# B cell CD19 is transferred between immune cells in mice and humans

**DOI:** 10.1038/s41467-026-75534-3

**Published:** 2026-07-29

**Authors:** Jasmin Ochs, Pia Schweineberg, Jacqueline Thode, Alica Blenkle, Leila Husseini, Matthias Klein, Tobias Bopp, Patrick Schindler, Friedemann Paul, Martin S. Weber

**Affiliations:** 1 Department of Neurology, University Medical Center, Göttingen, Germany; 2 Fraunhofer Institute for Translational Medicine and Pharmacology, Göttingen, Germany; 3 Institute of Neuropathology, University Medical Center, Göttingen, Germany; 4 Institute of Immunology, University Medical Center, Mainz, Germany; 5 Research Center for Immunotherapy (FZI), University Medical Center Mainz, Mainz, Germany; 6 Department of Neurology with experimental Neurology, Charité, Berlin, Germany; 7 Max Delbrueck Center for Molecular Medicine and Charité - Universitätsmedizin Berlin, Berlin, Germany

**Keywords:** Immunosuppression, Membrane fusion, Multiple sclerosis, Neuroimmunology, Translational immunology

## Abstract

When immune cells interact, they frequently exchange membrane-bound antigens. Our evolving understanding of these processes challenges the cellular specificity of lineage markers and therapeutic monoclonal antibodies. By using mouse and human B-T cell co-cultures, we report that CD19, an assumingly exclusive B cell marker, is transferred via trogocytosis when B cells activate T cells. In a B cell-driven model of experimental autoimmune encephalomyelitis, CD19^+^ T cells expand and show enhanced features of activation, differentiation, and encephalitogenic potential ex vivo. Additionally, co-transfer of CD19 and functional IgM from B cells results in the gain of B cell function by T cells. In patients with chronic central nervous system (CNS) demyelination, CD19^+^ T cells display a pro-inflammatory phenotype and are concomitantly depleted by inebilizumab, an approved anti-CD19 antibody, which raises important considerations for the therapeutic use of monoclonal antibodies overall. Finally, we report that myeloid cells acquire CD19 and functional IgM after phagocytosis of apoptotic B cells and thereby gain functional B cell properties. These findings highlight the commonness of membrane and antigen-transfer between cells, resulting in transmission of cellular function.

## Abstract (web summary)

Trogocytosis enables the transfer of membrane-bound antigens during pathogenic B-T cell interactions. Here, the authors utilize mouse and human cell models and demonstrate that the B cell marker CD19 is transferred, along with other B cell molecules, to T cells and myeloid cells. Importantly, the transferred molecules can be functional in the receiving cells and serve as a marker for B cell-activated T cells, resulting in a mainly pathogenic phenotype in CNS inflammation”.

## Introduction

The trogocytotic transfer of membrane and membrane-bound molecules during cell-cell interactions is a well-established process^1^. In the case of antigen presenting cells (APC) and T cells, trogocytosis is described as T cell receptor (TCR)-mediated endocytosis. After the formation of an immunological synapse, T cells internalize their TCR which is still bound to the major histocompatibility complex (MHC) class II-peptide complex of the APC^2^. Thereby, the T cell rips out, internalizes, and reexpresses the MHC II-peptide complex and, to a certain degree, the membrane and membrane-bound molecules surrounding the MHC II on the donor cell. Due to this process, MHC II is the most established transferred molecule. Its transfer can be considered specific and T cells can even utilize it to activate other T cells^3^. However, the transfer of the MHC II-surrounding membrane-bound molecules seems unspecific and largely relies on the donor cells and their expression patterns.

In our recent work, we showed that CD20, a B cell lineage marker, is transferred from B to T cells in an unspecific manner^4^. We proved that CD20^+^ T cells mainly develop via trogocytosis during pathogenic B cell-T cell interactions. The trogocytotic transfer of CD20 therefore marked T cells as recently B cell-activated T cells. CD20^+^ T cells present with a proinflammatory, most likely pathogenic phenotype and were shown to be increased in untreated multiple sclerosis (MS) patients and animals with experimental autoimmune encephalomyelitis (EAE)^4,5^. In MS and other inflammatory diseases, anti-CD20 monoclonal antibodies, such as rituximab, ocrelizumab, or ofatumumab, exhibit a resounding treatment effect^6–9^. The trogocytotic transfer of CD20 makes recently B cell-activated, proinflammatory T cells accessible to depletion by anti-CD20 antibodies which proved therapeutically beneficial^4,10,11^. This unspecific antigen transfer and its consequences in disease therapy opens up new opportunities: First, T cells might obtain a gain of function from acquired molecules; secondly, molecules found on T cells can lead to the identification of their recent interaction partners via molecular tracing; and lastly, the acquisition of membrane-bound antigens can make T cells accessible to therapeutic antibodies that were not originally designed to target them.

Accordingly, the focus here is to investigate how common membrane-bound antigen transfer is between immune cells, to understand what drives trogocytotic exchange and to elucidate whether this phenomenon is restricted to specific immune cells or molecules. Hence, we analyze the trogocytotic transfer of CD19, another B cell lineage marker, from B to T cells and explicate the consequences of this transfer, such as the concomitant transfer of neighboring membrane-bound molecules, resulting in a T cell gain of functional B cell properties. In addition, we demonstrate that membrane-embedded CD19 can also be transferred from B cells to phagocytes via efferocytosis, which again results in a concomitant transfer of functional B cell properties to phagocytes. Consequently, CD19 positivity renders both T cells and phagocytes accessible to anti-CD19 antibodies, resulting in their depletion. Since the monoclonal anti-CD19 antibody inebilizumab (INE) was recently approved for neuromyelitis optica (NMOSD) therapy^12,13^, knowledge about the transfer of CD19 and the added depletion of cells that are not B cells, might have a considerable impact for this novel treatment.

## Results

### CD19^+^ T cells exist in mouse and human, but CD19 is not endogenously expressed

**Fig. 1:**
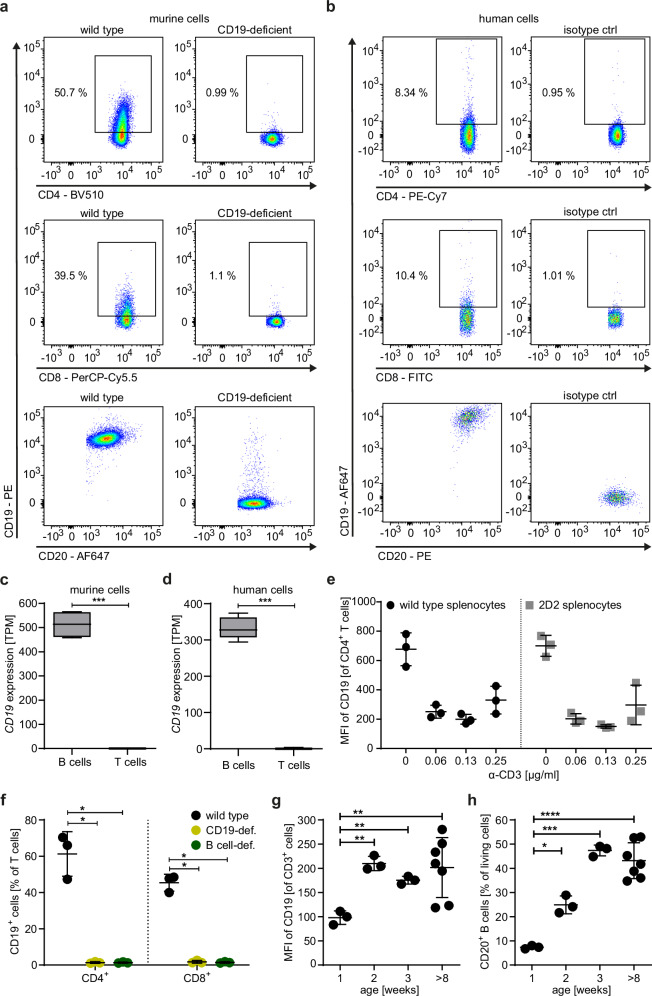
CD19 can be found on T cells but is not endogenously expressed. **a, b** Representative flow cytometric staining of CD19 on CD4^+^ and CD8^+^ T cells and B cells isolated from **a** wild type and CD19-deficient CD19-cre mice or **b** healthy control peripheral blood mononuclear cells (PBMC). **c, d** Bulk RNA sequencing of fluorescence-activated cell (FACS)-sorted B and T cells for the *CD19* transcript shown as TPM, reads per kilobase of transcript per million reads mapped; **c** *n* = 3 mice for B cells and 8 mice per T cells, analyzed via two-tailed unpaired Student’s t-test with Welch’s correction and Bonferroni correction for multiple testing; **d** *n* = 4 healthy human donors; one set of B and T cells per mouse or human, analyzed via two-tailed Mann-Whitney test with Bonferroni correction for multiple testing; displayed as box plot; **e** Mean fluorescence intensity (MFI) of CD19 of CD4^+^ T cells of splenocyte cultures isolated from wild type mice or myelin oligodendrocyte glycoprotein (MOG)_35–55_ peptide T cell receptor (TCR) transgenic 2D2 mice stimulated with anti-CD3/anti-CD28 antibodies for 48 h; *n* = 3 wells per group. **f** Flow cytometric analysis of CD19 on CD4^+^ and CD8^+^ T cells from the spleens of wild type, CD19-deficient CD19-cre, and B cell-deficient µMT mice; *n* = 3 per group; analyzed via Brown-Forsythe and Welch ANOVA with Dunnett’s T3 multiple comparisons test. **g** MFI of CD19 of CD3^+^ T cells and **h** percentage of CD20^+^ B cells in murine spleens during development; *n* = 3 mice for groups 1-3 weeks and 7 mice for the <8 group; analyzed via Brown-Forsythe and Welch ANOVA with Holm-Sidak’s multiple comparisons test. All figures are representative of **a, b, e**–**h** or pooled from **c, d** 2-4 independent experiments; data displayed as means ± SD; box plots are min to max with means ± SD; *=*p* < 0.05; **=*p* < 0.01; ***=*p* < 0.001; ****=*p* < 0.0001.

To find out whether CD19 is trogocytotically transferred from B to T cells, we first confirmed the existence of CD19^+^ T cells in mice and humans. CD19-deficient CD19-cre mice were used to distinguish the dimly positive from the negative cells. We discovered high amounts of CD19^+^CD4^+^ and CD8^+^ T cells with slightly higher amounts of CD19 on CD4^+^ T cells (Fig. 1a). To see if similar findings can be discovered in humans, we analyzed peripheral blood mononuclear cells (PBMC) from healthy controls (Fig. 1b). The positive signal was differentiated from the negative via isotype control antibody staining. Human T cells showed a lower but still substantial amount of CD19^+^ T cells in the CD4^+^ and CD8^+^ T cell populations. To examine whether T cells received their CD19 from B cells or endogenously expressed it, we purified murine and human T cells and analyzed them via bulk RNA sequencing (RNA-seq) for their expression of the *CD19* transcript (Fig. 1c, d). B cells were equally isolated and served as positive control. Purity of the cells was controlled by examining them for their expression of the *CD3e* (for T cells) and *MS4A1* (CD20; for B cells) transcripts (Supplementary Fig. 1a, c-e). The RNA-seq analysis revealed that neither murine nor human T cells are able to endogenously express *CD19*. Along these lines, direct T cell stimulation in splenocyte cultures from wild type and myelin oligodendrocyte glycoprotein (MOG)_35–55_ TCR transgenic 2D2 mice showed that T cells alone are unable to become CD19 positive (Fig. 1e and Supplementary Fig. 1h). The trogocytotic transfer of CD19 to T cells can be further implied since mice that either have no B cells (µMT) or no CD19 on their B cells (CD19-cre) also have no CD19^+^ T cells (Fig. 1f and Supplementary Fig. 1i–k). In addition, in the development of mice, T cells first become CD19 positive in the spleen at 2 weeks of age (Fig. 1g and Supplementary Fig. 1l), which coincides with the arrival of the first B cells from the bone marrow (Fig. 1h). These data demonstrate that CD19^+^ T cells exist and that they require B cells for their development.

### CD19 is trogocytotically transferred from B cells to T cells during B cell-T cell interaction

**Fig. 2:**
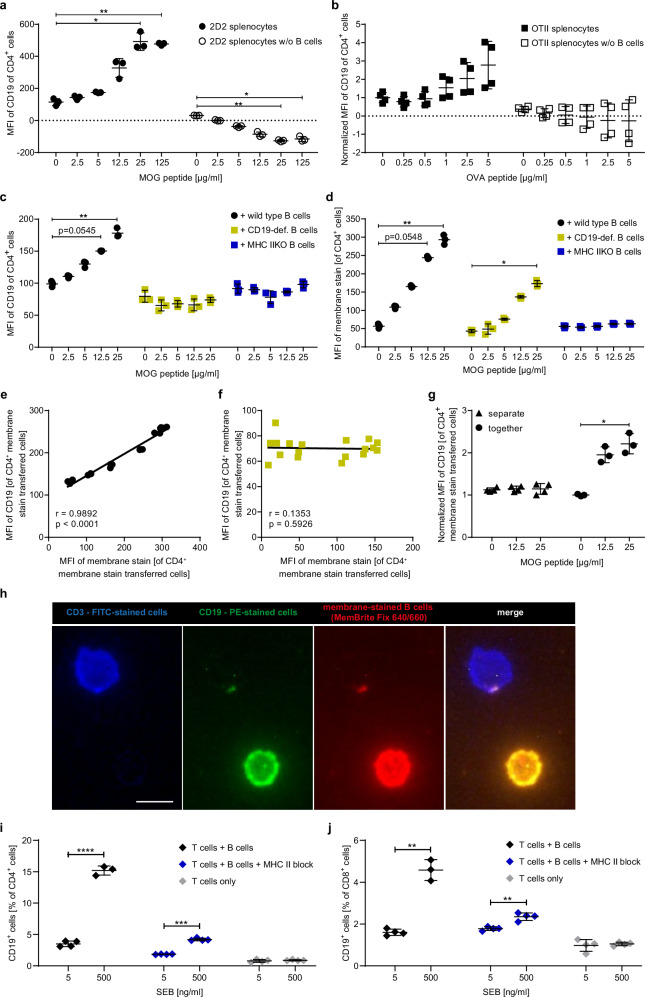
Development of CD19^+^ T cells requires direct contact of T and B cells and occurs via trogocytotic transfer of CD19. **a** Splenocyte culture with/without B cells and myelin oligodendrocyte glycoprotein (MOG)_35–55_ T cell receptor transgenic 2D2 T cells with MOG_35–55_ peptide. Mean fluorescence intensity (MFI) of CD19 on T cells. **b** Splenocyte culture with/without B cells and ovalbumin (OVA)_329–337_ peptide TCR transgenic T cells with OVA_329-337_ peptide. Normalized data of the MFI of CD19 on T cells; data normalized to the mean of 0 µg/ml. **c**–**f** Coculture of membrane-stained (mst) B cells from wild type, CD19-deficient, or MHC IIKO mice and 2D2 T cells stimulated with MOG_35–55_ peptide. **c**, MFI of CD19 on T cells. **d** MFI of mst on T cells. **a**–**d** Kruskal-Wallis test with Dunn’s multiple comparisons test; *n* = **a, c, d**, 3 wells and **b** 4 wells per group. **e** Correlation of the MFI of CD19 and mst on T cells after coculture with mst wild type B cells. **f** Correlation of the MFI of CD19 and mst on T cells after coculture with mst CD19-deficient B cells. **e, f** Two-tailed Spearman correlation; *n* = 3 wells per condition. **g** Transwell and nonseparated coculture of mst B and 2D2 T cells. MFI of CD19 of T cells; normalized to the mean of 0  µg/ml of the separated culture; Kruskal-Wallis test with Dunn’s multiple comparisons test; *n* = 3 wells (separated), 4 wells (together) per group. **h** Fluorescence microscopy after coculture: Mst B cells (red), CD3 (blue), and CD19 (green); scale bare: 10 µm. **i, j** Peripheral blood mononuclear T and B cell coculture or T cells only or B cells preincubated with anti-MHC class II (MHC II block) with Staphylococcal enterotoxin B (SEB); *n* = 4 wells per group (T cells + B cells 500 ng/ml: *n* = 3 wells). Analysis of CD19 on **i**, CD4^+^ or **j**, CD8^+^ T cells; two-tailed, unpaired Student’s t-test with Welch’s correction. All figures are representative of **a, c**–**f, h**–**j** or pooled from **b, g** at least two independent experiments; data is displayed as means ± SD; *=*p* < 0.05; **=*p* < 0.01; ****=*p* < 0.0001.

To determine whether CD19 is transferred during B cell-T cell interaction, we performed splenocyte cultures with cells from MOG_35-55_ TCR transgenic 2D2 mice stimulated with MOG_35-55_ peptide (Fig. 2a). Thereby, B cells can interact with T cells by presenting MOG_35-55_ peptide via MHC II. Afterward, we analyzed T cells via flow cytometry. The amount of CD19 on T cells displayed via the mean fluorescence intensity (MFI) increased concentration-dependently in the presence B cells. In splenocyte cultures without B cells the amount of CD19 on T cells decreased concentration-dependently. This was observed, even though both cultures showed similar T cell proliferation and activation (Supplementary Fig. 2a, b). The same tendency could be observed in splenocyte cultures with and without B cells from ovalbumin (OVA)_329–337_ peptide TCR transgenic OTII mice stimulated with OVA_329-337_ peptide (Fig. 2b and Supplementary Fig. 2c, d), making this finding antigen non-specific. To explicitly confirm that CD19 is transferred from B cells via trogocytosis, we performed B cell-T cell cocultures with 2D2 MOG_35-55_ TCR transgenic T cells and either wild type, CD19-deficient CD19-cre, or MHC IIKO B cells (Fig. 2c). An increase in CD19 on T cells could be observed only in cultures with CD19-expressing wild-type B cells. Cultures with CD19-deficient B cells showed no increase of CD19 on T cells, despite being similarly activated and proliferated (Supplementary Fig. 2e, f). In MHC IIKO B cell cultures, B cells were unable to interact with T cells in an antigen-presenting manner. Therefore, T cells from those cultures showed neither proliferation nor activation and did not become CD19 positive. To see if these results can be linked with trogocytosis where antigens are transferred due to the membrane transfer, we stained the various B cells with a membrane stain prior to co-culture. We could observe the transfer of stained B cell membrane to T cells in the co-cultures with wild-type and CD19-cre B cells. However, no transfer of either CD19 or membrane stain could be observed in co-cultures with MHC IIKO B cells, due to missing cell-cell interactions (Fig. 2d). That also confirms that the membrane stain cannot leak from B cells to T cells to unspecifically stain T cells in this setup. The membrane stain has to be actively transferred during cell-cell interactions. A correlation analysis revealed that in co-cultures with wild-type B cells the amount of membrane stain transferred to T cells highly significantly correlated to the amount of CD19 found on T cells (Fig. 2e). No correlation could be found between the transferred membrane stain and CD19 on T cells of CD19-def. CD19-cre B cell cocultures (Fig. 2f). Using a transwell coculture system, where B cells are separated from T cells via a membrane that still allows for the exchange of soluble factors, we confirmed that for the transfer of CD19 direct B cell-T cell interaction is crucial. When separated this way, T cells did not become CD19 positive nor were they activated (Fig. 2g and Supplementary Fig. 2g), confirming that CD19^+^ T cells only develop in direct cellular contact with B cells. Via fluorescence microscopic analysis of B and T cells after co-culture, we could visualize the transfer of a CD19-containing B cell membrane fragment to a T cell (Fig. 2h). Next, we investigated whether this transfer can be found in human cells. Therefore, we performed human B cell-T cell co-cultures stimulated with the bacterial superantigen Staphylococcus enterotoxin B (SEB) and also blocked this superantigen-driven interaction with an MHC II block (Fig. 2i, j and Supplementary Fig. 2h, i). The SEB-driven interaction led to a significant increase in CD19^+^ T cells, which could be partially blocked by an MHC II block. These results confirm that CD19 is trogocytotically transferred from B cells to T cells in mice and humans.

### The frequency of CD19^+^ T cells is increased in an inflammatory context

**Fig. 3:**
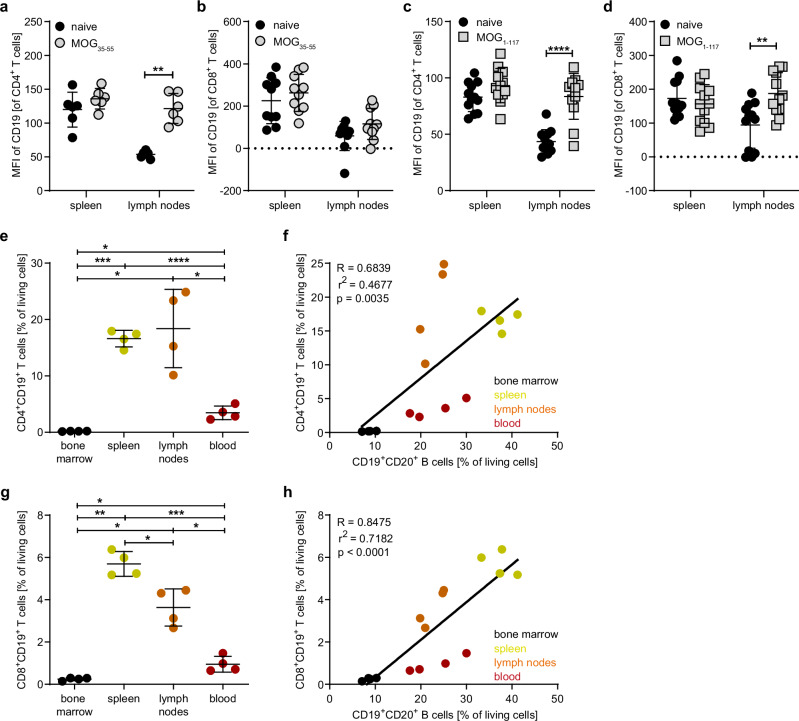
CD19^+^ T cells expand in EAE and are more prominent in B cell-rich compartments. **a**–**d** Mice were immunized with **a, b** MOG_35–55_ peptide or **c, d** conformationally folded MOG_1-117_ protein. Spleen and immunization draining lymph nodes were analyzed for their mean fluorescence intensity (MFI) of CD19 on **a, c** CD4^+^ or **b, d** CD8^+^ T cells; *n* = **a** 6; **b** 10; **c, d** 12 mice per group; analyzed via two-tailed Mann-Whitney test. **e, g** Flow cytometric analysis of CD19^+^ T cells in bone marrow, spleen, inguinal lymph nodes, and blood of wild type mice; *n* = 4 mice per group; analyzed via Brown-Forsythe and Welch ANOVA with Holm-Sidak’s multiple comparisons test. **f**, **h** two-tailed Pearson correlation analysis of CD19^+^ T cells and B cells in bone marrow, spleen, inguinal lymph nodes, and blood of wild type mice; *n* = 4 mice per group. All figures are representative of **e**–**h** or pooled from **a**–**d** 2-3 independent experiments; data is displayed as means ± SD; *=*p* < 0.05; **=*p* < 0.01; ***=*p* < 0.001; ****=*p* < 0.0001.

In inflammatory diseases such as MS and NMOSD, pathogenic B cell-T cell interactions are more prominent, evidenced by the higher number of CD20^+^ T cells detectable in patients with MS and mice with EAE. This observation suggests that the CD19^+^ T cell population should also expand in these diseases. Therefore, we immunized wild-type mice with MOG_35-55_ peptide (B cell-independent model) or MOG_1-117_ protein (B cell-involving model) and discovered an increase of CD19^+^ T cells in the immunization draining lymph nodes, comparable to that found for CD20^+^ T cells^4^ (Fig. 3a–d). Of note, the CD19 increase on T cells was higher in the B cell-involving model, indirectly confirming that pathogenic T cells are activated by B cells. Comparing blood samples from MS patients to healthy controls was not as conclusive (Supplementary Fig. 3a). To explain this discrepancy, we analyzed various murine immune cell compartments and the blood of several wild-type mice. We discovered that the highest amounts of CD19^+^ T cells were detected in the spleen and lymph nodes and that the amount of CD19^+^ T cells per organ strongly correlated with the amount of B cells present (Fig. 3e–h and Supplementary Fig. 3b–d). This result indicates that the blood of patients with MS might not sufficiently represent the effects found in the immunization draining lymph nodes of EAE mice. These results show that CD19^+^ T cells expand in EAE and indicate that this most probably also occurs in patients with MS, but would not easily be visible in the blood and may rather occur in secondary lymphoid organs.

### CD19 on T cells is tightly associated with differentiation, activation, and enhanced pathogenic properties

**Fig. 4:**
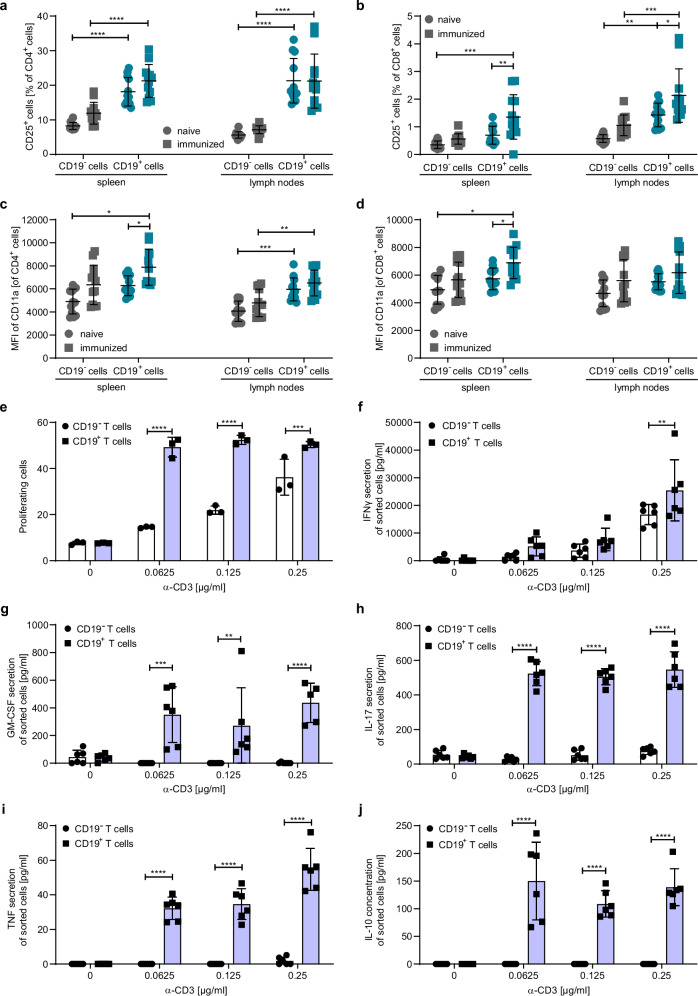
CD19^+^ T cells show an enhanced expression of activation markers, adhesion molecules, and cytokines. **a**–**d** Flow cytometric analysis of the spleen and immunization draining lymph nodes of naïve or MOG_1-117_ protein–immunized mice; *n* = 12 mice per group; analyzed via two-way ANOVA with Tukey’s multiple comparisons test. The expression of **a, b** CD25 (%) and **c**, **d** CD11a (mean fluorescence intensity, MFI) were evaluated on CD19^−^ and CD19^+^ CD4^+^ and CD8^+^ T cells. **e** Flow cytometric analysis of T cell proliferation; *n* = 3 wells per group; analyzed via 2-way ANOVA with Sidak’s multiple comparisons test. **f-j** ELISA-based cytokine analysis for **f** IFN-γ, **g** GM-CSF, **h** IL-17, **i** TNF, and **j** IL-10 of the supernatant of fluorescence-activated cell sorted CD19^+^ and CD19^−^ T cells from the spleens and inguinal lymph nodes of MOG_35–55_ peptide–immunized mice stimulated with anti-CD3/anti-CD28 antibodies; *n* = 6 wells (apart from **g** CD19^+^ T cells 0.25: *n* = 5 wells) per group analyzed via 2-way ANOVA with Sidak’s multiple comparisons test. All figures are pooled from **a**–**d, f**–**j** or representative of **e** 2-3 independent experiments; data is displayed as means ± SD; *=*p* < 0.05; **=*p* < 0.01; ***=*p* < 0.001; ****=*p* < 0.0001.

After discovering that CD19^+^ T cells expand in inflammatory diseases similar to CD20^+^ T cells, we then analyzed whether they also exhibit the same pathogenic potential. Therefore, we compared CD19^+^ T cells to CD19^-^ T cells under naive and EAE-induced conditions ex vivo. CD19^+^ T cells expressed significantly higher amounts of early (CD69) and late (CD25) T cell activation markers compared to CD19^−^ T cells (Fig. 4a, b and Supplementary Fig. 4a, b). In addition, CD19^+^ T cells expressed significantly higher quantities of the adhesion molecules CD11a (LFA-1) and CD49d (integrin α4) than their CD19^-^ counterparts (Fig. 4c, d and Supplementary Fig. 4c, d). To examine the cytokine expression of CD19^+^ T cells, we isolated CD19^+^ and CD19^−^ T cells of EAE-induced mice and restimulated them with anti-CD3/CD28 antibodies. CD19^+^ T cells showed a significantly higher proliferation compared to CD19^-^ T cells (Fig. 4e). While there were only minor but significant increases in the expression of IFNγ, the expression of the proinflammatory cytokines IL-17, TNF, and GM-CSF, the expression of IL-2 and the anti-inflammatory cytokines IL-10 and IL-4 were profoundly increased, indicating that CD19^+^ T cells are highly differentiated compared to CD19^-^ T cells (Fig. 4f–j and Supplementary Fig. 4e, f). Concerning the composition of secreted cytokines, both pro- and anti-inflammatory cytokines were more abundant in CD19^+^ T cells; however, the production of proinflammatory cytokines was considerably higher than the anti-inflammatory one. Since CD19^+^ T cells showed significantly higher proliferation, we adjusted cytokine secretion to the cell proliferation to factor in the impact of the increased cell number and could still detect the same significant increase in cytokine secretion (Supplementary Fig. 4g–l). Therefore, we conclude that CD19^+^ T cells are highly activated and differentiated cells with a mainly proinflammatory phenotype and properties that could potentially promote pathogenic CNS infiltration.

### CD19^+^ T cells acquire B cell properties

**Fig. 5:**
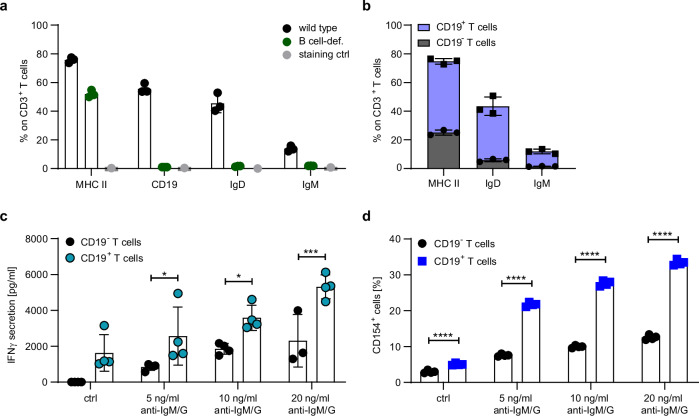
CD19^+^ T cells can be activated by B cell-stimulation. **a** Flow cytometric analysis of B cell marker on CD3^+^ T cells from the spleens of wild type and B cell-deficient µMT mice; *n* = 3 mice per group; either isotype control or fluorescence minus one served as staining control. **b** B cell marker positive cells from **a** were analyzed for their double positivity with CD19; *n* = 3 mice per group. **c** ELISA-based cytokine analysis for IFN-γ of the supernatant of fluorescence-activated cell sorted CD19^+^ and CD19^−^ T cells from the spleens of naive mice stimulated with anti-IgM/IgG F(ab′)2 fragment; *n* = 4 wells (apart from CD19^-^ T cells 20 ng/ml: *n* = 3 wells) per group analyzed via 2-way ANOVA with Sidak’s multiple comparisons test. **d** Flow cytometric analysis of fluorescence-activated cell sorted CD19^+^ and CD19^−^ T cells from the spleens of naive mice stimulated with anti-IgM/IgG F(ab′)2 fragment; *n* = 4 wells per group analyzed via 2-way ANOVA with Sidak’s multiple comparisons test. All figures are representative of 2–3 independent experiments; data is displayed as means ± SD; *=*p* < 0.05; ***=*p* < 0.001; ****=*p* < 0.0001.

Further analyzing CD19^+^ T cells, we found that other markers considered B cell-specific (IgD, IgM) are concomitantly transferred to T cells and occur there mainly in a double positive setting with CD19 (Fig. 5a, b and Supplementary Fig. 5a–d). MHC II has already been described as functional on T cells^14^; but to determine whether the B cell-specific antigens are also functional on T cells, we stimulated purified CD19^+^ and CD19^-^ T cells separately with an anti-IgM/IgG Fab-fragment. We observed a concentration-dependent stimulation of T cells with this B cell receptor-specific stimulation via the secretion of IFNγ and the expression of CD154 (CD40 Ligand) (Fig. 5c, d) that occurred predominantly in the CD19^+^ T cell population. In this crucial set of experiments, we could accordingly establish that other B cell molecules besides CD19 are transferred to T cells and that these membrane-embedded molecules are functional in the receiving T cell.

### Human CD19^+^ T cells display a proinflammatory phenotype with enhanced pathogenic properties

**Fig. 6:**
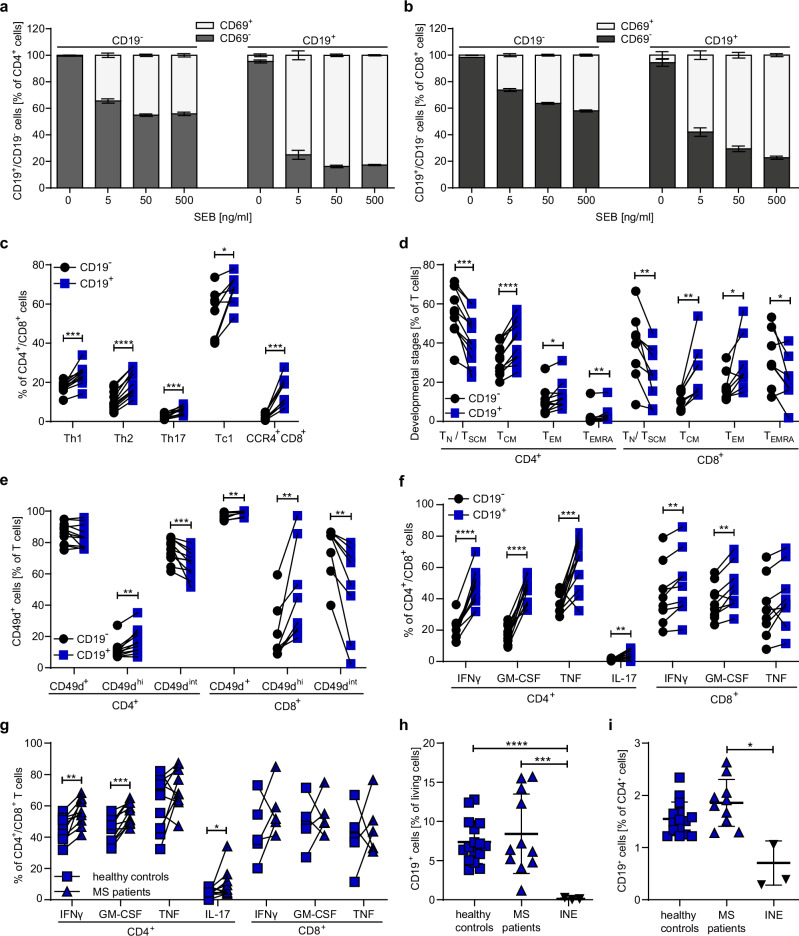
In healthy controls and patients with MS, CD19^+^ T cells display a more activated, mature phenotype. **a, b** Coculture of T and B cells from peripheral blood mononuclear cells (PBMC) of healthy donors with Staphylococcal enterotoxin B (SEB) for 48 hours; *n* = 4 wells (apart from 500 ng/ml: *n* = 3 wells) per condition. Normalized flow cytometric data of **a** CD4^+^CD19^+^ and CD4^+^CD19^−^ T cells (P = 0.006 in 0 µg/ml and *P* < 0.0001 in all other concentrations comparing CD19^+^ with CD19^−^), and **b** CD8^+^CD19^+^ and CD8^+^CD19^−^ T cells (*P* = 0.0208 in 0 µg/ml and *P* < 0.0001 comparing CD19^+^ with CD19^−^ in all concentrations) for CD69 expression; analyzed via 2-way ANOVA with Sidak’s multiple comparisons test. **c**–**f** Flow cytometric analysis of healthy control PBMCs. Comparison of CD19^+^ versus CD19^−^ T cells in **c** differentiation (Th1, Th2, Th17, *n* = 11; Tc1, CCR4^+^CD8^+^, *n* = 8), **d** developmental state [CCR7^+^CD45RO^− ^= T_N_/T_SCM_ (naive and stem cell–like memory), CCR7^+^CD45RO^+^ = T_CM_ (central memory), CCR7^−^CD45RO^+^ = T_EM_ (effector memory), CCR7^−^CD45RO^− ^= T_EMRA_ (terminally differentiated); *n* = 10 CD4^+^; *n* = 6 CD8^+^], **e** adhesion capability (integrin α4 = CD49d; *n* = 10 CD4^+^; *n* = 6 CD8^+^), and **f** cytokine production (IFN-γ, GM-CSF, TNF, and IL-17; *n* = 11 CD4^+^; *n* = 9 CD8^+^); analyzed by two-tailed Wilcoxon matched-pairs signed rank test. **g** Flow cytometric analysis of cytokine production (IFN-γ, GM-CSF, TNF, and IL-17) of CD19^+^ T cells from healthy controls vs MS patients; *n* = 10 CD4^+^; *n* = 5 CD8^+^; analyzed by two-tailed Wilcoxon matched-pairs signed rank test. **h, i** Flow cytometric analysis of **h** CD19^+^ B cells and **i** CD19^+^CD4^+^ T cells of healthy controls (*n* = **h** 16; **i** 15), untreated RRMS (*n* = **h** 11; **i** 10), and Inebilizumab (INE)-treated NMOSD patient PBMCs (*n* = 4); analyzed via Brown-Forsythe and Welch ANOVA test via Games-Howell’s multiple comparison test after analysis for normality via D’Agostino & Pearson test. All figures are representatives of **a, b** or pooled from **c**–**i** 2-3 independent experiments; data is displayed as means ± SD; *=*p* < 0.05; **=*p* < 0.01; ***=*p* < 0.001; ****=*p* < 0.0001.

To investigate if the pathogenic potential of CD19^+^ T cells displayed in mice holds true in humans, we performed human B cell-T cell cocultures and discovered that CD19^+^ T cells expressed the early activation marker CD69 earlier and to a higher amount than CD19^-^ T cells (Fig. 6a, b). Subsequently, we examined human CD19^+^ T cells unstimulated ex vivo using PBMCs from untreated MS and NMOSD patients and healthy controls. We compared CD19^+^ with CD19^-^ of both CD4^+^ and CD8^+^ T cells and found that CD19^+^ T cells have a higher quantity of differentiated T helper (Th)1 (CD4^+^CXCR3^+^), Th2 (CD4^+^CCR4^+^), Th17 (CCR4^+^CCR6^+^), Tc1 cytotoxic T cells (CD8^+^CXCR3^+^), and CD8^+^CCR4^+^ (Fig. 6c, Supplementary Fig. 6a, and Supplementary Table 1). Concerning maturation both in healthy controls and MS patients, CD19^+^ T cells presented significantly lower frequencies of immature, naïve and stem cell–like memory cells (CCR7^+^CD45RO^−^), while they consequently contained significantly higher frequencies of central memory (CCR7^+^CD45RO^+^) and effector memory T cells (CCR7^−^CD45RO^+^) (Fig. 6d and Supplementary Fig. 6b). Furthermore, CD19^+^ T cells exhibited significantly higher quantities of the adhesion molecule CD49d (Fig. 6e and Supplementary Fig. 6c) and contained significantly more IFNγ–, GM-CSF–, TNF, and IL-17–producing cells (Fig. 6f and Supplementary Fig. 6d). In patients with MS, the CD19^+^ T cell population contained even higher amounts of proinflammatory cytokine-secreting cells than in age- and sex-matched healthy controls, but no significant changes in the differentiation, maturation, or adhesion molecule expression could be detected (Fig. 6g and Supplementary Fig. 6e–g). In NMOSD patients, B cell depletion could be observed under anti-CD19 antibody (inebilizumab) treatment (Fig. 6h). A hiding of B cells due to an inability to stain CD19 after anti-CD19 treatment could be excluded by CD20 staining (Supplementary Fig. 6h). In addition, the percentage of CD19^+^CD4^+^ T cells significantly decreased, while no changes could be measured in the CD8^+^CD19^+^ T cell population (Fig. 6i and Supplementary Fig. 6i). To conclude, human CD19^+^ T cells display a highly activated and differentiated, mainly proinflammatory phenotype with properties that could potentially further pathogenic CNS infiltration and can be depleted by inebilizumab.

### CD19^+^ monocytes exist in mice and humans, however CD19 is not endogenously expressed

**Fig. 7:**
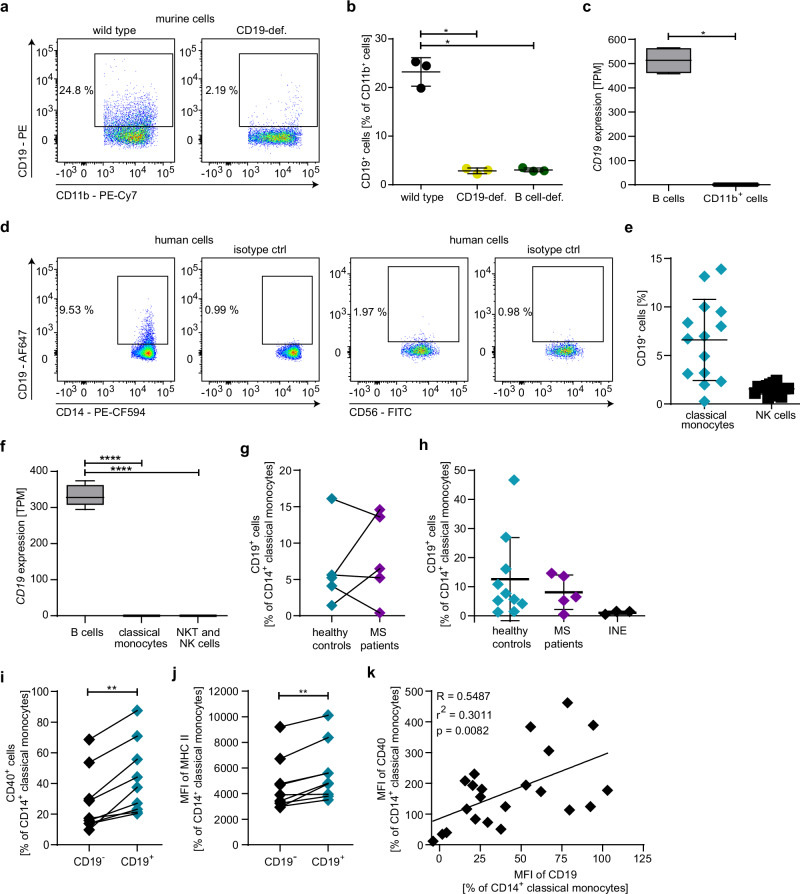
CD19 is present on monocytes even though they cannot endogenously express it. **a** Representative staining of CD19 on monocytes from spleens of wild type and CD19-deficient mice. **b** Analysis of CD19 on CD11b^+^ monocytes from the spleens of wild type, CD19-deficient, and B cell-deficient mice; *n* = 3 mice per group; analyzed via Brown-Forsythe and Welch ANOVA with Holm-Sidak’s multiple comparisons test. **c** Bulk RNA sequencing of B cells (*n* = 4 mice) and CD11b^+^ monocytes (*n* = 3 mice) for the *CD19* transcript shown as TPM, reads per kilobase of transcript per million reads mapped; analyzed via two-tailed Wilcoxon signed-rank test and Bonferroni correction for multiple testing. **d** Representative staining of CD19 on classical monocytes (CM) and natural killer (NK) cells from human peripheral blood mononuclear cells (PBMC). **e** Analysis of CD19 on CMs and NK cells from PBMCs; *n* = 14 per group**. f** Bulk RNA sequencing of B cells (*n* = 5), CMs (*n* = 4), and NK cells/natural killer T (NKT) cells (*n* = 3) for the *CD19* transcript from healthy human controls; shown as TPM; analyzed via two-tailed, unpaired Student’s t test with Welch’s correction and Bonferroni correction for multiple testing. **g** Analysis of CD19^+^ cells of CMs of healthy control and MS patient PBMCs; *n* = 5 PBMC samples per group. **h** Analysis of CD19^+^CD14^+^ CMs of healthy control (*n* = 10), untreated RRMS (*n* = 5), and Inebilizumab (INE)-treated NMOSD patient PBMCs (*n* = 4); Kruskal-Wallis test with Dunn’s multiple comparisons test after analysis for normality via D’Agostino & Pearson test. **i**, **j** Analysis of human CD19^+^ versus CD19^−^ CMs from PBMCs of healthy controls for **i** CD40 (%), **j** MHC II (mean fluorescence intensity, MFI); *n* = 9; analyzed via two-tailed Wilcoxon matched-pairs signed rank test. **k** Two-tailed Pearson correlation analysis of the MFI of CD40 and CD19 on CM from healthy human controls; *n* = 22. All figures are representatives of **a, b, d** or pooled from **c, e**–**k** 2–3 independent experiments; displayed as means ± SD; box plots are min to max with means ± SD; *=p < 0.05; **=p < 0.01; ****=p < 0.0001.

Incidentally, not only T cells are positive for CD19, but we also discovered a dim CD19 signal on monocytes. Flow cytometric analysis of murine wild type CD11b^+^ monocytes revealed a significant portion of CD19^+^CD11b^+^ monocytes (Fig. 7a). The CD19 signal was verified using CD19-deficient CD19-cre mice and CD19^+^ monocytes do not occur in B cell-deficient µMT mice (Fig. 7b and Supplementary Fig. 7a), indicating the need of B cells for the CD19 positivity of monocytes. To analyze if murine CD19^+^CD11b^+^ monocytes are able to endogenously express CD19 or receive it from B cells, CD11b^+^ monocytes were isolated by FACS and bulk RNA sequencing was performed (Fig. 7c). B cells were equally isolated to serve as positive control. Purity of the populations was analyzed via the transcripts *MS4A1* (CD20, for B cells) and *Itgam-1* (CD11b, for monocytes) (Supplementary Fig. 1b, c). No transcripts of *CD19* could be detected in the CD11b^+^ monocyte samples, meaning that they do not endogenously express *CD19*. The same analyses were performed for human classical monocytes and natural killer (NK) cells. Human classical CD14^+^ monocytes (CM) showed a significant amount of CD19^+^ cells gated via isotype control antibodies (Fig. 7d, e and Supplementary Fig. 7b), while NK cells only exhibited a small percentage of CD19^+^ cells. The human cells were equally isolated and examined via bulk RNA sequencing, to determine if CM and NK cells can endogenously express *CD19* (Fig. 7f). The purity was analyzed by examining for the transcripts *MS4A1* (CD20, for B cells), *CD14* (for CM), and *NCAM-1* (CD56, for NK and NKT cells) (Supplementary Fig. 1e–g). No transcripts of *CD19* could be detected in CM and NK and NKT cells, meaning that neither do endogenously express *CD19*. The discovery of CD19^+^ CM, raised the question whether they might increase under inflammatory conditions, such as MS, however no conclusive changes could be observed (Fig. 7g and Supplementary Fig. 7c). Under anti-CD19 inebilizumab treatment, we found a decrease of CD19^+^ CM, implying their depletion by this monoclonal antibody (Fig. 7h). Interestingly, when compared, CD19^+^ CM showed a higher expression of CD40 and MHC II, marker associated with activation and phagocytosis, then CD19^-^ CM (Fig. 7i, j). The amounts of CD19 and CD40 on CM are strongly correlated (Fig. 7k). These results, together with the absence of CD19^+^ monocytes in B cell-deficient mice and their inability to endogenously express *CD19*, strongly indicate that monocytes must also receive CD19 from B cells.

### Phagocytic myeloid cells can acquire CD19 from B cells via efferocytosis

**Fig. 8:**
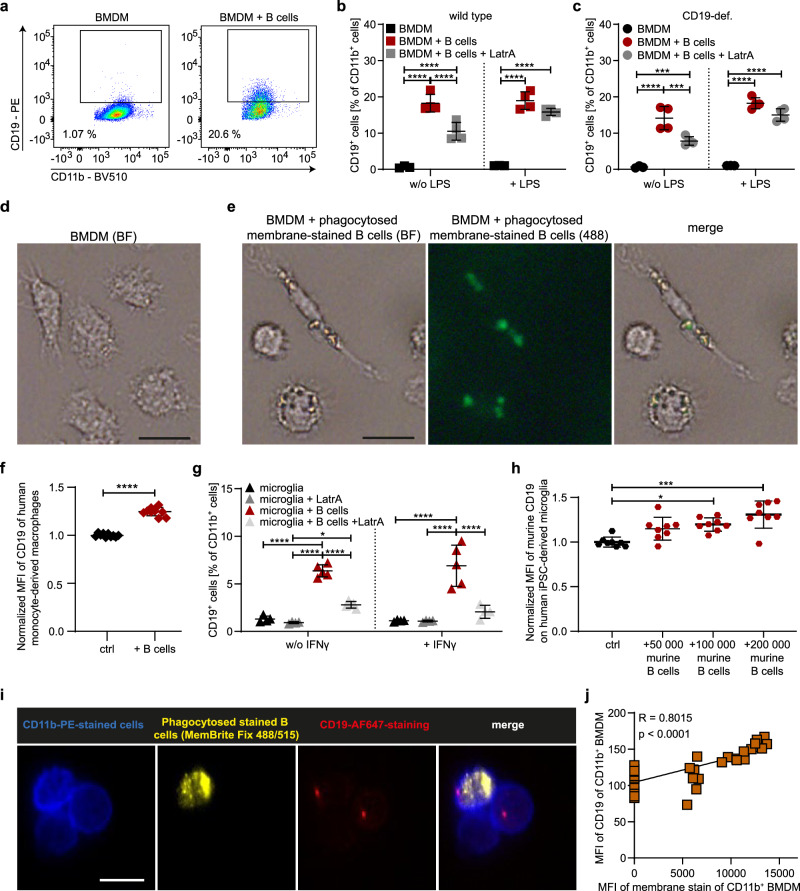
CD19 is transferred to phagocytes via efferocytosis. **a** Representative staining of CD19 on bone marrow derived macrophages (BMDM) with and without the absorption of apoptotic B cells. **b**, Analysis of CD19 on **b** wild type or **c** CD19-deficient BMDMs after the ingestion of apoptotic B cells with or without a prestimulation with LPS and with or without Latrunculin A (LatrA) as phagocytosis inhibitor; *n* = 4 wells (apart from BMDMs: *n* = 3 wells) per group; analyzed via 2-way ANOVA with Tukey’s multiple comparisons test. **d, e** Representative microscopic image of **d** BMDMs via brightfield (BF) and **e** BMDMs after the ingestion of apoptotic, membrane stained-B cells (MemBrite® Fix 488/515, green) in brightfield and fluorescence microscopy; scale bare measures 20 µm. **f** Flow cytometric analysis of CD19 on human monocyte-derived macrophages after the ingestion apoptotic B cells; *n* = 10 wells per group; normalized to the mean of the control group and analyzed via two-tailed, unpaired Student’s t-test with Welch’s correction. **g** Analysis of CD19 on microglia after the absorption of apoptotic B cells with or without a prestimulation with IFNγ and with or without LatrA as phagocytosis inhibitor; *n* = 4 wells (apart from + B cells and + B cells+LatrA w/o IFNγ; *n* = 5) per group; analyzed via 2-way ANOVA with Tukey’s multiple comparisons test. **h** Analysis of CD19 on human induced pluripotent stem cell (hiPSC)-derived microglia after the ingestion of murine apoptotic B cells; *n* = 8 per group; normalized to the mean of the control group and analyzed via Kruskal-Wallis test with Dunn’ multiple comparisons test. **i** Fluorescence microscopic examination of the efferocytotic transfer of CD19 to BMDMs. BMDMs stained with CD11b-PE (blue), ingested membrane-stained B cell particles (MemBrite® Fix 488/515, yellow), and CD19-AF647 (red); scale bare measures 20 µm. **j** Two-tailed Spearman correlation analysis of the mean fluorescence intensity of CD19 and membrane stain of BMDMs before and after the absorption of membrane-stained apoptotic B cells; *n* = 40. All figures are representatives of **a**–**e, g**–**i** or pooled from **f, h, j** 2-3 experiments; displayed as means ± SD; *=*p* < 0.05; ***=*p* < 0.001; ****=*p* < 0.0001.

We hypothesize that phagocytes, such as macrophages or microglia, could acquire membrane-bound antigens via efferocytosis, the clearance of apoptotic cells by phagocytes. Membrane recycling of ingested apoptotic cells, as observed in continuous efferocytosis, has high potential to lead to the re-expression of antigens^15^. To analyze whether CD19 is transferred that way, we generated bone marrow-derived macrophages (BMDM) and added apoptotic B cells to stimulate efferocytosis. BMDMs that ingested apoptotic B cells became significantly positive for CD19 (Fig. 8a, b). When we partly inhibited these processes via the phagocytosis inhibitor Latrunculin A (LatrA), the amount of CD19 on BMDMs after the culture was significantly lower. The phagocytotic capacity of BMDMs and its inhibition via LatrA were controlled with FITC-labeled Ovalbumin (Supplementary Fig. 7d). To prove that BMDMs actually absorbed apoptotic B cells, we stained B cells with a membrane stain before the assay and subsequently analyzed the BMDMs via flow cytometry and fluorescence microscopy (Fig. 8d, e and Supplementary Fig. 7e, f). This proved that apoptotic B cells are ingested due to the membrane stain signal found inside the BMDMs. In addition, LatrA also inhibited the absorption of apoptotic B cells by BMDMs, shown by the reduction of membrane stain signal (Supplementary Fig. 7e). To ensure that BMDMs did not simply start to express *CD19* due to an activation triggered by efferocytosis, we performed the same experiment with BMDMs generated from CD19-deficient mice, genetically unable to express *CD19*. We found the same increase in CD19 after stimulation with apoptotic B cells and its inhibition by LatrA (Fig. 8c). The phagocytotic capacity of CD19-deficient BMDMs and inhibition by LatrA were again controlled by FITC-labeled Ovalbumin (Supplementary Fig. 7g). To investigate if the same process occurs in humans, we isolated monocytes from PBMCs and differentiated them into human monocyte-derived macrophages (hMDM). We could similarly observe a significant increase in CD19 positivity after the ingestion of apoptotic human B cells (Fig. 8f and Supplementary Fig. 7h). Next, we wanted to test whether CNS-resident phagocytes were equally able to acquire CD19 via efferocytosis. Therefore, we generated murine primary microglia and stimulated them with apoptotic B cells. Microglia showed a significant positive signal for CD19 after the ingestion of apoptotic B cells, which could again be blocked by LatrA (Fig. 8g and Supplementary Fig. 7i). The phagocytotic capacity of microglia and inhibition by LatrA was controlled by FITC-labeled Ovalbumin (Supplementary Fig. 7j). To prove that microglia did not express CD19 due to the stimulation of efferocytosis, we performed the assay with apoptotic B cells from CD19-deficient CD19-cre mice and observed no increase in the CD19 signal of microglia (Supplementary Fig. 7k). Again, we investigated whether this process occurs in human microglia in a similar manner. Therefore, we generated human iPSC (induced pluripotent stem cell)-derived microglia and incubated them with murine apoptotic B cells (Fig. 8h and Supplementary Fig. 7l). We observed a murine CD19 signal on human iPSC-derived microglia, further proving the unspecificity of this process due to this xenogeneic transfer. To conclusively link the efferocytosis of apoptotic B cells with CD19^+^ phagocytes, we analyzed BMDMs after the stimulation with apoptotic, membrane-stained B cells via fluorescence microscopy (Fig. 8i). Internalized membrane-stained B cell fragments could be observed inside BMDMs, whereas a small dot of CD19 could be stained on the cell surface and persisted after the membrane-stained apoptotic B cell fragments were degraded. Additionally, we found a highly significant positive correlation between the amount of absorbed membrane stain with the CD19 signal on BMDMs after the efferocytosis of apoptotic B cells (Fig. 8j). Taken together these results prove that phagocytes can acquire CD19 via efferocytosis.

### CD19^+^ phagocytes can acquire B cell-properties

**Fig. 9:**
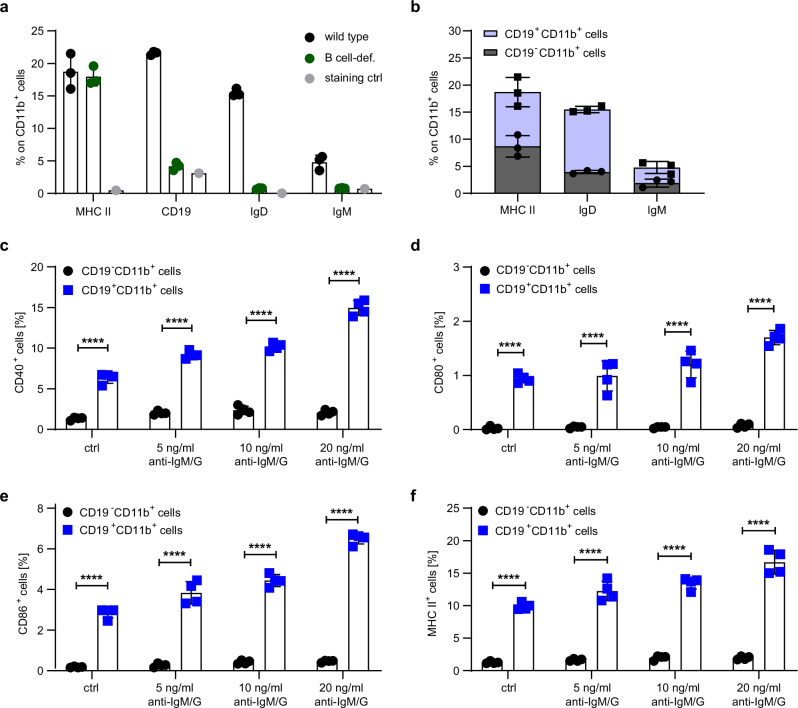
CD19^+^ myeloid cells can be stimulated via the B cell receptor. **a** Flow cytometric analysis of B cell marker on CD11b^+^ cells from the spleens of wild type and B cell-deficient µMT mice; n = 3 mice per group; either isotype control or fluorescence minus one served as staining control. **b** B cell marker positive cells from **a** were analyzed for their double positivity with CD19; *n* = 3 mice per group. **c**–**f** Flow cytometric analysis of fluorescence-activated cell sorted CD19^+^ and CD19^−^ CD11b^+^ cells from the spleens of naive mice stimulated with anti-IgM/IgG F(ab′)2 fragment; *n* = 4 wells per group analyzed via 2-way ANOVA with Sidak’s multiple comparisons test. All figures are representatives of 2–3 independent experiments; displayed as means ± SD; ****=*p* < 0.0001.

Based on the rather unspecific mechanism of efferocytosis, it seemed unlikely, that exclusively CD19 is transferred to phagocytes. In contrast, we found that other B cell-specific markers (IgD, IgM) are also transferred and found on the surface of phagocytes and that this occurs in association with the transfer of CD19 (Fig. 9a, b and Supplementary Fig. 8a–d). To find out, if the transferred B cell-specific antigens are also functional on phagocytes, we stimulated purified CD19^+^ and CD19^-^ CD11b^+^ cells separately with a B cell receptor-stimulating anti-IgM/IgG Fab-fragment. We could observe the activation of CD11b^+^ cells using this B cell-specific stimulation via an upregulation of the costimulatory molecules CD40, CD80, CD86, and MHC II (Fig. 9c–f and Supplementary Fig. 8e) in the population of CD19^+^ phagocytes. In conclusion, these experiments demonstrate that the efferocytotic transfer of B cell antigens to phagocytes is not restricted to CD19 and that the transferred molecules are functional in the receiving phagocytes.

## Discussion

The trogocytotic transfer of membrane-bound antigens between cells is an established process. It is described for a variety of immune cells, such as T cells^2^, B cells^16^, NK cells^17^, microglia^18^, and neutrophils^18^, and a plethora of molecules^19^. The trogocytotic transfer of the MHC II-peptide complex and MHC II-surrounding costimulatory molecules, such as CD86, from an APC via TCR-mediated endocytosis in the immunological synapse is one of the best characterized examples^2,19^. By expressing the MHC II-peptide complex on its surface, the recipient T cell acquires the ability to activate other T cells in an antigen-presenting manner^3^. Therefore, the T cell gains function due to the trogocytotic antigen transfer, while the donor cells lose part of their functions due to the relative decline of MHC II-peptide complexes on their surface.

With the work on MHC class II, we learned that the density and quantity of an antigen on the cell surface, in all likelihood, determine its probability to be transferred. In our previous work, we described the unspecific, trogocytotic transfer of CD20 from B to T cells during T cell activation^4^. CD19 is another antigen highly expressed on B cells, making its unspecific transfer highly likely. Our experiments proved that, just as with CD20, CD19 is transferred to T cells without directly leading to any detectable gain of function for the T cell. However, CD19 also marks T cells as recently B cell-activated T cells that exhibit a proinflammatory, potentially pathogenic phenotype. CD19^+^ T cells also show a high probability of being co-transferred with other B cell antigens such as IgD and IgM. These antigens proved to be functional on T cells, resulting in a T cell gain of B cell properties. Furthermore, transferred CD19 makes T cells accessible to anti-CD19 inebilizumab depletion. Having these results in mind, it has to be considered whether there is an overlap between the CD19^+^ and the CD20^+^ T cell populations. Both CD19 and CD20 are highly expressed on B cells during their development. A transfer of both antigens in the same trogocytotic process is therefore quite likely, leading to the possibility that inebilizumab would also partly deplete CD20^+^ T cells and anti-CD20 antibodies would additionally deplete parts of the CD19^+^ T cell population.

These considerations are of particular interest for inflammatory diseases such as MS, RA, and NMOSD. Our results show that an increase in pathogenic B cell-T cell interactions leads to an expansion of the CD19^+^ T cell population in EAE. CD19^+^ T cells exhibited an activated, mainly proinflammatory phenotype with an increased expression of proinflammatory cytokines. Their higher expression of adhesion molecules might impact the migration pattern of CD19^+^ T cells into the CNS in EAE/MS pathology. In the case of CD20^+^ T cells, an accumulation in the cerebrospinal fluid could be observed in MS patients. Whether the same holds true for CD19^+^ T cells appears quite likely but remains to be explored. For CD20^+^ T cells, their pathogenic potential and the therapeutic benefit of their depletion could be proven. Since CD19^+^ T cells strongly resemble CD20^+^ T cells, assuming them to be of equal pathogenic potential appears reasonable but requires further research. We could show that CD19^+^ T cells are indeed depletable by anti-CD19 antibodies and their depletion is very likely beneficial in NMOSD inebilizumab therapy.

However, the clinical impact of anti-CD19 depleting CD19^+^ T cells may be limited by the turnover of plasma membranes and thus the accessibility of membrane-embedded CD19 on T cells. In return, this aspect of membrane turnover could be an advantage in regard to immunosurveillance, as inebilizumab may also deplete CD19^+^ T cells raised to counteract infections. In previous work, we demonstrated that T cells completely replace their entire plasma membrane within 24–48 h and that the acquired CD19 and/or CD20 is internalized in this process^4^. As T cells cannot regenerate exchanged antigens, they are lost from the receiving cell surface 24–48 h after the transfer. Most antibodies against CD20 or CD19, such as ocrelizumab and inebilizumab, are applied 6-monthly intravenously, with studies planning to even widen the timeframe between applications. Accordingly, in these treatment regimens, CD19^+^ or CD20^+^ T cells may not be continuously depleted; on the other hand, both T cell populations may not redevelop in the absence of B cells and thus the relatively infrequent application of anti-CD20 or anti-CD19 may suffice to eradicate these recently B cell-activated T cell populations and to ensure their persisting absence. Whether regimens with shorter treatment intervals, such as subcutaneously applied anti-CD20 ofatumumab may differ in their ability to control pathogenic B-T cell interaction via the more frequent concomitant depletion of activated T cells remains to be examined.

In this work, we demonstrated the direct transfer of CD19 to CD4^+^ T cells. However, we revealed that a minor fraction of CD8^+^ T cells is also positive for CD19 and that both populations do not exist in mice genetically programmed to lack B cells. How CD8^+^ T cells obtain CD19 however remains elusive. The most probable hypothesis is its acquisition by T cells in a trogocytotic transfer during B cell-T cell interaction via the MHC I-peptide complex and the TCR. The trogocytotic transfer of the MHC I-peptide complex from APCs to CD8^+^ T cells is well described and an additional unspecific transfer of the surrounding membrane-bound molecules is therefore quite plausible^19^. However, the acquisition of the MHC I-peptide complex by CD8^+^ T cells is described to lead to fratricide, where other CD8^+^ T cells kill the MHC I-peptide complex^+^CD8^+^ T cell, as a mechanism to suppress T cell responses^3^. Should this be the process by which T cells acquire CD19 or CD20 in disease related inflammatory processes, CD20^+^CD8^+^ and CD19^+^CD8^+^ T cells, despite their pathogenic potential, might not even play any role in disease pathology, due to being marked for and eliminated by fratricide.

Our experiments also revealed the existence of CD19^+^ myeloid cells. We found that phagocytes, both of murine and human origin, acquire CD19 via efferocytosis, the clearance of apoptotic cells via phagocytosis. Especially in continuous efferocytosis, where phagocytes continually ingest apoptotic cells, membrane recycling from apoptotic cells into the plasma membrane of the phagocyte has been established^15,20^. In efferocytosis, membrane recycling is an essential process for phagocytes to remain able to continually reform their membrane to surround and enclose apoptotic cells. Apparently, in this process, also membrane-bound molecules are recycled, and this transfer is even possible across species, underlining the robustness of this process. The transfer of CD19 via efferocytosis was implicated by the strong correlation between the amount of CD19 on the monocyte surface and the expression of CD40 and MHC II, markers associated with phagocytosis. This indirectly proves ex vivo what we could show via phagoassays with apoptotic B cells in vitro. CD19 thus marks phagocytes for their recent ingestion of apoptotic B cells. In addition, CD19^+^ phagocytes proved to also be positive for other B cell antigens, such as IgM and IgD, which were shown to be functional there. Thus, CD19^+^ phagocytes gain B cell-properties and are accessible to anti-CD19 depletion.

Whether depletion of CD19^+^ phagocytes may have any impact on the therapeutic potential of inebilizumab remains unclear and may depend on the circumstances of its use. What needs to be considered though is that the process in which CD19 is acquired by phagocytes is of great importance for myeloid cell development and differentiation. In this regard, it has been described that phagocytes under continuous efferocytosis change their expression profile to a regulatory one with increased expression of the anti-inflammatory cytokine IL-10^15,21^. In addition, a feedback loop between those phagocytes and regulatory T cells as a means to suppress the immune response has been depicted^22^. Of note, to potentially deplete these regulatory CD19^+^ phagocytes generated by efferocytosis would concomitantly eliminate this mechanism of immunoregulation. In the bigger picture, it remains elusive whether CD19^+^ phagocyte depletion is beneficial, detrimental, or a mix of both and clearly, further research is required to answer this question.

Taken together, our research revealed that various forms of antigen transfer continuously happen in cell-cell interaction and that these subcellular processes have an enormous impact both for physiological homeostasis, as well as for many pathophysiological cascades underlying disease development and propagation. Furthermore, they tremendously alter our view and conception of many antigen-specific therapeutic approaches depending on the cellular specificity of lineage markers. Nowadays, where monoclonal antibody and CAR T cell therapies are widely-used and continue to grow as treatment options, the impact of antigen transfer between cells intentionally targeted and concomitantly addressed, such as tumor cells and tumor-fighting immune cells, needs to be considered and implemented into advanced treatment regimens. Besides these important therapeutic implications, antigen transfer between cells may also offer an opportunity to decipher currently unclear pathological processes via molecular tracing, as transferred antigens offer a short-term molecular memory of cellular interactions. In context with these considerations, our findings reported here highlight that we have just begun to understand and explore the potential of intercellular molecule transfer and that further research in this field should provide important advances.

## Methods

### Human samples

PBMCs were obtained after informed consent. The protocol was approved by the Ethics committee of the University Medicine of Göttingen (3/4/14). For longitudinal analysis, only relapse-free patients were included. PBMCs kindly provided by the university center of Berlin, Charite fall under the EA number EA1/362/20.

### Mice

Wild type (wt) C57BL/6 mice were purchased from Charles River (Strain Code: 027). MOG_p35-55_ TCR transgenic 2D2 mice were kindly provided by Dr. Kuchroo (Boston, USA, C57BL/6-Tg(Tcra2D2,Tcrb2D2)1Kuch/J; also available from Jackson Laboratory, strain #006912). CD19-cre mice were kindly provided by the AG Lalive (B6.129P2(C)-Cd19tm1(cre)Cgn/J; strain #006785). µMT mice (B6.129S2-Ighmtm1Cgn/J; strain #:002288), MHC IIKO mice (B6.129S2-H2dlAb1-Ea/J; strain #003584), and OVA_329-337_ TCR transgenic OTII mice (B6.Cg-Tg(TcraTcrb)425Cbn/J; strain #004194) were purchased from Jackson Laboratory. µMT mice, MHC IIKO mice, CD19-cre mice, and OT II mice were bred and held under barrier conditions until they were transferred into specific pathogen-free conditions for the experiments. 2D2 mice, C57BL/6 mice, and all mice during the experiments were kept under specific pathogen-free conditions. Experimental and control animals of the same strain were cohoused. Animals of different strains were kept in separate cages and bred separately. All mice were held with a 12:12 h dark:light cycle at a room temperature of 20–24 °C with a humidity of 45–65%. For organ extraction, mice were euthanized via deep CO_2_ narcosis followed by cervical dislocation. Apart from the EAE-induced C57BL/6 mice from Fig.3a–d and Fig.4, which were exclusively female, both genders were used as available. The age of the mice used for the experiments was generally 7–36 weeks, with the exception of Fig.1g,h where also 1, 2, and 3 week-old mice were used. All animal experiments were carried out in accordance with the Central Department for Animal Experiments, University Medical Center, Göttingen and approved by the Office for Consumer Protection and Food Safety of the State of Lower Saxony (protocol number 33.9-42502-04-15/1804, 33.9-42502-04-16/2267, and 33.9-42502-04-21/3680, 33.9-42502-04-20/3489).

### Isolation of human and murine leukocytes

PBMCs were isolated after Biocoll gradient centrifugation. Single-cell suspensions of murine lymphoid tissues were generated and passed through a 70 µm cell strainer. Murine blood was collected in PBS containing 1 mM EDTA and erythrocytes were lysed using BD Pharm Lysing Buffer (#555899). Murine splenic and human blood B cells were purified or removed by MACS separation (MojoSort Mouse CD19 Nanobeads, #480002; MojoSort Human CD19 Selection Kit, #480106, BioLegend). Murine and human T cells were isolated by negative MACS separation using the mouse pan T cell isolation kit II (Miltenyi; #130-095-130) or the MojoSort Human CD3 T Cell Isolation Kit (BioLegend; #480131).

### Anti-CD3 / anti-CD28 stimulation

For the analysis of T cell proliferation, T cells were stained with carboxyfluorescein succinimidyl ester (CFSE, BioLegend; #423801), while T cells remained unstained for the evaluation of differentiation. T cells were incubated in anti-CD3 (clone 145-2C11 for murine T cells; #100359)/anti-CD28 (clone 37.51 for murine T cells; #102121) (BioLegend) pre-coated wells for 48–72 h.

### Anti-IgM/G stimulation

For the functional analysis of B cell marker on T cells and CD11b^+^ cells, cells were stained for FACS-sorting, sorted, and incubated (117,000 T cells or 27,500 CD11b^+^ cells per well) with different concentrations of AffiniPure F(ab’)2 Fragment Goat Anti-Mouse IgG + IgM (H + L) (Jackson ImmunoResearch Europe Ltd.; RRID: AB_2338471) in 96-well plates overnight.

### T cell-B cell-coculture assays

For murine B cell–T cell coculture assays, B cells from wild-type, MHC IIKO, or CD19-cre mice and T cells from MOG_35-55_ TCR transgenic 2D2 mice were MACS-separated from splenocytes. For human B cell–T cell coculture assays, B cells and T cells were isolated via MACS from PBMCs from healthy donors. Following separation, B cells and T cells were evaluated for purity (>98%) by FACS staining for CD19 and CD3. 400,000 B cells and 50,000 T cells were plated in 96-well plates for culture and after 24–48 h, T cells were evaluated by flow cytometry. Murine cocultures were stimulated with MOG_35-55_ peptide, humane cocultures were stimulated with Staphylococcus enterotoxin B (SEB, Sigma-Aldrich Israel Ltd.; #S4881-1MG). To control for effects independent of cell-cell contact, a coculture was performed in transwell plates, where B cells and T cells were separated by an impenetrable 0.4 µm membrane. Additionally, B cells were stained with membrane stain (MemBrite Fix 488/515; Biotium; #30093) according to the manufacturer’s instructions.

### BMDM generation

BMDMs were generated by isolating the bone marrow from 1–2 femurs of C57Bl/6 J or CD19-cre mice and stimulating it with L929 medium [DMEM (Pan Biotech; # P04-03590), 30% L929 cell-conditioned medium, 10% fetal calf serum (Anprotec; #AC-SM-0190), 5% horse serum (Sigma-Aldrich; #H1138), 50 U/ml penicillin + 50 μg/ml streptomycin (Life Technologies; #15070-063), 0.05 mM β-mercaptoethanol (Gibco; #31350-010)] at 37 °C and 5 % CO_2_ for seven days. Adherent BMDMs were harvested using cell scrapers. Cultures contained >95% myeloid cells verified by flow cytometry.

### Microglia generation

For the generation of primary microglia, brain cells of new-born to two-day-old C57BL/6 J mice were enzymatically isolated with 0.4 mg DNAse I (Roche; #05952077103) and 2.5% trypsin (Pan Biotech; #P10-022100). A mixed glial cell culture was achieved by cultivating the cells in DMEM containing 10% fetal calf serum, 1% GlutaMax (Thermo Fisher Scientific; # 35050061), 100 U/ml penicillin, and 100 µg/ml streptomycin at 37 °C and 5% CO_2_ until confluency. Thereafter, cells were stimulated with a medium containing DMEM, 30% L929 cell-conditioned medium, 10% fetal calf serum, 100 U/ml penicillin, and 100 µg/ml streptomycin for five days, to gain an enriched microglia culture. To separate primary microglia from other glia cells, microglia were harvested by gentle shaking at 90 rpm for 30 min at 37 °C. The generated cultures contained >97% microglial cells verified by flow cytometry.

### Human monocyte-derived macrophages generation

Human monocyte-derived macrophages (hMDM) were generated by isolating PBMCs as mentioned above. Monocytes were isolated by positive selection using CD14 MicroBeads (Miltenyi; #130-050-201) according to manufacturer´s instruction. Monocytes were cultured at 37 °C, 5% CO_2_ for 5 days in medium [AIM V medium (Thermo Fisher Scientific; #12055091), 10% human serum (Bio&Sell; #HUAB.SE.0100), 50 U/ml penicillin, 50 µg/ml streptomycin)], containing 50 ng/ml human M-CSF (Miltenyi Biotech; # 130-096-491), followed by 2 days in medium (AIM V medium, 10% human serum, 50 U/ml penicillin, 50 µg/ml streptomycin), containing 10 ng/ml human IFN-γ (BioLegend; #570206).

### Human iPSC-derived microglia generation

Human induced pluripotent stem cells (hiPSC) UMGi130-A clone 8 (isWT11.8) maintained in StemMACS iPSC-Brew XF (Brew; Miltenyi; #130-104-368) were differentiated into hiPSC-derived hematopoietic progenitors (iHPCs) and hiPSC-derived macrophages as previously described^23^ with some modifications followed by differentiation into hiPSC-derived microglia (iMG). For the hematopoietic differentiation hiPSCs were dissociated using Accutase (Gibco, Thermo Fischer; #A1110501) to generate a single-cell suspension. 60,000 cells/cm² were plated in Brew containing 7.5 ng/ml Activin A (Proteintech; #HZ-1138), 30 ng/ml BMP4 (Proteintech; #HZ-1045), 3 µM CHIR 99021 (Selleckchem; #S1263), and 10 µM ROCK inhibitor (Y-27632; Selleckchem; #S1049) onto Matrigel (Corning)-coated plates. After 18 h, medium was changed to TeSR-E6 (E6; Stemcell; #05946) containing 10 ng/ml Activin A, 40 ng/ml BMP4, and 20 µM IWP2 (Selleckchem; #S7085). On day 2, medium was changed to E6-containing 10 ng/ml Activin A, 40 ng/ml BMP4, 20 µM IWP2, and 20 ng/ml FGF-basic (Peprotech; #AF-100-18B). On day 3, the cells were dissociated by Accutase and replated at 30,000 cells/cm² onto Matrigel-coated plates in E6-containing 15 ng/ml VEGF 165 (Peprotech; #AF-100-20), 5 ng/ml FGF-basic, and 10 µM ROCK inhibitor (Selleckchem; #Y-27632). On day 4, medium was changed to E6-containing 15 ng/ml VEGF 165 and 5 ng/ml FGF-basic. On days 5 and 6, medium was changed to E6-containing 15 ng/ml VEGF 165 and 5 ng/ml FGF-basic, 200 ng/ml SCF (Proteintech; #HZ-1024), and 20 ng/ml IL-6 (Proteintech; #HZ-1019). On day 7, medium was fully replaced and on day 8, a half medium change was performed with E6 containing 100 ng/ml SCF, 10 ng/ml IL-6, 30 ng/ml TPO (Proteintech; #HZ-1248-GMP), and 30 ng/ml IL-3 (Thermo Fisher Scientific; #200-03), which was used for all further medium changes of the hematopoietic differentiation. On day 9, the supernatant containing iHPCs in semi-suspension was collected and centrifuged at 300 g for 8 min. To the remaining hematopoietic differentiation 50% cell-free conditioned medium and 50% fresh medium were added. The collected iHPC pellet was plated at 10,500 cells/cm² onto Matrigel-coated plates in iMG medium [75% IMDM (Gibco, Thermo Fisher Scientific; #21980032), 25% F12 (Gibco, Thermo Fisher Scientific; #21765029) medium, 1x B-27 supplement (Gibco, Thermo Fisher Scientific; #17504044), 1x GlutaMAX supplement (Gibco, Thermo Fisher Scientific; #35050061), 100 ng/ml IL-34 (Proteintech; #HZ-1316-GMP), and 20 ng/ml M-CSF (Proteintech; #HZ-1192-GMP)]. Further harvests of iHPCs from the remaining hematopoietic differentiation were similarly performed on day 11 and 14, with half of the volume of medium being exchanged on day 13. New iMG medium was supplemented every second day. On day 21, the cells were mechanically dissociated and replated at 10,500–20,800 cells/cm² onto Matrigel-coated plates in iMG medium. New iMG medium was supplemented every second day. On day 28, the cells were mechanically dissociated and replated at 20,800–52,000 cells/cm² onto Matrigel-coated plates in iMG medium. Half medium changes were performed every second day until final analysis. From day 35 the cells were considered hiPSC-derived microglia and their culture was continued for up to 30 days. The hiPSC line was kindly provided by Dr. Lukas Cyganek.

### Phagoassays

Mature BMDMs or microglia were harvested by scraping with cell scrapers and plated at a concentration of 250,000 BMDMs or 300,000 microglia in 500 µl per well in 24 well plates. A portion of the BMDMs were prestimulated with 500 ng LPS (Sigma, #L4391-MG), while a portion of microglia were prestimulated with 10 ng/ml IFNγ (BioLegend; #570206) overnight. Thereafter, LPS/IFNγ was removed and the cells were washed once with PBS. A portion of the cells were then prestimulated with 100–200 ng Latrunculin A (Sigma-Aldrich; #L5163-100) for 30 min at 37 °C. After the prestimulation, either 0.5–1 × 10^6^ apoptotic B cells or 0.5–1 µg/ml FITC-labeled Ovalbumin (Thermo Fisher Scientific; #O23020) were added and incubated for 2.5 h at 37 °C. To generate murine apoptotic B cells, B cells were isolated from wildtype mouse spleens 2-4 days before the assay and incubated without stimulation at 37 °C and 5% CO_2_. After 2–3 days 50–75% of the B cells were either dying or dead, confirmed by flow cytometry. Apoptotic B cells and their particles can be phagocytosed in a process called efferocytosis. After the 2.5 h timeframe for phagocytosis, B cells/OVA were removed and the phagocytes were washed twice with PBS, before they were scraped with cell scrapers and plated in 96-well plates for staining. Microglia were incubated with Trypsin/DNase for 4 min at 37 °C before scraping.

Differentiated hMDMs were harvested using a cell scraper. 50,000 cells/well were plated into 96-well round bottom plates and rested for 30 min at 37 °C. For the generation of human apoptotic B cells, PBMCs were generated from healthy donors, and B cells were isolated using the B Cell Isolation Kit II (Miltenyi; #130-091-151), according to manufacturer´s instructions. B cells were incubated without stimulation for 3 days at 37 °C, 5% CO_2_. 250,000 apoptotic B cells were added to the hMDMs and incubated together for 2.5 h at 37 °C, 5% CO_2_. Afterwards, the B cell-containing supernatant was removed and hMDMs were washed using PBS before flow cytometry staining.

Differentiated hiPSC-derived microglia were harvested using cold PBS. 20,000 cells/well were plated into poly-L-lysin (Merck; # P1274) coated 96-well flat bottom plates and incubated overnight at 37 °C. Apoptotic murine B cells were generated as described above. 50,000, 100,000, or 200,000 apoptotic B cells were added to the hiPSC-derived microglia and incubated together for 2 h at 37 °C, 5% CO_2_. Afterwards, hiPSC-derived microglia and murine B cells were detached using cold PBS and washed using PBS before flow cytometry staining.

### EAE induction and scoring

Female C57BL/6 J mice were actively immunized subcutaneously with either 100 µg MOG_35-55_ peptide MEVGWYRSPFSRVVHLYRNGK (Auspep) or 75 µg MOG_1-117_ protein (GenScript Biotech) emulsified in Complete Freund’s Adjuvant (CFA; Sigma-Aldrich; #F5881) containing 250 µg killed *Mycobacterium tuberculosis* H37 Ra (BD Bioscience; #231141) followed by intraperitoneal injections of 200 ng of *Bordetella pertussis* toxin (Sigma-Aldrich; #516560) on the day of immunization and 2 days thereafter. EAE severity was assessed daily and scored on a scale from 0 to 5 as follows: 0 = no clinical signs; 1.0 = tail paralysis; 2.0 = hindlimb paresis; 3.0 = severe hindlimb paresis; 4.0 = paralysis of both hindlimbs; 4.5 = hindlimb paralysis and beginning forelimb paresis 5.0 = moribund/death.

### Flow cytometry

Murine immune cells were analyzed using the following antibodies in a 1:100 dilution if not otherwise declared: CD3-PE, FITC, BV605, or BUV395 (145-2C11; BioLegend; #100308, #100306, #100351; BD Biosciences; #563565), CD4-BV510 or FITC (GK1.5; BioLegend; #100449, #100406), CD8-BV421, FITC or PerCP-Cy5.5 (53-6.7; BioLegend; #100738, #100706, #100734), CD45R/B220-PE-CY7 (RA3-6B2; BioLegend; #103222), CD11b-BV510, PE or PE-Cy7 (M1/70; BioLegend; #101263, #101208; BD Biosciences; #552850), CD11c-PE/Dazzle (N418; BioLegend; #117348), CD19-PerCP-Cy5.5, PE, or AF647 (1D3; BioLegend; #152406, #152408; BD Biosciences; #557684), CD20-AF647 (SA275A11; 1:1000; BioLegend; #150404), IgD-BV421 (11-26 c.2a; BioLegend; #405725), IgM-BUV395 (AF6-78, BD Biosciences; #742349), Ly6C-BV421 (HK1.4; BioLegend; #128032), Ly6G-BV785 (1A8; BioLegend; #127645), MHC II-BV421 or BV785 (M5/114.15.2, BioLegend; #107631, #107645), and NK1.1-BV605 (PK136; BioLegend; #108740). CD19^+^ T cells, BMDMs, NK cells, microglia, and monocytes were determined using pre-gates for single cells, cell size, living cells, and CD20^-^ cells and gated on the equivalent CD19-cre cells or isotype controls/fluorescence minus ones as negative gating controls. T cell and monocyte activation was investigated using: CD11a-FITC (M17/4; BioLegend; #101106), CD25-PE or BV421 (PC61.5; BioLegend; #102008, #102033), CD40-PE-CF594 (3/23, BD Biosciences; #562847), CD49d-PE (9C10; BioLegend; #103705), and CD69-PE-Cy7 or BV711 (H1.2F3; BioLegend; #104512, #104537), CD80-APC (16-10A1, BioLegend; #104714), CD86-BV421 (GL-1, BioLegend; #105032), and CD154-APC (MR1, BioLegend; #106510). Fc receptors were blocked using monoclonal antibody specific for CD16/CD32 (93; BioLegend; #156604).

CD3-BV510 or BV711 (UCHT1; BioLegend; #300448, #317328), CD4-PE-Cy7 or BV605 (RPA-T4; BioLegend; #300512, #300556), CD8-FITC or PerCP-Cy5.5 (RPA-T8; BD Bioscience; #561948, #560662), CD11c-PE/Cy5.5 (3.9, eBioscience; #35-0116-42), CD14-PerCP-Cy5.5 or BV421 (M5E3; BioLegend; #301824; BD Biosciences; #565283), CD14-PE/CF594 (MΦP9, BD Biosciences; #562335), CD16-PE-Cy7 (3G8; BioLegend; #302016), CD19-AF647 (SJ25C1; BioLegend; #363040), CD20-PE (REA780; Miltenyi Biotec; #130-111-338) were used for the surface staining of human PBMCs. Human T cells were analysed for their phenotype, developmental stage, and activation using CCR4-BV510 (1G1; BD Biosciences; #563066), CCR6-BV605 (11A9; BD Biosciences; #562724), CCR7-PE-CF594 (150503; BD Biosciences; #562381), CD45RO-AF700 (UCHL1; BD Biosciences; #561136), CXCR3-BV786 (1C6; BD Biosciences; #353738), CD49d-BV421 (9F10; BioLegend; #304322) and CD69-BV785 (FN50; BioLegend; #310932). To investigate the cytokine profile of human T cells, PBMCs were stimulated with 10 ng/ml PMA (Sigma-Aldrich; #P8139) and 0.5 µg/ml ionomycin (Sigma-Aldrich; #AABH9A95673F) first for 1 h, followed by 12 h in the presence of 1 µl/200 µl brefeldin A (BD Bioscience; #15847968). To assess human T cell cytokine production IL-17-PE-Cy7 (BL168; 1:50; BioLegend; #512315), IFN-γ-BV421 (B27; 1:50; BioLegend; #506538), GM-CSF-PE/Dazzle (BVD2-21C11; 1:50; BioLegend; #502318), and TNF-AF700 (Mab11; 1:50; BD Biosciences; #557996) were utilized after fixation and permeabilization (BD Bioscience; #554714). To assess human monocyte activation CD40-PE/Dazzle (5C3; BioLegend; #334342), CD80-PE-Cy7 (L307.4; BD Biosciences; #561135), CD86-BV605 (GL-1; BioLegend; #105037), CD163-FITC (GHI/61; BioLegend; 333618), CCR2-BV421 (K036C2; BioLegend; #357210), CCR5-PerCP-Cy5.5 (J418F1; BioLegend; #359111), and CX3CR1-PE (K0124E1; BioLegend; #355704) were stained. Isotype control antibodies were applied as negative staining control: Alexa Fluor 647 mouse IgG1,k isotype ctrl (MOPC-21; BD Biosciences; #557714), Alexa Fluor 700 mouse IgG1,k isotype ctrl (MOPC-21; 1:50; BD Biosciences; #557882), BV421 mouse IgG1,k isotype ctrl (MOPC-21; 1:50; BioLegend; #400158), BV510 mouse IgG1,k isotype ctrl (X40; BD Biosciences; #562946), BV605 mouse IgG1,k isotype ctrl (X40; BD Biosciences; #562652), BV785 mouse IgG1,k isotype ctrl (MOPC-21; BioLegend; #400170), PE-CF594 mouse IgG2a,k isotype ctrl (G155-178; BD Biosciences; #562306), PE-Cy7 mouse IgG1,k isotype ctrl (MOPC-21; 1:50; BD Biosciences; #557872), REA control (S) antibody (REA293; 1:1000; Miltenyi Biotec; #130-113-438). Fc receptors were blocked using a monoclonal antibody specific for CD16/CD32 (Human TruStain FcX (Fc Receptor Blocking Solution); BioLegend; #422302). Dead human and murine cells were stained with LIVE/DEAD Fixable NIR Dead Cell Stain Kit (Thermo Fisher Scientific; 1:500; #423106). Samples were acquired on a BD LSRII cytometer (BD Bioscience). All data evaluation was performed using FlowJo software (FlowJo LLC).

### Fluorescence microscopy

For fluorescence microscopy B cells were stained with a membrane stain (MemBrite Fix 488/515 or 640/660; #30093 or #30097) according to the manufacturer´s instructions before the coculture. After B cell-T cell coculture, the cells were stained with CD3-FITC (145-2C11; BioLegend; #100306) and CD19-PE (1D3; BioLegend; #152408) for 1 h at room temperature and directly analyzed on cover slips. BMDMs were seeded on coverslips for microscopic analysis. After the phagoassay, BMDMs were stained with CD11b-PE (M1/70; BioLegend; #101208) and CD19-AF647 (1D3; BD Biosciences; #557684) for 1 h at room temperature. For the analysis, a digital camera (DP71; Olympus Europa GmbH, Hamburg, Germany) mounted on a fluorescence microscope (BX51; Olympus Europa GmbH) was used.

### Enzyme-linked immunosorbent assay

Production of IFN-γ, IL-17, IL-4, TNF, and GM-CSF was measured using ELISA MAX Standard Set kits (BioLegend; #430801, #432501, #431101, #430901, #432201). IL-2 and IL-10 production was measured employing ELISA MAX Standard Set kits (R&D Systems; #DY402, #DY417). Absorbance was measured at 450 nm with subtraction of a 540 nm reference wavelength on iMark microplate reader (Bio-Rad laboratories Inc.).

### RNA-Seq and bioinformatical analysis

B cells, T cells, classical monocytes, and NK/NKT cells were isolated from PBMCs of healthy subjects (Supplementary data table 1) and B cells, T cells, and CD11b^+^ cells from wild-type mice. Total RNA was isolated with the RNeasy Plus Micro Kit according to the manufacturer’s protocol (Qiagen; # 74134). RNA was quantified with a Qubit 2.0 fluorometer (Invitrogen) and the quality was assessed on a Bioanalyzer 2100 (Agilent) using an RNA 6000 Pico chip (Agilent; #5067-1513). Samples with an RNA integrity number (RIN) of > 8 were used for library preparation. Barcoded mRNA-seq cDNA libraries were prepared from 70 ng of total RNA using NEBNext Poly(A) mRNA Magnetic Isolation Module and NEBNext Ultra II RNA Library Prep Kit (New England Biolabs; # E7490S, # E7770L) for Illumina according to the manual with a final amplification of 13 PCR cycles. Quantity was assessed using Invitrogen’s Qubit HS assay kit (#Q33230) and library size was determined using Agilent’s 2100 Bioanalyzer HS DNA assay (#5067-4626, # 5067-4627). Barcoded RNA-Seq libraries were sequenced at Novogene (Cambridge, UK) on an Illumina NovaSeq Cycle). The raw output data of the NovaSeq was preprocessed according to the Illumina standard protocol. Sequence reads were trimmed for adapter sequences and further processed using Qiagen’s software CLC Genomics Workbench (v20.0 with CLC’s default settings for RNA-Seq analysis). Reads were aligned to GRCh38 genome.

### Statistical analysis

Statistics were calculated using the software GraphPad Prism 8. Gauss distribution was tested for via D’Agostino & Pearson normality test. The respective statistical comparisons used are indicated in the figure legends. A value of *p* ≤ 0.05 was considered significant and is shown by one asterisk. Two asterisks, three asterisks, and four asterisks indicate significances of *p* ≤ 0.01, *p* ≤ 0.001 and *p* ≤ 0.0001, respectively.

### Reporting summary

Further information on research design is available in the Nature Portfolio Reporting Summary linked to this article.

## Supplementary information

Supplementary Information

Transparent Peer Review file

Reporting Summary

## Source data

Source Data file

## Data Availability

The Bulk RNA sequencing data have been deposited in Gene Expression Omnibus (GEO) under GSE286404 and GSE286403. All data are included in the Supplementary Information or available from the authors, as are unique reagents used in this Article. The raw numbers for charts and graphs are available in the Source Data file whenever possible. Source data are provided with this paper.
