# Supplementary material for: B cell CD19 is transferred between immune cells in mice and humans
Source: Nat Commun. 2026 Jul 29;17:7588. doi: 10.1038/s41467-026-75534-3 (PMC13421502; doi:10.1038/s41467-026-75534-3)
Supplement: Supplementary file 1 — Supplementary Information [file 41467_2026_75534_MOESM1_ESM.pdf]

B cell CD19 is transferred between immune cells in mice and humans.

-

Supplementary Information

Jasmin Ochs[1,2], Pia Schweineberg[2], Jacqueline Thode[3], Alica Blenkle[2], Leila Husseini[1,2], Matthias Klein[4,5], Tobias Bopp[4,5], Patrick Schindler[6,7], Friedemann Paul[7], Martin S. Weber[1,2,3]

[1]Department of Neurology, University Medical Center, Göttingen, Germany
[2]Fraunhofer Institute for Translational Medicine and Pharmacology
[3]Institute of Neuropathology, University Medical Center, Göttingen, Germany
[4]Institute of Immunology, University Medical Center Mainz, Germany
[5]Research Center for Immunotherapy (FZI), University Medical Center Mainz, Mainz, Germany
[6]Department of Neurology with experimental Neurology, Charité, Berlin, Germany
[7]Max Delbrueck Center for Molecular Medicine and Charité - Universitätsmedizin Berlin, Berlin, Germany

List of figures:

Supplementary Fig.1: Purity analysis of sorted cells and control data

Supplementary Fig.2: CD19 expression of B cells or B cells as such is not required for T cell activation and proliferation

Supplementary Fig.3: B cell and T cell analysis in various organs

Supplementary Fig.4: In the development of EAE, $CD19^+$ T cells produce higher amounts of cytokines and show enhanced expression of activation markers when compared to $CD19^-$ T cells

Supplementary Fig.5: B cell marker on T cells

Supplementary Fig.6: $CD19^+$ T cells display an activated, mature phenotype with enhanced pathogenic properties which is furthered by MS.

Supplementary Fig.7: CD19 is present on monocytes due to efferocytosis

Supplementary Fig.8: Myeloid cells can receive various B cell marker

Supplementary Table 1: PBMC samples

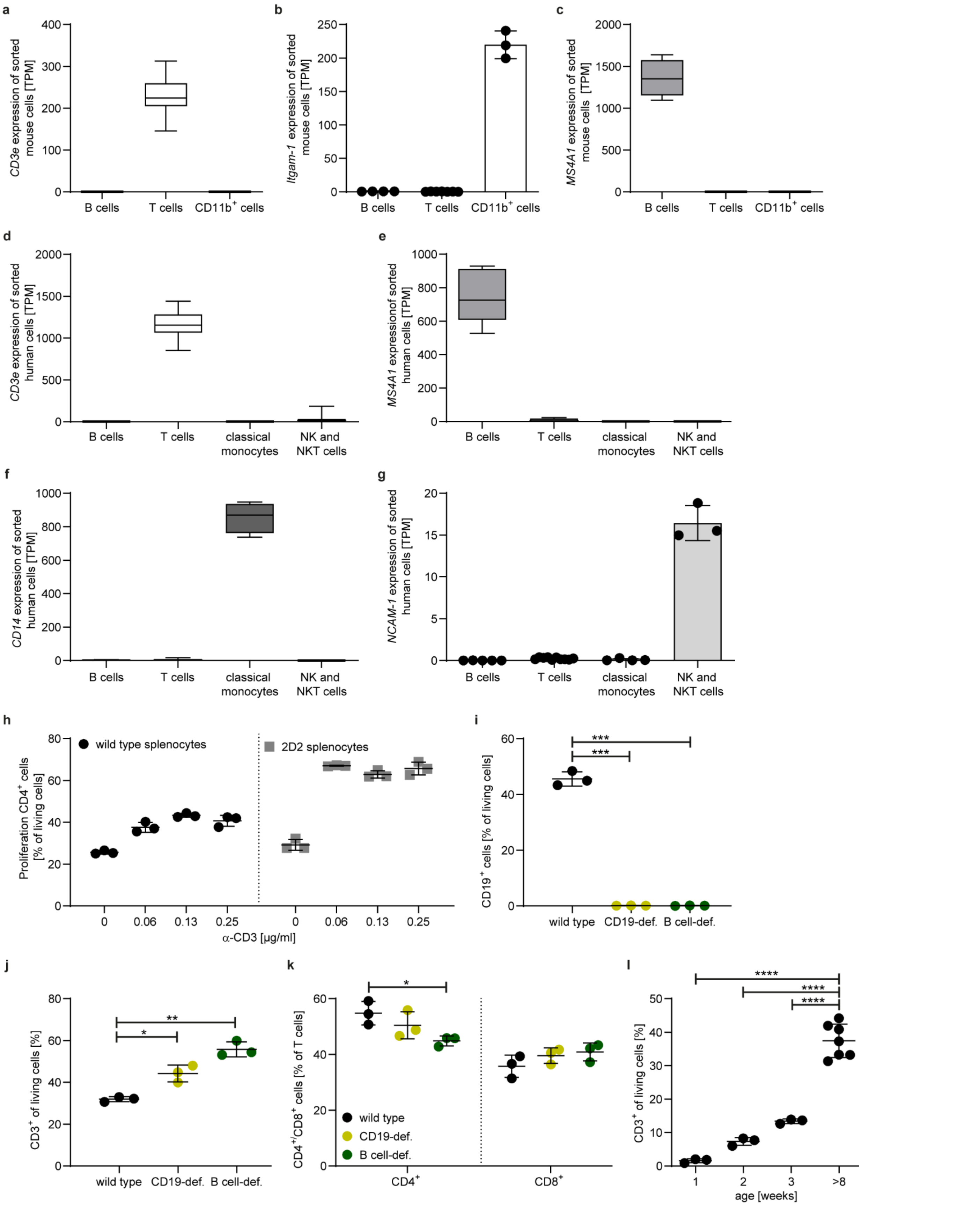

Supplementary Fig. 1: Purity analysis of sorted cells and control data. a-g Bulk RNA sequencing of fluorescence-activated cell sorted murine B cells (n = 4), murine T cells (n = 7), murine CD11b$^+$ monocytes (n = 3), human B cells (n = 5), human T cells (n = 10), human classical monocytes (n = 4), and human natural killer (NK) and natural killer T (NKT) cells (n = 3) for the transcripts **a, d** *CD3e*; **b** *Itagm-1*; **c, e** *MS4A1*; **f** *CD14*; **g** *NCAM-1*; n = mice or human peripheral blood mononuclear cell (PBMC) samples. One set of B cells, T cells, and monocytes per mouse or human; displayed as box plot/histogram. **h** Proliferation of CD4$^+$ T cells of splenocyte cultures isolated from wild type mice or myelin oligodendrocyte glycoprotein (MOG)$_{35–55}$ peptide T cell receptor (TCR) transgenic 2D2 mice and stimulated with anti-CD3/anti-CD28 antibodies for 48 hours; n = 3 wells per group. **i-k** Flow cytometric analysis of **i,** CD19$^+$ cells, **j** CD3$^+$ cells, and **k** CD4$^+$ and CD8$^+$ T cells from the spleens of wild type, CD19-deficient CD19-cre mice, and B cell-deficient µMT mice; n = 3 mice per group; analyzed via Brown-Forsythe and Welch ANOVA tests with Games-Howell's multiple comparisons test. **l** Percentage of CD3$^+$ T cells in murine spleens during development; 1/2/3 weeks: n = 3 mice per group; 8 weeks: n = 7 mice; analyzed via Brown-Forsythe and Welch ANOVA with Holm-Sidak's multiple comparisons test. All figures are representative of 2-3 independent experiments; data is displayed as means ± SD; box plots are min to max with means ± SD; *=p<0.05; **=p<0.01; ***=p<0.001; ****=p<0.0001.

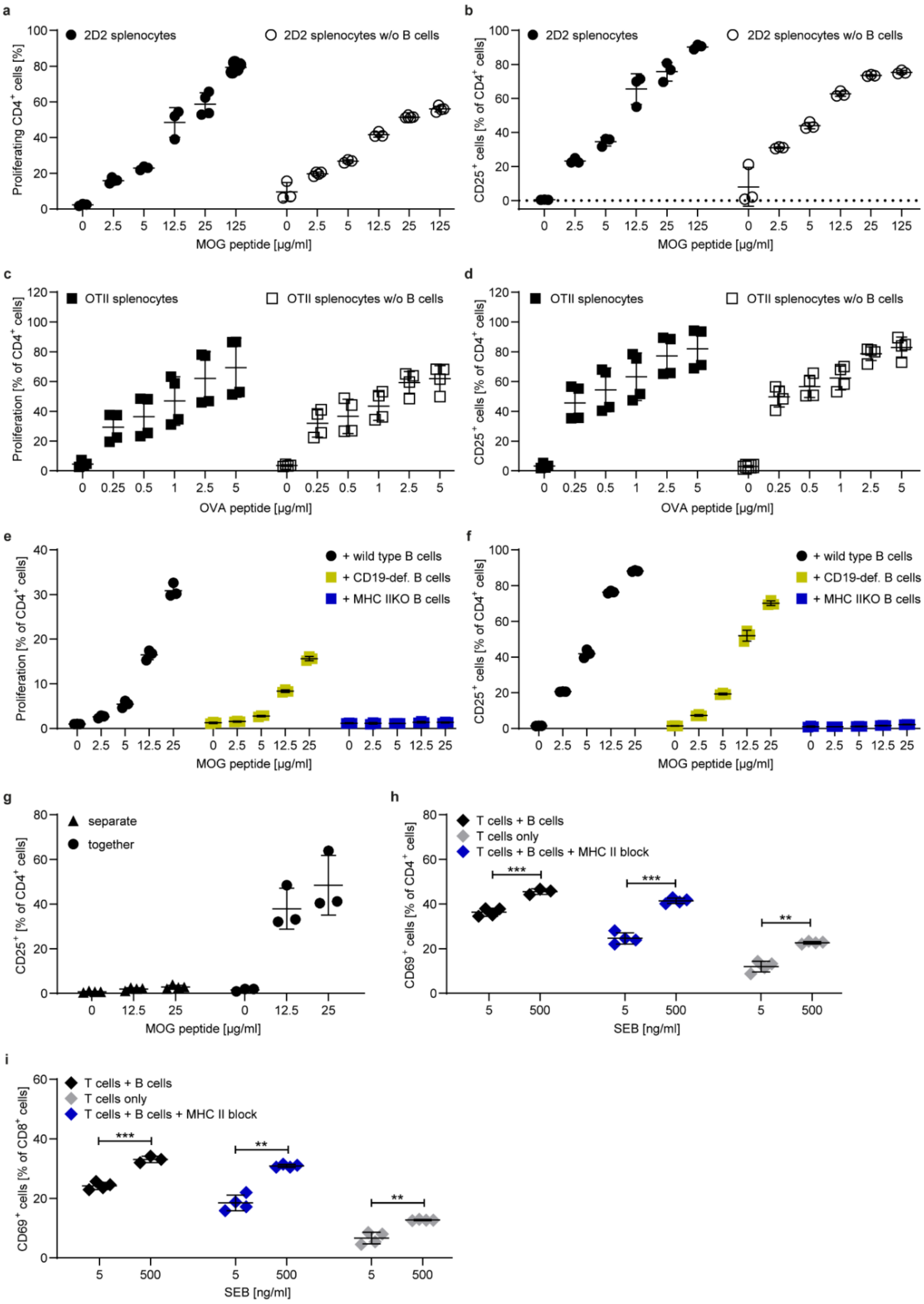

Supplementary Fig. 2: CD19 expression of B cells or B cells as such is not required for T cell activation and proliferation. a, b Splenocyte culture with and without B cells from 2D2 mice stimulated with MOG$_{35–55}$ peptide for 48 hours; **a** n = 3-4 wells per group, **b** n = 3 wells per group. **c, d** Splenocyte culture with and without B cells from ovalbumin (OVA)$_{329–337}$ peptide TCR transgenic OTII mice stimulated with OVA$_{329-337}$ peptide for 48 hours; n = 4 wells per group. **e, f** Coculture of membrane-stained (MemBrite Fix 488/515) B cells from wild type, CD19-deficient CD19-cre, or MHC IIKO mice and MOG$_{35–55}$–specific 2D2 T cells stimulated with MOG$_{35–55}$ peptide for 24 hours; n = 3 wells per group. **g** Transwell coculture and nonseparated coculture of membrane-stained wild type B cells and MOG$_{35-55}$–specific 2D2 T cells stimulated with MOG$_{35–55}$ peptide; n = 3 (together) or 4 (separate) wells per group. **h, i** Coculture of T and B cells from peripheral blood mononuclear cells (PBMC) of healthy donors together (B cells + T cells) or T cells alone (T cells only) or B cells incubated with an antibody against MHC class II (anti–HLA-DR, anti–HLA-DQ, and anti–HLA-DP) (MHC II block) 1 hour before coculture with T cells in the presence of Staphylococcal enterotoxin B (SEB) for 48 hours; analyzed via two-tailed, unpaired Student's t-test with Welch's correction; n = 4 wells per group (T cells + B cells 500 ng/ml: n= 3 wells). All cultures were analyzed via flow cytometry for their **(a, c, e)** proliferation and **(b, d, f-i)** activation (CD25, CD69) of **(a-h),** CD4$^+$ or **i,** CD8$^+$ T cells. All figures are representative of **(a, b, e-g)** or pooled from **(c, d, h, i)** at least two independent experiments; data is displayed as means ± SD; **=p<0.01; ***=p<0.001.

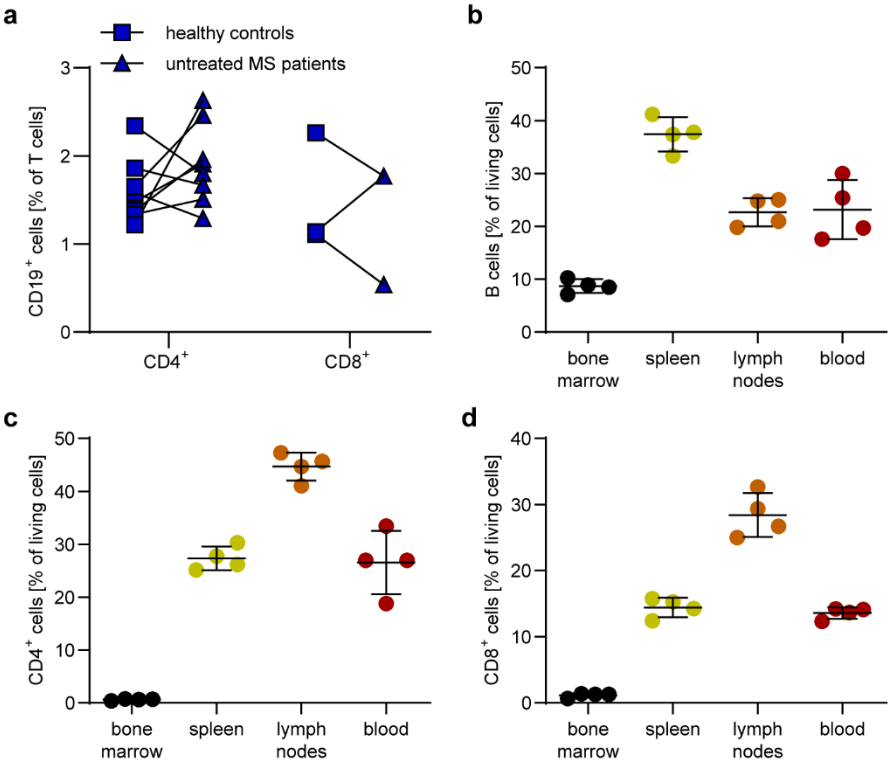

Supplementary Fig. 3: B cell and T cell analysis in various organs. a Flow cytometric analysis of the $CD19^{+}$ T cells from healthy controls vs MS patients; n = 3 ($CD8^{+}$), n = 8 ($CD4^{+}$) PBMC samples per group. **b-d** Flow cytometric analysis of B and T cells from bone marrow, spleen, inguinal lymph nodes, and blood of wild type mice; n = 4 mice per group. All figures are representative of **(a-c)** or pooled from **(d)** 2-3 independent experiments; data is displayed as means ± SD.

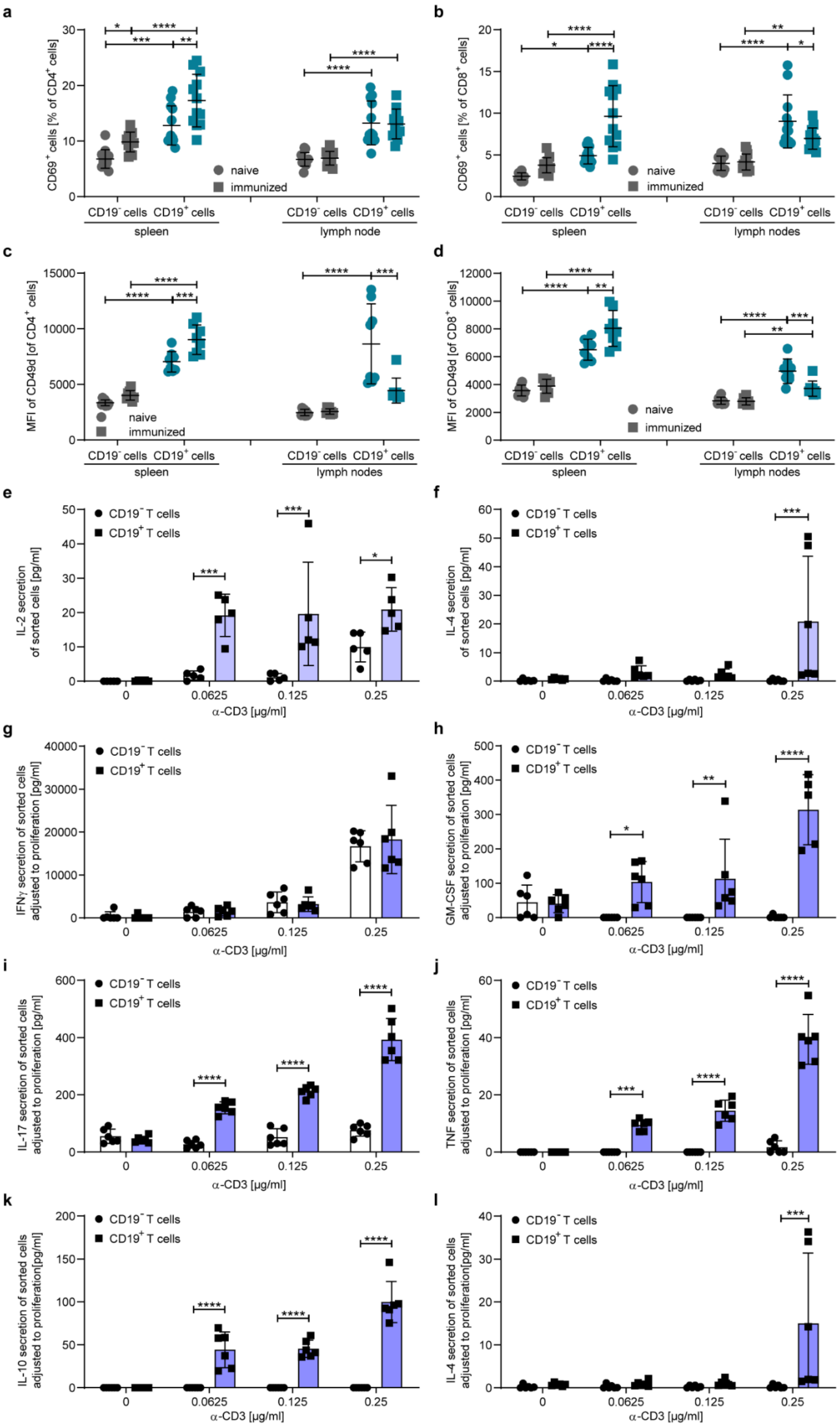

Supplementary Fig. 4: In the development of EAE, $CD19^+$ T cells produce higher amounts of cytokines and show enhanced expression of activation markers when compared to $CD19^-$ T cells. a-d Analysis of the spleen and immunization draining lymph nodes of naïve or MOG_{1-117} protein–immunized mice; n = 12 per group; analyzed via two-way ANOVA with Tukey's multiple comparisons test. Expression of **a, b** CD69 and **c, d** CD49d (mean fluorescence intensity, MFI) were evaluated on $CD19^-$ and $CD19^+$ $CD4^+$ and $CD8^+$ T cells. **e-l,** Cytokine analysis for **e** IL-2, **f** IL-4, **g** IFNγ, **h** GM-CSF, **i** IL-17, **j** TNF, **k** IL-10, and **l** IL-4 of the supernatant of $CD19^+$ and $CD19^-$ T cells from the spleens and inguinal lymph nodes of MOG_{35-55} peptide–immunized mice stimulated with anti-CD3/anti-CD28 antibodies; n = 6 wells per group, apart from **e** and **h** 0.25 $CD19^+$: n = 5; **(g-l)** data adjusted to the measured proliferation shown in **Fig. 4e**; analyzed via 2-way ANOVA with Sidak's multiple comparisons test. All figures are pooled from 2-3 independent experiments; data is displayed as means ± SD; *=p<0.05; **=p<0.01; ***=p<0.001; ****=p<0.0001.

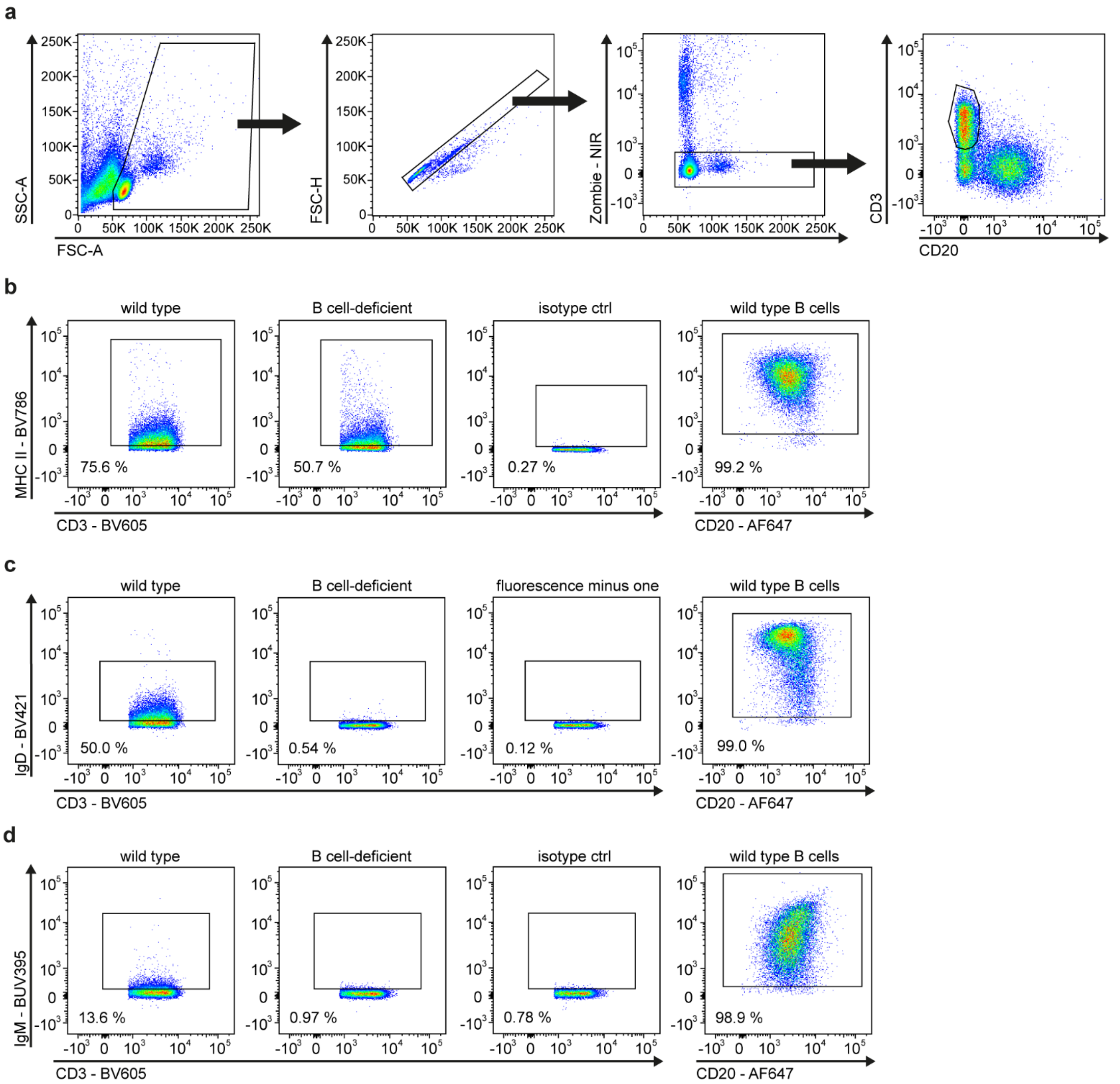

Supplementary Fig. 5: B cell marker on T cells. a Representative gating strategy for flow cytometric analysis and FACS-sorting, starting with size exclusion, followed by doublet exclusion, and the exclusion of dead cells via the life/dead staining. Thereafter T cells were gated via CD3, CD4, or CD8 against CD20 to exclude B cells, followed by gating on the marker analyzed on the T cell. **b-d** Representative flow cytometric staining of **b** MHC II, **c** IgD, and **d** IgM on $CD3^+$ T cells and $CD20^+$ B cells isolated from wild type and B cell-deficient µMt mice.

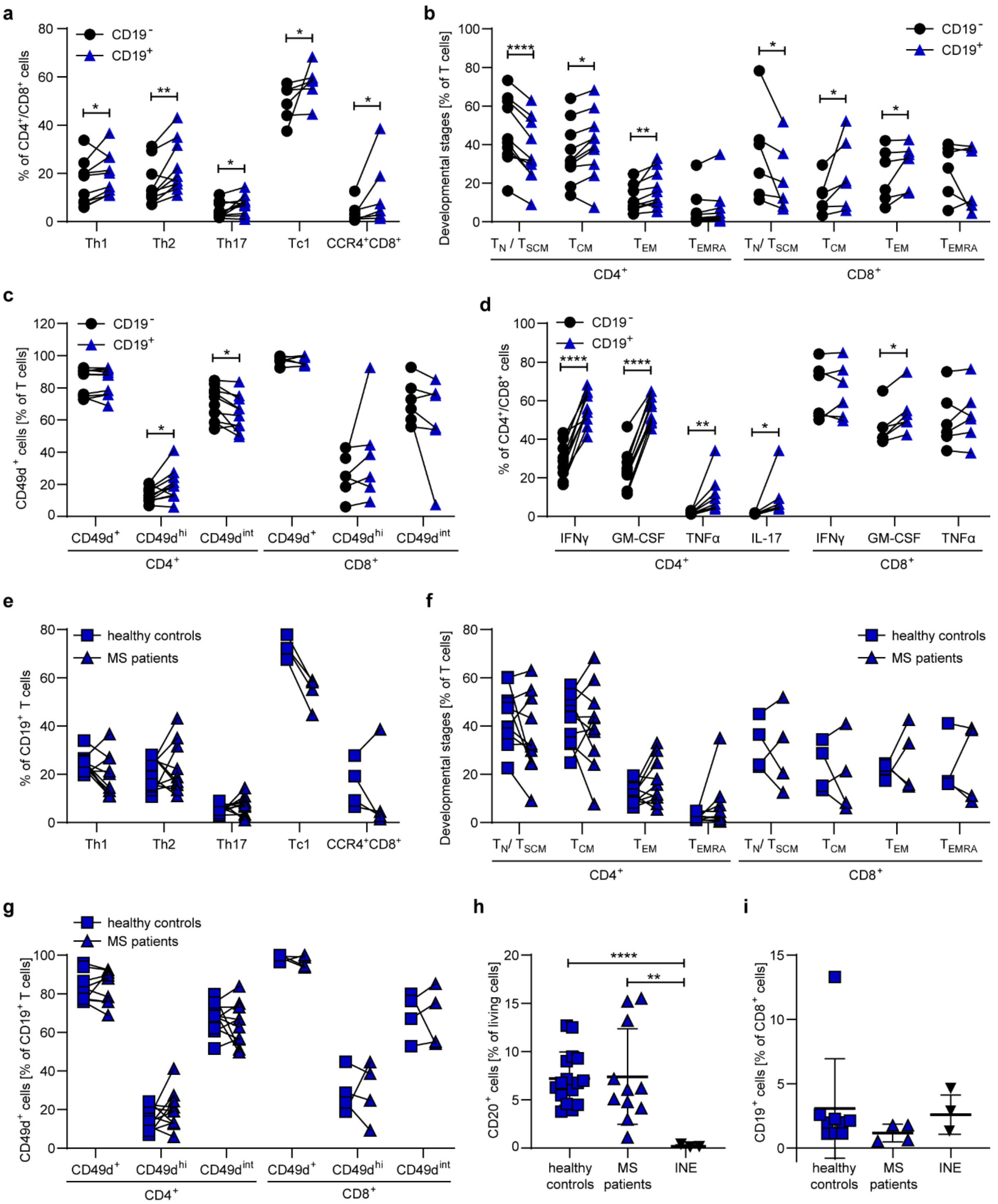

Supplementary Fig. 6: CD19$^+$ T cells display an activated, mature phenotype with enhanced pathogenic properties which is furthered by MS. a-d Flow cytometric analysis of peripheral blood mononuclear cells (PBMC) from MS patients. Comparison of CD19$^+$ versus CD19$^-$ T cells (separated into CD4$^+$ and CD8$^+$) in regard to **a** differentiation (Th1, Th2, Th17, n = 10; Tc1, CCR4$^+$CD8$^+$, n = 8), **b** developmental state [CCR7$^+$CD45RO$^-$ = T_N/T_{SCM} (naïve and stem cell–like memory), CCR7$^+$CD45RO$^+$ = T_{CM} (central memory), CCR7$^-$CD45RO$^+$ = T_{EM} (effector memory), and CCR7$^-$CD45RO$^-$ = T_{EMRA} (terminally differentiated)], **c** adhesion capability (integrin α4 = CD49d), and **d** cytokine production (IFN-γ, GM-CSF, TNF, and IL-17); analyzed by two-tailed Wilcoxon matched-pairs signed rank test. **b-d** CD4$^+$: n = 10, CD8$^+$: n = 6 PBMC samples per group. **e-g** Flow cytometric analysis of the **e** differentiation (Th1, Th2, Th17, Tc1, CCR4$^+$CD8$^+$), **f** developmental state [CCR7$^+$CD45RO$^-$ = T_N/T_{SCM} (naïve and stem cell–like memory), CCR7$^+$CD45RO$^+$ = T_{CM} (central memory), CCR7$^-$CD45RO$^+$ = T_{EM} (effector memory), and CCR7$^-$CD45RO$^-$ = T_{EMRA} (terminally differentiated)], and **g** adhesion capability (integrin α4 = CD49d) of CD19$^+$ T cells from healthy controls vs MS patients; CD4$^+$: n = 10, CD8$^+$: n = 4 PBMC samples per group; analyzed by two-tailed Wilcoxon matched-pairs signed rank test. **h** Flow cytometric analysis of CD20$^+$ B cells from PBMCs of healthy controls (n = 16), untreated RRMS (n = 11), and Inebilizumab (INE)-treated NMOSD patients (n = 4); analyzed via Brown-Forsythe and Welch ANOVA test via Games-Howell's multiple comparison test after analysis for normality via D'Agostino & Pearson test. **i,** Flow cytometric analysis of CD19$^+$ CD8$^+$ T cells from PBMCs of healthy controls (n = 9), untreated RRMS (n = 4), and INE-treated NMOSD patients (n = 4); analyzed via Kruskal-Wallis test with Dunn's multiple comparisons test after analysis for normality via D'Agostino & Pearson test. All figures are pooled from 2-4 independent experiments; data is displayed as means ± SD; *=p<0.05; **=p<0.01; ****=p<0.0001.

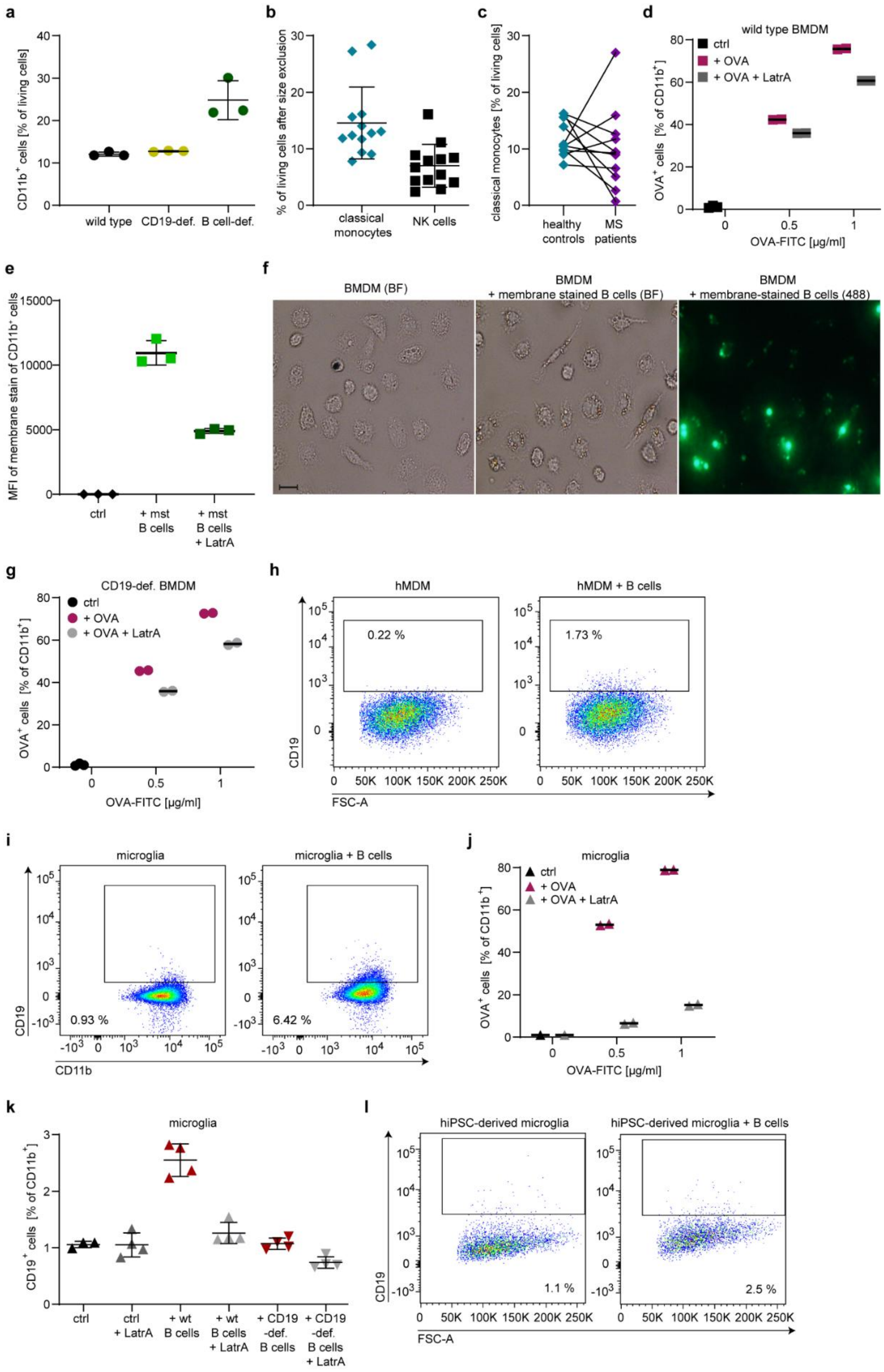

Supplementary Fig. 7: CD19 is present on monocytes due to efferocytosis. a Analysis of CD11b$^+$ monocytes isolated from the spleens of wild type, CD19-deficient, and B cell-deficient µMT mice; n = 3 mice per group. **b** Analysis of classical monocytes (CM) and NK cells of peripheral mononuclear cells (PBMC) from healthy donors; n = 13 per group. **c** Comparison of the amount of CM from PBMC of healthy donors and matched MS patients; n = 11 per group. **d, e** Analysis of the ingestion of **d** OVA-FITC or **e** apoptotic, membrane-stained (mst) B cells by wild type BMDM prestimulated with LPS and with or without Latrunculin A (LatrA) as phagocytosis inhibitor; **d** 0.5 and 1 µg/ml: n = 2 wells per group; **d** 0 µg/ml and **e**: n = 3 wells per group. **f** Representative microscopic image of BMDMs after the ingestion of apoptotic mst-B cells (MemBrite® Fix 488/515, green) in brightfield (BF) and fluorescence; scale bare measures 20 µm. **g** Analysis of the phagocytosis of Ovalbumin-FITC (OVA-FITC) by CD19-deficient CD19-cre BMDM with a prestimulation with LPS and with or without LatrA as phagocytosis inhibitor; 0.5 and 1 µg/ml: n = 2 wells 0 µg/ml 3 wells per group. **h, i** Representative staining of CD19 on **h** human monocyte derived macrophages (hMDM) or **i** CD11b$^+$ microglia with or without the ingestion of apoptotic B cells and their fragments. **j** Flow cytometric analysis of the phagocytosis of Ovalbumin-FITC (OVA-FITC) by microglia with a prestimulation with IFNγ and with or without LatrA as phagocytosis inhibitor; 0.5 and 1 µg/ml: n = 2 wells 0 µg/ml 1 well per group. **k** Analysis of CD19 on BMDM after the ingestion of apoptotic wild type or CD19-deficient B cells with a prestimulation with LPS and with or without LatrA as phagocytosis inhibitor; n = 4 (ctrl: n = 3) wells per group. **l** Representative staining of CD19 on human induced pluripotent stem cell-derived microglia with or without the ingestion of murine apoptotic B cells. All figures are representatives of **(a, d-l)** or pooled from **(b, c)** 2-3 independent experiments; displayed as means ± SD.

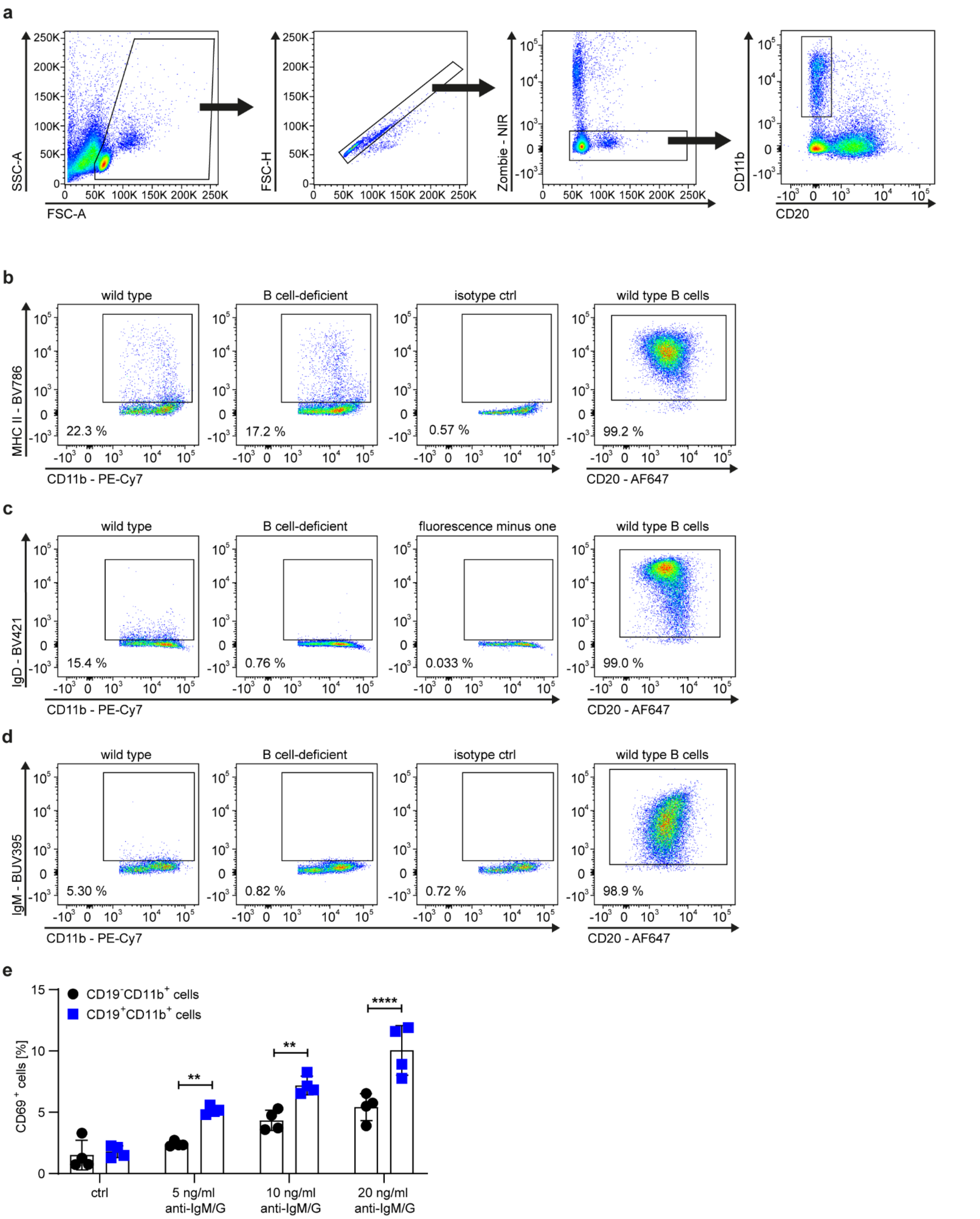

Supplementary Fig. 8: Myeloid cells can receive various B cell marker. a Representative gating strategy for flow cytometric analysis and FACS-sorting, starting with size exclusion, followed by doublet exclusion, and the exclusion of dead cells via the life/dead staining. Thereafter myeloid cells were gated via CD11b (mouse) or CD14 (human) against CD20 to exclude B cells, followed by gating on the marker of interest against CD11b/CD14. **b-d** Representative flow cytometric staining of **b** MHC II, **c** IgD, and **d** IgM on CD11b^{+} cells and CD20^{+} B cells isolated from wild type and B cell-deficient µMt mice. **e** Flow cytometric analysis of fluorescence-activated cell sorted CD19^{+} and CD19^{-} CD11b^{+} cells from the spleens of naive mice stimulated with anti-IgM/IgG F(ab′)2 fragment; n = 4 wells per group analyzed via 2-way ANOVA with Sidak's multiple comparisons test; data is displayed as means ± SD; **=p<0.01; ****=p<0.0001.

Supplementary Table 1: PBMC samples

	Healthy controls	Untreated RRMS	Inebilizumab-treated NMOSD
**Number of subjects**	15	11	3
**Sex (w/m)**	7/8	3/8	3/0
**Age at start of study (years; mean ± SD)**	42.1 ± 10.2	44.9 ± 10.5	41.7 ± 24.9
