# Supplementary material for: B cell CD19 is transferred between immune cells in mice and humans
Source: Nat Commun. 2026 Jul 29;17:7588. doi: 10.1038/s41467-026-75534-3 (PMC13421502; doi:10.1038/s41467-026-75534-3)
Supplement: Supplementary file 3 — Reporting Summary [file 41467_2026_75534_MOESM3_ESM.pdf]

nature portfolio

Corresponding author(s): Martin Weber

Last updated by author(s): 10.06.2026

Reporting Summary

Nature Portfolio wishes to improve the reproducibility of the work that we publish. This form provides structure for consistency and transparency in reporting. For further information on Nature Portfolio policies, see our Editorial Policies and the Editorial Policy Checklist.

Statistics

For all statistical analyses, confirm that the following items are present in the figure legend, table legend, main text, or Methods section.

n/a	Confirmed	
☐	☒	The exact sample size (n) for each experimental group/condition, given as a discrete number and unit of measurement
☐	☒	A statement on whether measurements were taken from distinct samples or whether the same sample was measured repeatedly
☐	☒	The statistical test(s) used AND whether they are one- or two-sided<br>*Only common tests should be described solely by name; describe more complex techniques in the Methods section.*
☐	☒	A description of all covariates tested
☐	☒	A description of any assumptions or corrections, such as tests of normality and adjustment for multiple comparisons
☐	☒	A full description of the statistical parameters including central tendency (e.g. means) or other basic estimates (e.g. regression coefficient) AND variation (e.g. standard deviation) or associated estimates of uncertainty (e.g. confidence intervals)
☐	☒	For null hypothesis testing, the test statistic (e.g. F, t, r) with confidence intervals, effect sizes, degrees of freedom and P value noted<br>*Give P values as exact values whenever suitable.*
☒	☐	For Bayesian analysis, information on the choice of priors and Markov chain Monte Carlo settings
☒	☐	For hierarchical and complex designs, identification of the appropriate level for tests and full reporting of outcomes
☐	☒	Estimates of effect sizes (e.g. Cohen's d, Pearson's r), indicating how they were calculated

Our web collection on statistics for biologists contains articles on many of the points above.

Software and code

Policy information about availability of computer code

Data collection	D™ LSRII cytometer, digital camera (DP71; Olympus 732 Europa GmbH, Hamburg, Germany) mounted on a fluorescence microscope (BX51; Olympus Europa 733 GmbH), iMark microplate reader (Bio-Rad laboratories Inc.), NEBNext® Poly(A) mRNA Magnetic Isolation Module and NEBNext® Ultra™ II RNA Library Prep 749 Kit for Illumina®, Agilent's 2100 Bioanalyzer, Illumina NovaSeq Cycle
Data analysis	GraphPad Prism 8 was used for graph preparation and statistical analysis, FlowJo software was used for data visibility and analysis of flw cytometric data, imageJ was used to analyze and visualize the microscopy data, Qiagen's software CLC Genomics Workbench was used to analyze the PCR and bulk sequencing data. Bulk sequencing data is available in GEO.

For manuscripts utilizing custom algorithms or software that are central to the research but not yet described in published literature, software must be made available to editors and reviewers. We strongly encourage code deposition in a community repository (e.g. GitHub). See the Nature Portfolio guidelines for submitting code & software for further information.

Data

Policy information about availability of data

All manuscripts must include a data availability statement. This statement should provide the following information, where applicable:

- Accession codes, unique identifiers, or web links for publicly available datasets
- A description of any restrictions on data availability
- For clinical datasets or third party data, please ensure that the statement adheres to our policy

All data are included in the Supplementary Information or available from the authors, as are unique reagents used in this Article. The raw numbers for charts and

graphs are available in the Source Data file whenever possible. The Bulk RNA sequencing data have been deposited in Gene Expression Omnibus (GEO) under GSE286404 and GSE286403 (https://www.ncbi.nlm.nih.gov/geo/query/acc.cgi).

Research involving human participants, their data, or biological material

Policy information about studies with human participants or human data. See also policy information about sex, gender (identity/presentation), and sexual orientation and race, ethnicity and racism.

Reporting on sex and gender	In this study, sex of participants was determined based on self-report. No sex- and gender-based analyses have been performed. We noted the sex of our patients to be able to create a sex-matched group of healthy controls for reference. This was neccessary to exclude possible influences not relating to our analysis.
Reporting on race, ethnicity, or other socially relevant groupings	In this study, race or ethnicity of participants was determined based on self-report. There was no analysis performed based on those parameters.
Population characteristics	Participants were analyzed for their age at sample collection to create age-matched groupings. We also analyzed participants for their current treatments at sample collection.
Recruitment	Healthy controls were recruited on their suitability to create an sex- and age-matched control group for the untreated MS group. Untreated MS patients were recruited after informed consent and included when the patient did not receive any treatment influencing the immune system for the last 6 months and were free of current relapses.
Ethics oversight	Ethics committee of the University Medicine of Göttingen (3/4/14), samples provided by the Charite fall under EA1/362/20

Note that full information on the approval of the study protocol must also be provided in the manuscript.

Field-specific reporting

Please select the one below that is the best fit for your research. If you are not sure, read the appropriate sections before making your selection.

[x] Life sciences [ ] Behavioural & social sciences [ ] Ecological, evolutionary & environmental sciences

For a reference copy of the document with all sections, see nature.com/documents/nr-reporting-summary-flat.pdf

Life sciences study design

All studies must disclose on these points even when the disclosure is negative.

Sample size	The sample size was not predetermined. Generally, the sample size for in vitro cell culture as well as for ex vivo mouse experiments was 3-4 wells/animals per condition to allow for statistical analysis. Wherever possible data from 2-3 experiments was pooled for the analysis. Untreated MS patient and inebilizumab-treated NMOSD patient samples were acquired throughout the study and as many as possible were included.
Data exclusions	Data from flow cytometry analysis were excluded, if the to be analyzed cell population of a sample was smaller than 100 cells.
Replication	All in vitro and mouse experiments were either pooled from or are representative of at least two independent successful experiments. Patients samples were measured over multiple experiments, always allowing for the age- and sex-matched samples to be measured together.
Randomization	Human samples were allocated by treatment. Mice were allocated by mouse line. In EAE experiments, mice were of equal age and gender and were allocated randomly, but with a consideration to a potential cage effect.
Blinding	We did not apply blinding of the samples in our study since washing of the flow cytometer with water between certain groups (wt mice, CD19-def. mice) was required to prevent sample contamination by potential carry over.

Reporting for specific materials, systems and methods

We require information from authors about some types of materials, experimental systems and methods used in many studies. Here, indicate whether each material, system or method listed is relevant to your study. If you are not sure if a list item applies to your research, read the appropriate section before selecting a response.

Materials & experimental systems

n/a	Involved in the study
	☒ Antibodies
	☒ Eukaryotic cell lines
☒	☐ Palaeontology and archaeology
	☒ Animals and other organisms
☒	☐ Clinical data
☒	☐ Dual use research of concern
☒	☐ Plants

Methods

n/a	Involved in the study
☒	☐ ChIP-seq
	☒ Flow cytometry
☒	☐ MRI-based neuroimaging

Antibodies

Antibodies used

anti-CD3 (clone 145-2C11,#100302) / anti-CD28 (clone 37.51,#102101) BioLegend
CD3-PE, FITC, or BV605(145-2C11; BioLegend, #100308,100306, 100351)
CD3-BUV395 (145-2C11; BD Biosciences, #563565)
CD4-BV510 or FITC (GK1.5; #100449,100406, BioLegend)
CD8-BV421, FITC or PerCP-Cy5.5 (53-6.7; BioLegend,#100738,100706,100734)
CD45R/B220-PE-CY7 (RA3-6B2; BioLegend,#103222)
CD11b-BV510, PE or PE-Cy7 (M1/70; BioLegend, #101263, 101207, 101216)
CD11c-PE/Dazzle (N418; BioLegend, #117348)
CD19-PerCP-Cy5.5, PE or AF647 (1D3; SJ25C1 BioLegend,#152406, 152408, 363040)
CD20-AF647 (SA275A11; BioLegend, #150404)
IgD-BV421 (11-26c.2a ; BioLegend, #405725)
IgM-BUV395 (AF6-78; BD Biosciences, #742349)
Ly6C-BV421 (HK1.4; BioLegend, #128032)
Ly6G-BV785 (1A8; BioLegend, #127645)
MHC II-BV421 or BV785 (M5/114.15.2, BioLegend, #107632, 107645)
MHC II-BUV395 (2G9, BD Biosciences, #569244)
NK1.1-BV605 (PK136; BioLegend, #108740)
CD11a-FITC (M17/4; BioLegend, #101106)
CD25-PE or BV421 (PC61.5; e-Bioscience,#12-0251-83 or PC61 BioLegend, #102033)
CD40-PE-CF594 (3/23; BD Biosciences, #562847)
CD49d-PE (9C10; BioLegend, #103706)
CD69-PE-Cy7 or -BV711 (H1.2F3; BioLegend, #104512, #104537)
CD80-APC (16-10A1; BioLegend, #104714)
CD86-BV421 (GL-1; BioLegend, #105032)
CD154-APC (MR1; BioLegend, #106510)
CD16/CD32 (93; BioLegend, #101333)
CD3-BV510 or BV711 (UCHT1; BioLegend, #300448, 317327)
CD4-PE-Cy7 or BV605 (RPA-T4; BD Bioscience, #560649, BioLegend, #300556)
CD8-FITC or PerCP-Cy5.5 (RPA-T8; BD Bioscience, #561948, 560662)
CD11c-PE/Cy5.5 (3.9, eBioscience, #35-0116-42)
CD14-PerCP-Cy5.5 or BV421 (M5E2; BioLegend, #301824, 301830)
CD14-PE/CF594 (MΦP9, BD Biosciences, #562335)
CD16-PE-Cy7 (3G8; BioLegend, #302016)
CD19-AF647 (SJ25C1; BioLegend, #363039)
CD20-PE (REA780; Miltenyi Biotec, #130-111-338)
CCR4-BV510 (1G1; BD Biosciences, #563066)
CCR6-BV605 (11A9; BD Biosciences, #562724)
CCR7-PE-CF594 (150503; BD Biosciences, #562381)
CD45RO-AF700 (UCHL1; BD Biosciences, #561136)
CXCR3-BV786 (1C6; BD Biosciences, #353737)
CD49d-BV421 (9F10; BioLegend, #303422)
CD69-BV785 (FN50; BioLegend, #310932)
IL-17-PE-Cy7 (BL168; BioLegend, #512315)
IFN-γ-BV421 (B27; BioLegend, #506538)
GM-CSF-PE/Dazzle (BVD2-21C11; BioLegend, #502318)
TNFα-AF700 (Mab11; BD Biosciences, #557996)
CD40-PE/Dazzle (5C3; BioLegend, #334342)
CD80-PE-Cy7 (L307.4; BD Biosciences, #305232)
CD86-BV605 (GL-1; BioLegend, #105037)
CD163-FITC (GHI/61; BioLegend, #333618)
CCR2-BV421 (K036C2; BioLegend, #357209)
CCR5-PerCP-Cy5.5 (J418F1; BioLegend, #359112)

	CX3CR1-PE (K0124E1; BioLegend, #355704)<br>Alexa Fluor 647 mouse IgG1,k isotype ctrl (MOPC-21; BD Biosciences, #557714)<br>Alexa Fluor 700 mouse IgG1,k isotype ctrl (MOPC-21; BD Biosciences, #557882)<br>BV421 mouse IgG1,k isotype ctrl (MOPC-21; BioLegend, #400158)<br>BV510 mouse IgG1,k isotype ctrl (X40; BD Biosciences, #562946)<br>BV605 mouse IgG1,k isotype ctrl (X40; BD Biosciences, #562652)<br>BV785 mouse IgG1,k isotype ctrl (MOPC-21; BioLegend, #400169)<br>BUV395 mouse IgG1,k isotype ctrl (MOPC-21; BD Biosciences, #563547)<br>PE-CF594 mouse IgG2a,k isotype ctrl (G155-178; BD Biosciences, #562306)<br>PE-Cy7 mouse IgG1,k isotype ctrl (MOPC-21; BD Biosciences, #557872)<br>REA control (S) antibody (REA293; Miltenyi Biotec, #130-113-438)
Validation	Antibodies were validated by test stainings, unstained samples, fluorescence minus ones, and/or isotype control antibodies.

Eukaryotic cell lines

Policy information about cell lines and Sex and Gender in Research

Cell line source(s)	iPSC line UMGi130-A clone 8 (isWT11.8) was kindly provided by Dr. Lukas Cyganek.
Authentication	The line was authenticated by the provider.
Mycoplasma contamination	Tested negative for mycoplasma.
Commonly misidentified lines<br>(See ICLAC register)	n/a

Animals and other research organisms

Policy information about studies involving animals; ARRIVE guidelines recommended for reporting animal research, and Sex and Gender in Research

Laboratory animals	Wild type (wt) C57BL/6 mice were purchased from Charles River (Strain Code: 027). MOGp35-55 TCR transgenic 2D2 mice were kindly provided by Dr. Kuchroo (Boston, USA, C57BL/6-Tg(Tcra2D2,Tcrb2D2)1Kuch/J; strain #006912). CD19-cre mice were kindly provided by the AG Lalive (B6.129P2(C)-Cd19tm1(cre)Cgn/J; strain #006785). µMT mice (B6.129S2-Ighmtm1Cgn/J; strain #:002288), MHC IIKO mice (B6.129S2-H2dlAb1-Ea/J; strain #003584), and OVA329-337 TCR transgenic OTII mice (B6.Cg-Tg(TcraTcrb)425Cbn/J; strain #004194) were purchased from Jackson Laboratory. Mice were utilized from birth (microglia generation) up to 6 months of age.
Wild animals	No wild animals were utilized.
Reporting on sex	Sex of the mice was only taken into consideration for experiments requiring EAE induction. EAE-induced mice had to be female.
Field-collected samples	No field collected samples
Ethics oversight	All animal experiments were carried out in accordance with the Central Department for Animal Experiments, University Medical Center, Göttingen and approved by the Office for Consumer Protection and Food Safety of the State of Lower Saxony (protocol number 33.9-42502-04-15/1804, 33.9-42502-04-16/2267, and 33.9-42502-04-21/3680, 33.9-42502-04-20/3489).

Note that full information on the approval of the study protocol must also be provided in the manuscript.

Plants

Seed stocks	n/a
Novel plant genotypes	n/a
Authentication	n/a

Flow Cytometry

Plots

Confirm that:

☒ The axis labels state the marker and fluorochrome used (e.g. CD4-FITC).

☒ The axis scales are clearly visible. Include numbers along axes only for bottom left plot of group (a 'group' is an analysis of identical markers).

☒ All plots are contour plots with outliers or pseudocolor plots.

☒ A numerical value for number of cells or percentage (with statistics) is provided.

Methodology

Sample preparation	PBMCs were isolated after Biocoll gradient centrifugation. Single cell suspensions of murine lymphoid tissues were generated and passed through a 70 µm cell strainer. Murine blood was collected in PBS containing 1 mM EDTA and erythrocytes were lysed using BD Pharm Lysing Buffer. Murine splenic and human blood B cells were purified or removed by MACS separation. Murine and human T cells were isolated by negative MACS separation using the mouse pan T cell isolation kit II (Miltenyi) or the MojoSort™ Human CD3 T Cell Isolation Kit (BioLegend). Bone marrow-derived myeloid cells (BMDM) were generated by isolating the bone marrow from 1-2 femurs of C57Bl/6J or CD19-cre mice and stimulating it with L929 medium (DMEM, 30 % L929 cell-conditioned medium, 10 % fetal calf serum, 5 % horse serum, 50 U/ml penicillin, 50 µg/ml streptomycin,<br>23<br>0.05 mM β-mercaptoethanol at 37 °C and 5 % CO2 for seven days. Adherent BMDMs were harvested using cell scrapers. For the generation of primary microglia, brain cells of new-born to two-day-old C57BL/6J mice were enzymatically isolated with 0.4 mg DNAse I (Roche) and 2.5 % trypsin (Pan Biotech). A mixed glial cell culture was achieved by cultivating the cells in DMEM containing 10 % fetal calf serum, 1% GlutaMax™, 100 U/ml penicillin, and 100 µg/ml streptomycin at 37 °C and 5 % CO2 until confluency. Thereafter, cells were stimulated with a medium containing DMEM, 30% L929 cell-conditioned medium, 10 % fetal calf serum, 100 U/ml penicillin, and 100 µg/ml streptomycin for five days, to gain an enriched microglia culture. To separate primary microglia from other glia cells, microglia were harvested by gentle shaking at 90 rpm for 30 min at 37 °C. The generated cultures contained >97 % microglial cells verified by flow cytometry. Human monocyte-derived macrophages (hMDMs) were generated by isolating PBMCs as mentioned above. Monocytes were isolated by positive selection using CD14 MicroBeads (Miltenyi) according to manufacturer´s instruction. Monocytes were cultured at 37 °C, 5 % CO2 for 5 days in medium (AIM V™ medium, 10 % human serum, 50 U/ml penicillin, 50 µg/ml streptomycin), containing 50 ng/ml human M-CSF, followed by 2 days in medium (AIM V™ medium, 10 % human serum, 50 U/ml penicillin, 50 µg/ml streptomycin), containing 10 ng/ml human IFN-γ. Human induced pluripotent stem cells (hiPSCs) maintained in StemMACS™ iPSC-Brew XF (Brew; Miltenyi) were dissociated using Accutase to generate a single-cell suspension. 60 000 cells/cm² were plated in Brew containing 7.5 ng/ml Activin A (Proteintech), 30 ng/ml BMP4 (Proteintech), 3 µM CHIR 99021 (Selleckchem), and 10 µM ROCK inhibitor (Y-27632; Selleckchem) onto Matrigel (Corning)-coated plates. After 18 h, medium was changed to TeSR™-E6 (E6; Stemcell) containing 10 ng/ml Activin A, 40 ng/ml BMP4, and 20 µM IWP2 (Selleckchem). On day 2, medium was changed to E6-containing 10 ng/ml Activin A, 40 ng/ml BMP4, 20 µM IWP2, and 20 ng/ml FGF-basic (Peprotech). On day 3, the cells were dissociated by Accutase and replated at 60 000 cells/cm² onto Matrigel-coated plates in E6-containing 15 ng/ml VEGF 165 (Peprotech), 5 ng/ml FGF-basic, and 10 µM ROCK inhibitor (Y-27632). On day 4, medium was changed to E6-containing 15 ng/ml VEGF 165 and 5 ng/ml FGF-basic. On days 5 and 6, medium was changed to E6-containing 15 ng/ml VEGF 165 and 5 ng/ml FGF-basic, 200 ng/ml SCF (Proteintech), and 20 ng/ml IL-6 (Proteintech). On days 7 and 9, E6-containing 100 ng/ml SCF, 10 ng/ml IL-6, 30 ng/ml TPO (Proteintech), and 30 ng/ml IL-3 (Proteintech). On day 10, the cells in semi-suspension were collected and plated at 10 500 cells/cm² onto Matrigel-coated plates in iMG medium [75 % IMDM (Gibco™), 25 % F12 (Gibco™) medium, containing 1x B-27 supplement (Gibco™), 1x GlutaMAX™ supplement (Gibco™), 100 ng/ml IL-34 (Proteintech), and 20 ng/ml M-CSF (Proteintech)]. New iMG medium was supplemented every second day. On day 21, the cells were mechanically dissociated and replated at 10 500 – 20 800 cells/cm² onto Matrigel-coated plates in iMG medium. New iMG medium was supplemented every second day. On day 28, the cells were mechanically dissociated and replated at 20 800 – 52 000 cells/cm² onto Matrigel-coated plates in iMG medium. Half medium changes were performed every second day until final analysis. Fc receptors were blocked using a monoclonal antibody specific for CD16/CD32 (Human TruStain FcX™ (Fc Receptor Blocking Solution); BioLegend). Dead human and murine cells were stained with LIVE/DEAD™ Fixable NIR Dead Cell Stain Kit (Thermo Fisher Scientific).
Instrument	BD™ LSRII cytometer (BD Bioscience)
Software	BD FACSDiva™ software and FlowJo software (FlowJo LLC)
Cell population abundance	The abundance of cells per population ranged from <100 (excluded for further analysis) to 100 000 cells. Purity was determined by flow cytometry.
Gating strategy	Pregating was performed by FSC-A/SSC-A for size, FSC-A/FSC-H for single cells followed by Zombie/FSC-A to exclude zombie positive necrotic cells. When gating for CD19 positive cells apart from B cells, B cells were excluded in a pregate by either CD20 or B220.

☒ Tick this box to confirm that a figure exemplifying the gating strategy is provided in the Supplementary Information.
