# Supplementary material for: B cell CD19 is transferred between immune cells in mice and humans
Source: Nat Commun. 2026 Jul 29;17:7588. doi: 10.1038/s41467-026-75534-3 (PMC13421502; doi:10.1038/s41467-026-75534-3)
Supplement: Supplementary file 2 — Transparent Peer Review file [file 41467_2026_75534_MOESM2_ESM.pdf]

nature portfolio

Peer Review File

B cell CD19 is widely transferred between immune cells

Corresponding Author: Professor Martin Weber

This file contains all reviewer reports in order by version, followed by all author rebuttals in order by version.

Version 0:

Reviewer comments:

Reviewer #1

(Remarks to the Author)
This manuscript by Ochs et al. demonstrates that T cells and myeloid cells acquire CD19 from B cells via trogocytosis during T-B or myeloid-B cell interactions, which is an extension of their previous report that T cells acquire CD20 from B cells (Ochs et al., Sci. Transl. Med., 2022). As in that report, the authors here clearly demonstrate T cell and myeloid cell trogocytosis of CD19 in several experimental settings in both mouse and human systems. However, the physiological relevance of CD19 on T or myeloid cells, or of CD19+ these cells is not addressed at all. For instance, as the authors discussed (lines 486-487), it is quite important to address whether depletion of CD19+ T cells by anti-CD19 mAb is beneficial in neuromyelitis optica therapy using a mouse model.

It has been well known for over 20 years that CD4+ T cell receptor mediates trogocytosis of MHC class II molecules and bystander membrane proteins, including CD80, CD86, and CD54, from antigen-presenting cells (Hwang et al., J. Exp Med., 2000; Tatari-Calderone et al., J. Immunol., 2002; Wetzel et al., 2005, J. Immunol.; Zhou et al., Blood, 2005). Recently, trogocytosis of CD19 has raised concerns for CAR-T therapy (Hamieh et al., Nature, 2019; Labanieh and Mackall, Nature, 2023). Therefore, the intercellular transfer of membrane proteins between immune cells has been well recognized. The manuscript by Ochs et al. could be the first report that T cells trogocytose CD19 from B cells; however, this observation alone is not sufficiently informative and therefore does not meet the criteria for publication in Nature Communications.

Reviewer #2

(Remarks to the Author)
This paper strongly supports the high transfer of membrane and membrane-bound molecules during cell-cell interactions, particularly between B and T cells by trogocytosis, but also between phagocytes and B cells by efferocytosis, resulting in pro-inflammatory phenotypes of T cells and myeloid cells. The experimental strategies are well conducted and illustrated in mouse and murine models of EAE and in vivo cocultures, and are very convincing.
We therefore understand that (i) CD19 is not expressed endogenously in T cells, (ii) B cells are required to obtain CD19+ T cells, (iii) transfer from B cells to T cells requires direct cell-cell interaction, (iv) that CD19+T cells exert both pro-inflammatory (especially GM-CSF and anti-IL17 and TNF production) and anti-inflammatory (IL10 production) capacities, (v) that efferocytosis also enables myeloid cells to integrate membrane and membrane-bound molecules such as CD19 from apoptotic B cells, and potentially to engage in pro-inflammatory actions.

The authors also provide some human data. The authors also show the presence of 8-10% CD19+T cells in healthy human PBMCs, with also some in vitro properties to exert Th1, Th2, Th17 and cytotoxic phenotypes and properties. Comparisons between healthy and MS patients show that CD4+CD19+T cells are more inflammatory in MS, but CD8+CD19+T cells are not. The authors illustrate the depletion of C19+T cells by inebilizumab in 3 patients with NMOSD treated with inebilizumab. The results are significant and show how B-cell depletion can have an impact on targeted cells, but also more generally, suggesting new pathophysiological approaches and therapeutic aspects.

Main comments and questions:
The authors should explain or discuss why they use 2 EAE active models with MOG35-55 and MOG1-117. Are both models associated with the same magnitude of B cell response?
Given the human data are limited to a small sample of MS and NMOSD patients, it would be interesting to add longitudinal information. Do the authors have the number and percentage of NMOSD patients' CD19+ T cells among T cells (i.e., just

before the introduction of inebilizumab) or at least lymphocyte immunophenotyping at baseline with the number of total, CD4+ and CD8+ T cells. This would make possible to measure whether or not inebilizumab has a significant impact on T cells in the blood.
The authors should also discuss how such expected selective depletive therapy may impact the risk of the treatments especially on infection risk and immunosurveillance.

Reviewer #3

(Remarks to the Author)
This manuscript investigates the transfer of CD19 from B cells to T cells, utilizing both murine and human cell models. This transfer occurs via trogocytosis, a process by which membrane fragments and surface molecules are exchanged between cells in close contact. The significance of this finding lies in its implications for therapies that employ anti-CD19 antibodies to deplete B cells. Such treatments may inadvertently lead to the depletion of CD19-acquiring T cell populations.
A limitation of this manuscript is the substantial overlap with a previously published study (PMID: 35353539) by the same research team. That study described the transfer of CD20 from B cells to T cells using similar experimental methods and approaches. The current manuscript replicates the analysis to include CD19, with many of the methodologies and experimental design largely unchanged.
Nevertheless, this study does extend the concept of B cell marker trogocytosis, and its consequence for B cell-targeted therapies, to include monocytes. It also provides mechanistic insights into the process by which CD19 is transferred to monocytes.

The experiments conducted are of high quality and support the conclusions of the study.
Specific comments
Figure 1 and Figure 6 would benefit from inclusion of flow cytometry to show CD19 levels on B cells.
Figure 2. Why are %CD19+ T cells in i and j so low compared to the higher % depicted in Figure 1?

Version 2:

Reviewer comments:

Reviewer #1

(Remarks to the Author)
The original manuscript by Ochs et al. lacked experiments addressing the function of B cell-derived CD19-acquired cells, which was my major concern. In the revised manuscript, the authors have adequately addressed this issue by demonstrating that anti-IgM stimulation activates CD19/IgM/IgD-acquired T cells (Fig. 5) and myeloid cells (Fig. 9). Although the in vivo pathogenic role of these cells remained to be established in an appropriate mouse model, the authors discuss this possibility appropriately based on their in vitro studies using patient-derived cells. Overall, the revised manuscript satisfactory address the major concerns raised during review.

Reviewer #2

(Remarks to the Author)
I have no additionnal comments, thanks to the authors for the effort and the supplementary data

Reviewer #3

(Remarks to the Author)
The revised manuscript is significantly strengthened and I support publication.

Open Access This Peer Review File is licensed under a Creative Commons Attribution 4.0 International License, which permits use, sharing, adaptation, distribution and reproduction in any medium or format, as long as you give appropriate credit to the original author(s) and the source, provide a link to the Creative Commons license, and indicate if changes were made.
In cases where reviewers are anonymous, credit should be given to 'Anonymous Referee' and the source.
The images or other third party material in this Peer Review File are included in the article’s Creative Commons license, unless indicated otherwise in a credit line to the material. If material is not included in the article’s Creative Commons license and your intended use is not permitted by statutory regulation or exceeds the permitted use, you will need to obtain permission directly from the copyright holder.
To view a copy of this license, visit https://creativecommons.org/licenses/by/4.0/

Response to reviewers

In response to the instructive critique by the reviewers, we performed additional experiments which are included in this revision and described in detail below. We thank the reviewers for raising these important points and believe that adding the requested data substantially improved the quality of our work. In the following, we provide our responses to each of the reviewers' comments:

Reviewer: 1

This manuscript by Ochs et al. demonstrates that T cells and myeloid cells acquire CD19 from B cells via trogocytosis during T-B or myeloid-B cell interactions, which is an extension of their previous report that T cells acquire CD20 from B cells (Ochs et al., Sci. Transl. Med., 2022). As in that report, the authors here clearly demonstrate T cell and myeloid cell trogocytosis of CD19 in several experimental settings in both mouse and human systems. However, the physiological relevance of CD19 on T or myeloid cells, or of CD19+ these cells is not addressed at all. For instance, as the authors discussed (lines 486-487), it is quite important to address whether depletion of CD19+ T cells by anti-CD19 mAb is beneficial in neuromyelitis optica therapy using a mouse model.

We thank the reviewer for these thoughtful points. Indeed, it is quite important to know if $CD19^+$ cells are pathogenic and if their depletion is beneficial in neuromyelitis optica (NMO) therapy. We could show that, just like $CD20^+$ T cells which we found to be pathogenic, $CD19^+$ T cells are differentiated cells, expressing high amounts of adhesion molecules and proinflammatory cytokines. Therefore, we see a considerable pathogenic potential for these cells and are confident that their depletion will be beneficial in NMO therapy. In our search for strategies to deplete $CD19^+$ T cells, we purchased a murine anti-CD19 B cell-depleting antibody from Bio X Cell, LLC (#BE0150). This antibody requires a secondary anti-rat antibody which we also purchased (#BE0122). We followed the depletion protocol provided by the publications referenced by Bio X Cell, LLC [1, 2], (Fig. 1a). To test this antibody combination, we first performed experiments to deplete all $CD19^+$ cells to prove their depletion capacity. We performed the test-depletion experiment twice with an isotype control antibody, followed by the secondary antibody, as negative control and once with an established anti-CD20 antibody as positive control. Unfortunately, the anti-CD19 antibody did not deplete neither B cells nor $CD19^+$ T cells (Fig. 1b-e). We were unable to find any other, commercially available anti-CD19-depleting antibody. Bio X Cell, LLC suggests to achieve B cell depletion by combining the anti-CD19 antibody with their anti-CD22 and anti-B220 antibodies, however they have no suggestion on how to improve anti-CD19 single antibody depletion. Another strategy we considered is $CD19^+$ T cell depletion via inebilizumab therapy in mice genetically modified to express human CD19 on their B cells, however there is no such huCD19tg mouse line commercially available.

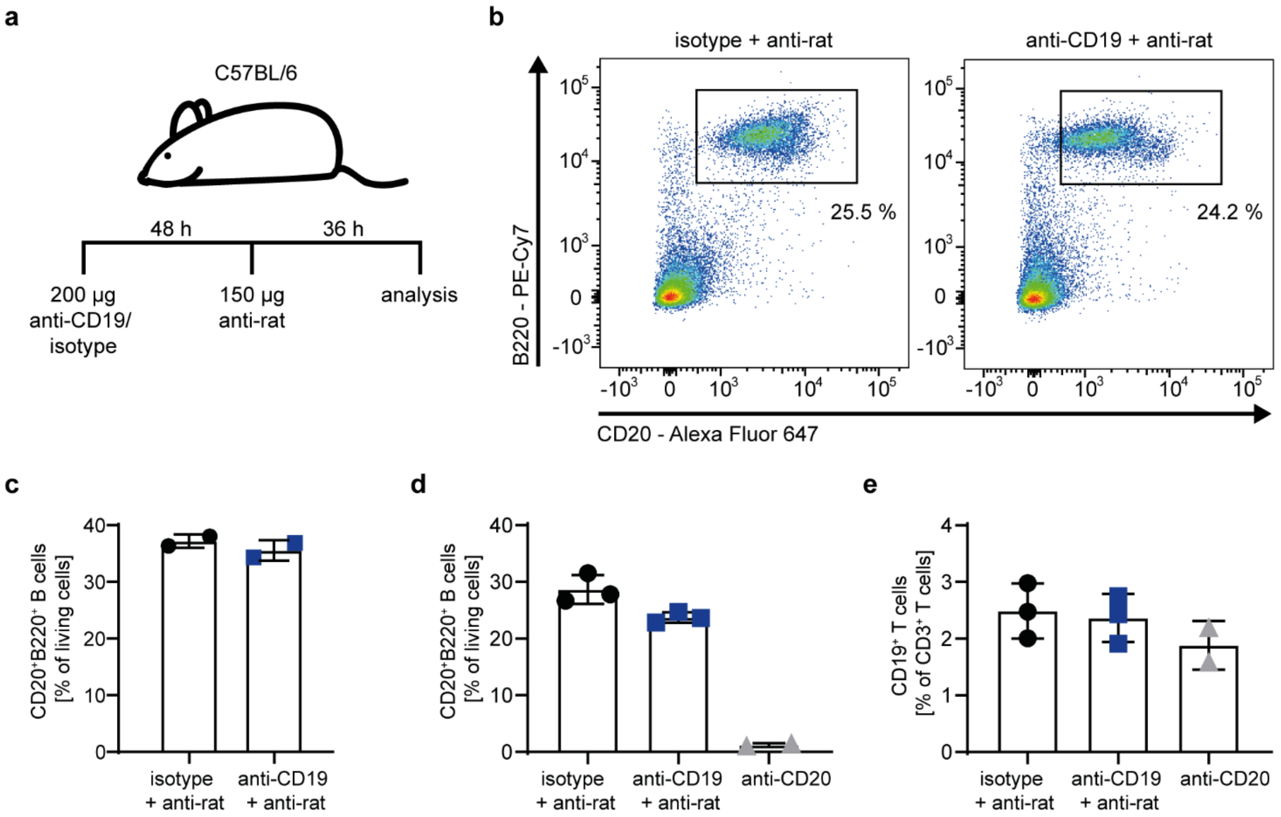

Fig. 1: Anti-CD19 depletion in mouse
a Schematic representation of the experimental design. **b** Representative flow cytometry blots for splenocytes after treatment. **c, d** Analysis of B cells in the spleen after treatments in two separate experiments. **c** Experiment 1; n = 2 mice per group. **d** Experiment 2; n = 2-3 mice per group. **e** Analysis of CD19$^+$ T cells in the spleen after treatments in two separate experiments; n = 2-3 mice per group.

It has been well known for over 20 years that CD4+ T cell receptor mediates trogocytosis of MHC class II molecules and bystander membrane proteins, including CD80, CD86, and CD54, from antigen-presenting cells (Hwang et al., J. Exp Med., 2000; Tatari-Calderone et al., J. Immunol., 2002; Wetzel et al., 2005, J. Immunol.; Zhou et al., Blood, 2005). Recently, trogocytosis of CD19 has raised concerns for CAR-T therapy (Hamieh et al., Nature, 2019; Labanieh and Mackall, Nature, 2023). Therefore, the intercellular transfer of membrane proteins between immune cells has been well recognized. The manuscript by Ochs et al. could be the first report that T cells trogocytose CD19 from B cells; however, this observation alone is not sufficiently informative and therefore does not meet the criteria for publication in Nature Communications.

We thank the reviewer for the careful contextualization of our findings. We are aware of the mentioned references and appreciate the acknowledgement that we are the first to report the transfer of CD19 to T cells and monocytes.

Reviewer #2:

This paper strongly supports the high transfer of membrane and membrane-bound molecules during cell-cell interactions, particularly between B and T cells by trogocytosis, but also between phagocytes and B cells by efferocytosis, resulting in pro-inflammatory phenotypes of T cells and myeloid cells. The experimental strategies are well conducted and illustrated in mouse and murine models of EAE and in vivo cocultures, and are very convincing.
We therefore understand that (i) CD19 is not expressed endogenously in T cells, (ii) B cells are required to obtain CD19+ T cells, (iii) transfer from B cells to T cells requires direct cell-cell interaction, (iv) that CD19+T cells exert both pro-inflammatory (especially GM-CSF and anti-IL17 and TNFα production) and anti-inflammatory (IL10 production) capacities, (v) that efferocytosis also enables myeloid cells to integrate membrane and membrane-bound molecules such as CD19 from apoptotic B cells, and potentially to engage in pro-inflammatory actions.

The authors also provide some human data. The authors also show the presence of 8-10% CD19+T cells in healthy human PBMCs, with also some in vitro properties to exert Th1, Th2, Th17 and cytotoxic phenotypes and properties. Comparisons between healthy and MS patients show that CD4+CD19+T cells are more inflammatory in MS, but CD8+CD19+T cells are not. The authors illustrate the depletion of C19+T cells by inebilizumab in 3 patients with NMOSD treated with inebilizumab.
The results are significant and show how B-cell depletion can have an impact on targeted cells, but also more generally, suggesting new pathophysiological approaches and therapeutic aspects.

We thank this reviewer for the excellent summary of our findings and value the careful wording on the significance and impact of our work.

Main comments and questions:

The authors should explain or discuss why they use 2 EAE active models with MOG35-55 and MOG1-117. Are both models associated with the same magnitude of B cell response?

We thank the reviewer for raising this important point. The two active EAE models indeed differ in their B cell response. The MOG_{35-55} peptide model can operate largely without any B cell involvement, while the MOG_{1-117} protein model is considered a B cell-involving EAE model with a strong B cell activation. This difference can also explain the higher amount of transferred CD19 to T cells in the MOG_{1-117} protein EAE model compared to the MOG_{35-55} peptide EAE (Fig. 3a-d). Based on these differences in regard to pathogenic B cell contribution, we employed both models. We apologize this important point was not clear enough and clarified this point by rephrasing the respective part in the results as follows:

“Therefore, we immunized wild type mice with MOG_{35-55} peptide (B cell-independent model) or MOG_{1-117} protein (B cell-involving model) to induce EAE and discovered an increase of $CD19^+$ T cells in the immunization draining lymph nodes, comparable to that previously found for $CD20^+$ T cells (Fig. 3a-d). The increase in CD19 on T cells is more pronounced in the B cell-involving model, indirectly confirming the finding that pathogenic T cells are activated by B cells.”

Given the human data are limited to a small sample of MS and NMOSD patients, it would be interesting to add longitudinal information. Do the authors have the number and percentage of NMOSD patients' CD19+ T cells among T cells (i.e., just before the introduction of inebilizumab) or at least lymphocyte immunophenotyping at baseline with the number of total, CD4+ and CD8+ T cells. This would make possible to measure whether or not inebilizumab has a significant impact on T cells in the blood.

We thank the reviewer for raising this important question. In response, we are now providing all available details on the inebilizumab-treated patient samples. Out of the three samples we analyzed, one patient did not have any lymphocyte immunophenotyping before inebilizumab treatment. The other two patients had immunophenotyping prior to inebilizumab initiation. We provide this additional information below in table 1. However, we feel that the data is difficult to interpret, as both patients received various immunosuppressing medications before starting inebilizumab.

Table 1: Absolute amount/percentage of T cells in inebilizumab-treated NMOSD patients

	**before inebilizumab**	**after/during inebilizumab**
**patient 1**	not measured	1.91 /nl (86 %)
**patient 2**	2.32 /nl (95 %)	1.18 /nl (94 %)
**patient 3**	1.18 /nl (94 %)	1.51 /nl (92 %)

To address mechanistically whether inebilizumab is capable of depleting $CD19^+$ T cells and monocytes, we modelled *in vivo* depletion in an *in vitro* setting. For this purpose, we isolated peripheral mononuclear blood cells from three healthy controls and treated the cells with inebilizumab or an isotype control antibody *in vitro* overnight. $CD19^+$ T cells and monocytes of healthy controls seem to be reduced by inebilizumab treatment *in vitro* (Fig. 2).

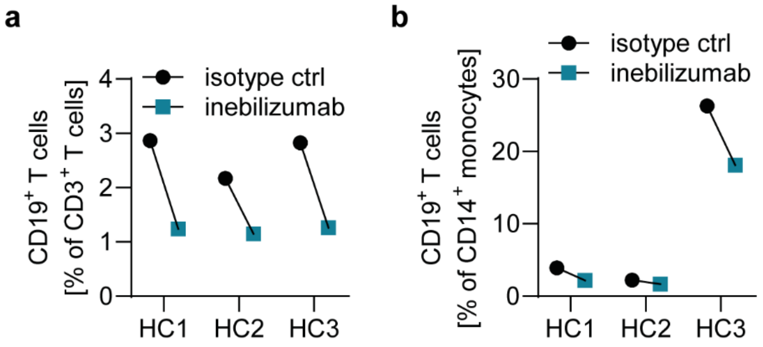

Fig. 2: *In vitro* depletion with inebilizumab
a, b Peripheral mononuclear blood cells were isolated for three healthy controls (HC) and incubated overnight with 1 µg/ml inebilizumab or an isotype control antibody *in vitro*; 2 wells were employed per HC per condition with the mean of both cultures displayed.

The authors should also discuss how such expected selective depletive therapy may impact the risk of the treatments especially on infection risk and immunosurveillance.

We agree that this important point needs to be discussed. We assume that anti-CD19 depletion of T cells and monocytes may have negative effects on infection risk and immunosurveillance, since recently B cell-activated T cells acquiring CD19 are unselectively depleted. This may include T cell populations raised to counteract infections. To include this critical point in our manuscript we added the following passage into our discussion (line 492-494):

"However, the clinical impact of anti-CD19 depleting $CD19^+$ T cells may be limited by the turnover of plasma membranes and thus the accessibility of membrane embedded CD19 on T cells. In return, this aspect on membrane turnover could be an advantage in regard to immunosurveillance, as inebilizumab may also deplete $CD19^+$ T cells raised to counteract infections."

Reviewer #3:

This manuscript investigates the transfer of CD19 from B cells to T cells, utilizing both murine and human cell models. This transfer occurs via trogocytosis, a process by which membrane fragments and surface molecules are exchanged between cells in close contact. The significance of this finding lies in its implications for therapies that employ anti-CD19 antibodies to deplete B cells. Such treatments may inadvertently lead to the depletion of CD19-acquiring T cell populations.
A limitation of this manuscript is the substantial overlap with a previously published study (PMID: 35353539) by the same research team. That study described the transfer of CD20 from B cells to T cells using similar experimental methods and approaches. The current manuscript replicates the analysis to include CD19, with many of the methodologies and experimental design largely unchanged.

Nevertheless, this study does extend the concept of B cell marker trogocytosis, and its consequence for B cell-targeted therapies, to include monocytes. It also provides mechanistic insights into the process by which CD19 is transferred to monocytes.

We acknowledge that our recent manuscript provides some complementary data in regard to B cell-T cell interaction. We appreciate that the reviewer values that the current manuscript is a substantial extension on the concept of B cell trogocytosis and its consequences and the inclusion of monocytes. We thank the reviewer for this supportive comment.

The experiments conducted are of high quality and support the conclusions of the study.

Specific comments
Figure 1 and Figure 6 would benefit from inclusion of flow cytometry to show CD19 levels on B cells.

We thank the reviewer for this idea to improve our respective figures and added CD19 levels on B cells to the representative blots shown in Figure 1 (also now included in the manuscript):

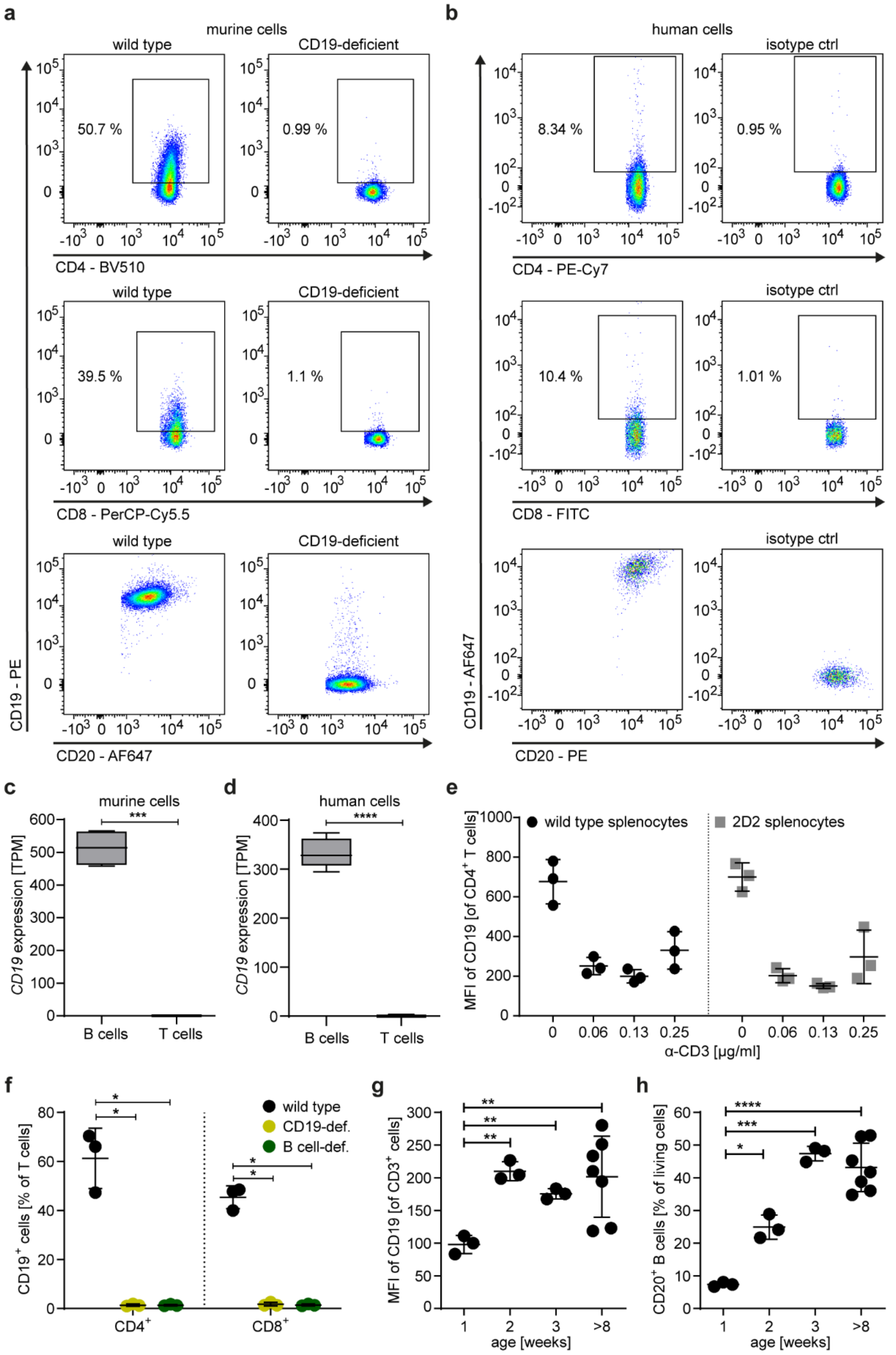

Fig.1: CD19 can be found on T cells but is not endogenously expressed

a, b Representative flow cytometric staining of CD19 on CD4+ and CD8+ T cells <u>and B cells</u> isolated from

a wild type and CD19-deficient CD19-cre mice or **b** healthy control peripheral blood mononuclear cells (PBMCs). **c, d** Bulk RNA sequencing of fluorescence-activated cell (FACS)-sorted B and T cells for the *CD19* transcript shown as TPM, reads per kilobase of transcript per million reads mapped; **c** n = 3-8 mice, analyzed via unpaired Student's t test with Welch's correction and Bonferroni correction for multiple testing; **d** n = 4 healthy human donors; one set of B and T cells per mouse or human, analyzed via Mann Whitney test with Bonferroni correction for multiple testing; displayed as box plot with means ± SD; **e** Mean fluorescence intensity (MFI) of CD19 of $CD4^+$ T cells of splenocyte cultures isolated from wild type mice or myelin oligodendrocyte glycoprotein $(MOG)_{35-55}$ peptide T cell receptor (TCR) transgenic 2D2 mice stimulated with anti-CD3/anti-CD28 antibodies for 48 hours. **f** Flow cytometric analysis of CD19 on $CD4^+$ and $CD8^+$ T cells from the spleens of wild type, CD19-deficient CD19-cre, and B cell-deficient µMT mice; n = 3 per group; analyzed via Brown-Forsythe and Welch ANOVA with Dunnett's T3 multiple comparisons test. **g** MFI of CD19 of $CD3^+$ T cells and **h,** percentage of $CD20^+$ B cells in murine spleens during development; n = 3-7 per group, displayed as means ± SD; analyzed via Brown-Forsythe and Welch ANOVA with Holm-Sidak's multiple comparisons test. All figures are representative of **(a, b, e-h)** or pooled from **(c, d)** 2-4 independent experiments; data displayed as means ± SD.

Figure 2. Why are %CD19+ T cells in i and j so low compared to the higher % depicted in Figure 1?

We agree that this requires clarification. The cells from Fig. 1 are stained directly after isolation from the blood. In contrast, the cells from Fig. 2i, j have gone through magnetic-activated cell sorting and were afterwards incubated with or without Staphylococcus enterotoxin B (SEB) for 48 h. The differences in CD19 positivity can be explained due to the separation process and activation of the cells. Another factor may be membrane turnover. A T cell exchanges its whole surface in the course of 24-48 h. Therefore, if T cells do not receive new CD19 via T cell-B cell interaction, they will inevitably loose it over time. Taken together these points hopefully explain the discrepancy in the amount of $CD19^+$ T cells between the signals displayed in Fig. 1 and Fig. 2.

We thank you for your kind attention and look forward to your response.

Sincerely,

Martin S. Weber, M.D.

1. Sawen P, Lang S, Mandal P, Rossi DJ, Soneji S, Bryder D. Mitotic History Reveals Distinct Stem Cell Populations and Their Contributions to Hematopoiesis. *Cell Rep* **14**, 2809-2818 (2016).

2. Keren Z, *et al.* B-cell depletion reactivates B lymphopoiesis in the BM and rejuvenates the B lineage in aging. *Blood* **117**, 3104-3112 (2011).

RESPONSE TO REVIEWERS' COMMENTS

In response to the instructive critique by the reviewers, we performed additional experiments which are included in this revision and described in detail below. We thank the reviewers for raising these important points and believe that adding the requested data substantially improved the quality of our work. In the following, we provide our responses to each of the reviewers' comments:

Reviewer: 1

This manuscript by Ochs et al. demonstrates that T cells and myeloid cells acquire CD19 from B cells via trogocytosis during T-B or myeloid-B cell interactions, which is an extension of their previous report that T cells acquire CD20 from B cells (Ochs et al., Sci. Transl. Med., 2022). As in that report, the authors here clearly demonstrate T cell and myeloid cell trogocytosis of CD19 in several experimental settings in both mouse and human systems. However, the physiological relevance of CD19 on T or myeloid cells, or of CD19+ these cells is not addressed at all.

We thank the reviewer for pointing us to this very interesting question. Additional experiments now revealed the physiological relevance of the observation that CD19 is transferred to T and myeloid cells. In these new experiments, we show that CD19 has a high probability of being co-transferred with other B cell-specific antigens, such as IgD and IgM (Fig. 1a,b and Fig.2a,b). These molecules could be detected on T and myeloid cells almost exclusively when they also contained CD19. Most importantly, we show that transferred molecules are functional in the receiving cell via stimulating $CD19^{+}$ and $CD19^{-}$ T and myeloid cells separately with a B cell-specific stimulus (anti-IgM/G Fab2-Fragment). We could observe a significant activation in the $CD19^{+}$ T and myeloid cells compared to the $CD19^{-}$ counterparts. These results establish that via trogocytosis and efferocytosis functional B cell-properties, so far assumed to be exclusively carried out by B cells, can be transferred to T and myeloid cells (Fig. 1c,d and Fig. 2c-f).

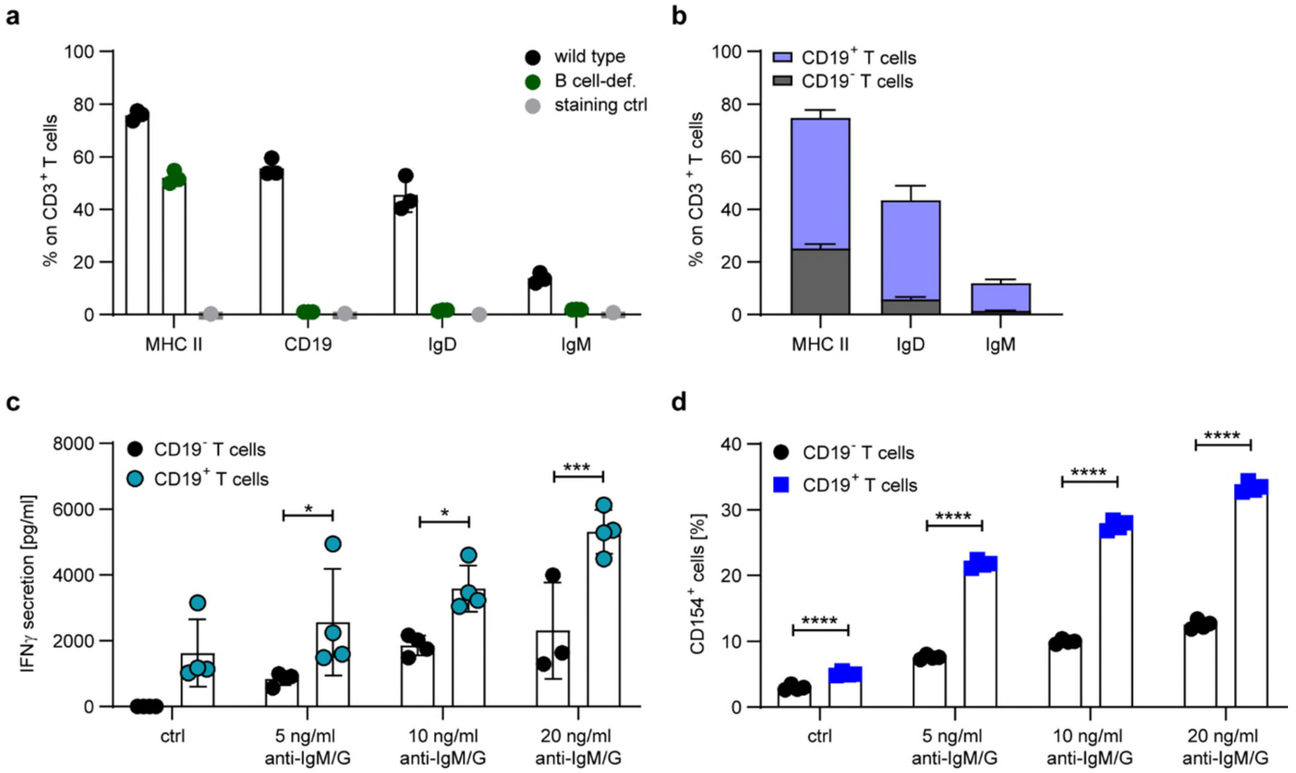

Fig.1: CD19$^+$ T cells can be activated by B cell-stimulation. a Flow cytometric analysis of B cell marker on CD3$^+$ T cells from the spleens of wild type and B cell-deficient µMT mice; n = 3 per group; either isotype control or fluorescence minus one served as staining control. **b** B cell marker positive cells from a were analyzed for their double positivity with CD19. **c** ELISA-based cytokine analysis for IFN-γ of the supernatant of fluorescence-activated cell sorted CD19$^+$ and CD19$^-$ T cells from the spleens of naive mice stimulated with anti-IgM/IgG F(ab′)2 fragment; n = 3-4 wells per group analyzed via 2-way ANOVA with Sidak's multiple comparisons test; data is displayed as means ± SD. **d** Flow cytometric analysis of fluorescence-activated cell sorted CD19$^+$ and CD19$^-$ T cells from the spleens of naive mice stimulated with anti-IgM/IgG F(ab′)2 fragment; n = 3-4 wells per group analyzed via 2-way ANOVA with Sidak's multiple comparisons test; data is displayed as means ± SD.

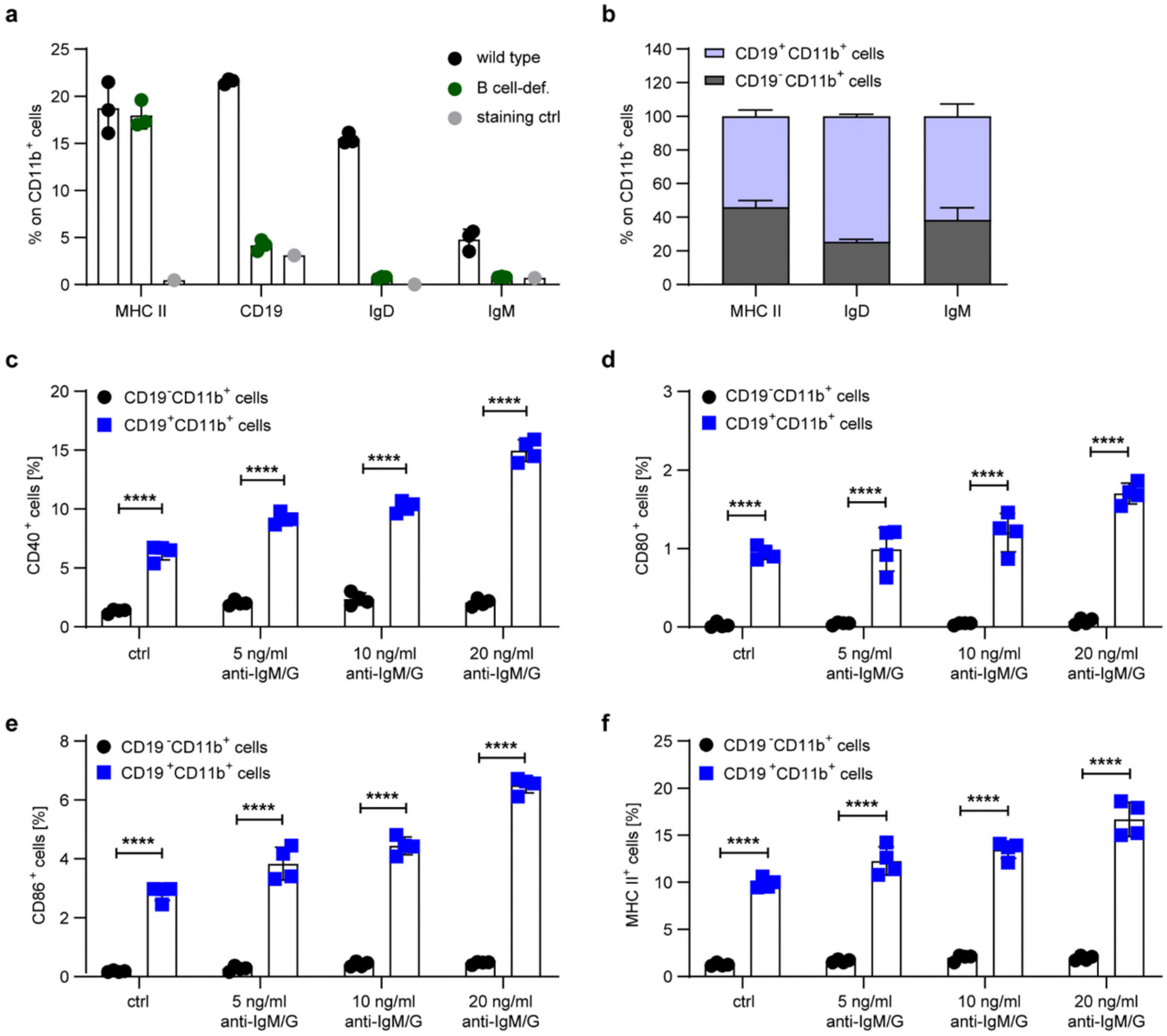

Fig.2: CD19⁺ myeloid cells can be stimulated via the B cell receptor. a Flow cytometric analysis of B cell marker on CD11b⁺ cells from the spleens of wild type and B cell-deficient μMT mice; n = 3 per group; either isotype control or fluorescence minus one served as staining control. **b** B cell marker positive cells from a were analyzed for their double positivity with CD19. **c-f** Flow cytometric analysis of fluorescence-activated cell sorted CD19⁺ and CD19⁻ CD11b⁺ cells from the spleens of naive mice stimulated with anti-IgM/IgG F(ab′)2 fragment; n = 3-4 wells per group analyzed via 2-way ANOVA with Sidak's multiple comparisons test; data is displayed as means ± SD.

For instance, as the authors discussed (lines 486-487), it is quite important to address whether depletion of CD19+ T cells by anti-CD19 mAb is beneficial in neuromyelitis optica therapy using a mouse model.

We thank the reviewer for these thoughtful points. Indeed, it is quite important to know if CD19⁺ cells are pathogenic and if their depletion is beneficial in neuromyelitis optica (NMO) therapy. In that regard, we could show that CD19⁺ T cells are highly differentiated effector cells, expressing high

amounts of adhesion molecules and proinflammatory cytokines. Therefore, we see a considerable pathogenic potential for these cells and are confident that their depletion will be beneficial in NMO therapy. We would indeed like to address this question furthermore in an applicable mouse model. However, currently there is no anti-CD19-depleting antibody that can be used in mice available to test the therapeutic depletion effect in such a mouse experiment. Based on this situation, we tried to model an *in vivo* anti-CD19 depletion in an *in vitro* setting. For this purpose, we isolated peripheral mononuclear blood cells from three healthy controls and treated the cells with inebilizumab or an isotype control antibody *in vitro* overnight. $CD19^+$ T cells and monocytes of healthy controls seem to be reduced by inebilizumab treatment *in vitro* (Fig. 3). In addition, we show in the manuscript Fig.6i and 7h that $CD19^+$ T cells and monocytes are depleted by inebilizumab in patients with MS. In light of the pathogenic phenotype of $CD19^+$ T cells and monocytes shown both in mice and humans, we thus consider it highly plausible that their concomitant depletion is beneficial in treatment of NMOSD.

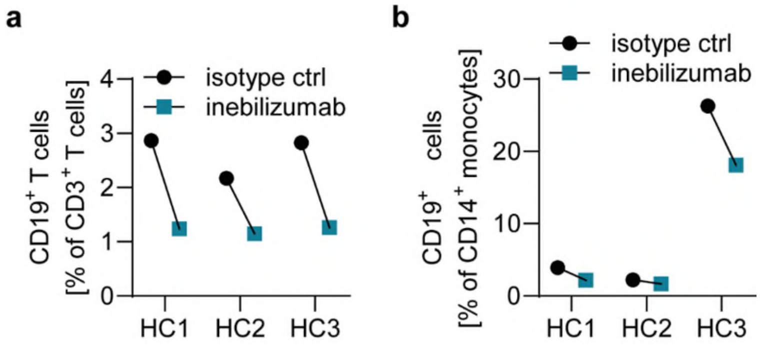

Fig. 3: *In vitro* depletion with inebilizumab
a, b Peripheral mononuclear blood cells were isolated for three healthy controls (HC) and incubated overnight with 1 µg/ml inebilizumab or an isotype control antibody *in vitro*; 2 wells were employed per HC per condition with the mean of both cultures displayed.

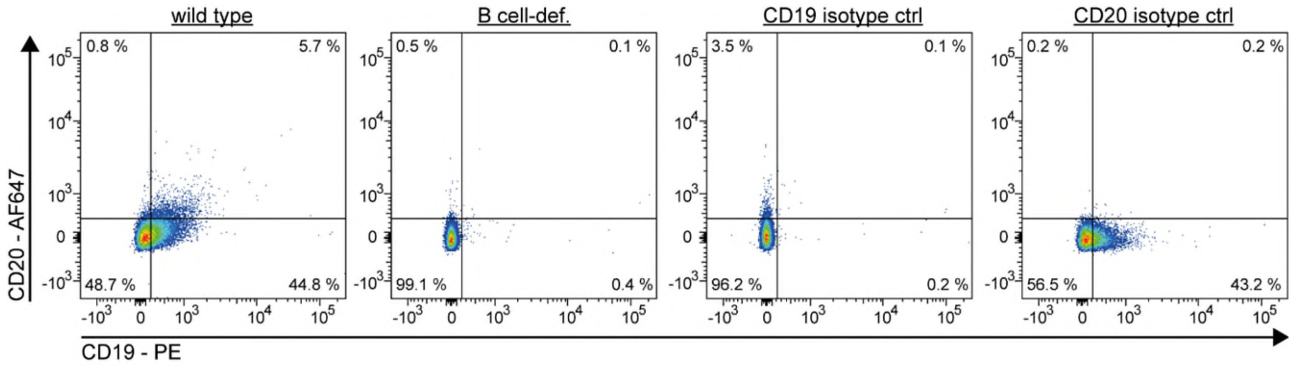

Fig. 4: *Exemplary flow cytometric blots of murine T cells*
Murine splenocytes isolated from wild type or B cell-deficient µMT mice stained for CD3, CD19, and CD20 and controlled by CD19 and CD20 isotype control antibodies.

It has been well known for over 20 years that CD4+ T cell receptor mediates trogocytosis of MHC class II molecules and bystander membrane proteins, including CD80, CD86, and CD54, from antigen-presenting cells (Hwang et al., J. Exp Med., 2000; Tatari-Calderone et al., J. Immunol., 2002; Wetzel et al., 2005, J. Immunol.; Zhou et al., Blood, 2005). Recently, trogocytosis of CD19 has raised concerns for CAR-T therapy (Hamieh et al., Nature, 2019; Labanieh and Mackall, Nature, 2023). Therefore, the intercellular transfer of membrane proteins between immune cells has been well recognized. The manuscript by Ochs et al. could be the first report that T cells trogocytose CD19 from B cells; however, this observation alone is not sufficiently informative and therefore does not meet the criteria for publication in Nature Communications.

We thank the reviewer for the careful contextualization of our findings. We are aware of the mentioned references and appreciate the acknowledgement that we are the first to report the transfer of CD19 to T cells and monocytes.

Reviewer #2:

This paper strongly supports the high transfer of membrane and membrane-bound molecules during cell-cell interactions, particularly between B and T cells by trogocytosis, but also between phagocytes and B cells by efferocytosis, resulting in pro-inflammatory phenotypes of T cells and myeloid cells. The experimental strategies are well conducted and illustrated in mouse and murine models of EAE and in vivo cocultures, and are very convincing.
We therefore understand that (i) CD19 is not expressed endogenously in T cells, (ii) B cells are required to obtain CD19+ T cells, (iii) transfer from B cells to T cells requires direct cell-cell interaction, (iv) that CD19+T cells exert both pro-inflammatory (especially GM-CSF and anti-IL17 and TNFα production) and anti-inflammatory (IL10 production) capacities, (v) that efferocytosis also enables myeloid cells to integrate membrane and membrane-bound molecules such as CD19 from apoptotic B cells, and potentially to engage in pro-inflammatory actions.

The authors also provide some human data. The authors also show the presence of 8-10% CD19+T cells in healthy human PBMCs, with also some in vitro properties to exert Th1, Th2, Th17 and cytotoxic phenotypes and properties. Comparisons between healthy and MS patients show that CD4+CD19+T cells are more inflammatory in MS, but CD8+CD19+T cells are not. The authors illustrate the depletion of C19+T cells by inebilizumab in 3 patients with NMOSD treated with inebilizumab.
The results are significant and show how B-cell depletion can have an impact on targeted cells, but also more generally, suggesting new pathophysiological approaches and therapeutic aspects.

We thank this reviewer for the excellent summary of our findings and value the careful wording on the significance and impact of our work.

Main comments and questions:

The authors should explain or discuss why they use 2 EAE active models with MOG35-55 and MOG1-117. Are both models associated with the same magnitude of B cell response?

We thank the reviewer for raising this important point. The two active EAE models indeed differ in their B cell response. The MOG_{35-55} peptide model can operate largely without any B cell involvement, while the MOG_{1-117} protein model is considered a B cell-involving EAE model with a strong B cell activation. This difference can also explain the higher amount of transferred CD19 to T cells in the MOG_{1-117} protein EAE model compared to the MOG_{35-55} peptide EAE (Fig. 3a-d). Based on these differences in regard to pathogenic B cell contribution, we employed both models. We apologize this important point was not clear enough and clarified this point by rephrasing the respective part in the results as follows:

“Therefore, we immunized wild type mice with MOG_{35-55} peptide (B cell-independent model) or MOG_{1-117} protein (B cell-involving model) to induce EAE and discovered an increase of $CD19^+$ T cells in the immunization draining lymph nodes, comparable to that previously found for $CD20^+$ T cells (Fig. 3a-d). The increase in CD19 on T cells is more pronounced in the B cell-involving model, indirectly confirming the finding that pathogenic T cells are activated by B cells.”

Given the human data are limited to a small sample of MS and NMOSD patients, it would be interesting to add longitudinal information. Do the authors have the number and percentage of NMOSD patients' CD19+ T cells among T cells (i.e., just before the introduction of inebilizumab) or at least lymphocyte immunophenotyping at baseline with the number of total, CD4+ and CD8+ T cells. This would make possible to measure whether or not inebilizumab has a significant impact on T cells in the blood.

We thank the reviewer for raising this important question. In response, we are now providing all available details on the inebilizumab-treated patient samples. Out of the three samples we analyzed, one patient did not have any lymphocyte immunophenotyping before inebilizumab treatment. The other two patients had immunophenotyping prior to inebilizumab initiation. We provide this additional information below in table 1. However, we feel that the data is difficult to interpret, as both patients received various immunosuppressing medications before starting inebilizumab.

Table 1: Absolute amount/percentage of T cells in inebilizumab-treated NMOSD patients

	before inebilizumab	after/during inebilizumab
**patient 1**	not measured	1.91 /nl (86 %)
**patient 2**	2.32 /nl (95 %)	1.18 /nl (94 %)
**patient 3**	1.18 /nl (94 %)	1.51 /nl (92 %)

To address mechanistically whether inebilizumab is capable of depleting CD19$^+$ T cells and monocytes, we modelled *in vivo* depletion in an *in vitro* setting. For this purpose, we isolated peripheral mononuclear blood cells from three healthy controls and treated the cells with inebilizumab or an isotype control antibody *in vitro* overnight. CD19$^+$ T cells and monocytes of healthy controls seem to be reduced by inebilizumab treatment *in vitro* (Fig. 3).

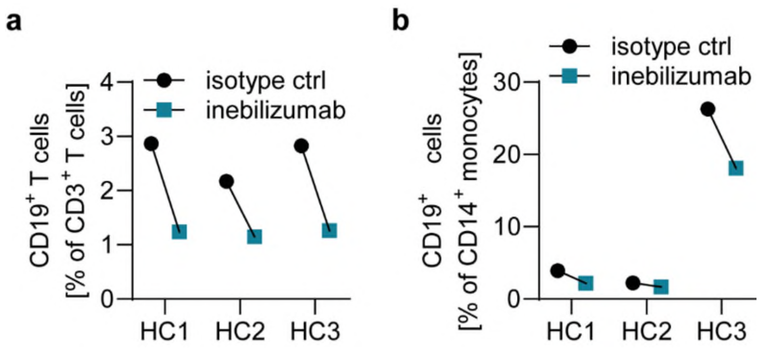

Fig. 3: *In vitro* depletion with inebilizumab
a, b Peripheral mononuclear blood cells were isolated for three healthy controls (HC) and incubated overnight with 1 µg/ml inebilizumab or an isotype control antibody *in vitro*; 2 wells were employed per HC per condition with the mean of both cultures displayed.

The authors should also discuss how such expected selective depletive therapy may impact the risk of the treatments especially on infection risk and immunosurveillance.

We agree that this important point needs to be discussed. We assume that anti-CD19 depletion of T cells and monocytes may have negative effects on infection risk and immunosurveillance, since recently B cell-activated T cells acquiring CD19 are unselectively depleted. This may include T cell populations raised to counteract infections. To include this critical point in our manuscript we added the following passage into our discussion (line 492-494):

"However, the clinical impact of anti-CD19 depleting CD19$^+$ T cells may be limited by the turnover of plasma membranes and thus the accessibility of membrane embedded CD19 on T cells. In return,

this aspect on membrane turnover could be an advantage in regard to immunosurveillance, as inebilizumab may also deplete $CD19^{+}$ T cells raised to counteract infections."

Reviewer #3:

This manuscript investigates the transfer of CD19 from B cells to T cells, utilizing both murine and human cell models. This transfer occurs via trogocytosis, a process by which membrane fragments and surface molecules are exchanged between cells in close contact. The significance of this finding lies in its implications for therapies that employ anti-CD19 antibodies to deplete B cells. Such treatments may inadvertently lead to the depletion of CD19-acquiring T cell populations.
A limitation of this manuscript is the substantial overlap with a previously published study (PMID: 35353539) by the same research team. That study described the transfer of CD20 from B cells to T cells using similar experimental methods and approaches. The current manuscript replicates the analysis to include CD19, with many of the methodologies and experimental design largely unchanged.
Nevertheless, this study does extend the concept of B cell marker trogocytosis, and its consequence for B cell-targeted therapies, to include monocytes. It also provides mechanistic insights into the process by which CD19 is transferred to monocytes.

We acknowledge that our recent manuscript provides some complementary data in regard to B cell-T cell interaction. We appreciate that the reviewer values that the current manuscript is a substantial extension on the concept of B cell trogocytosis and its consequences and the inclusion of monocytes. We thank the reviewer for this supportive comment.

The experiments conducted are of high quality and support the conclusions of the study.

Specific comments
Figure 1 and Figure 6 would benefit from inclusion of flow cytometry to show CD19 levels on B cells.

We thank the reviewer for this idea to improve our respective figures and added CD19 levels on B cells to the representative blots shown in Figure 1 (also now included in the manuscript):

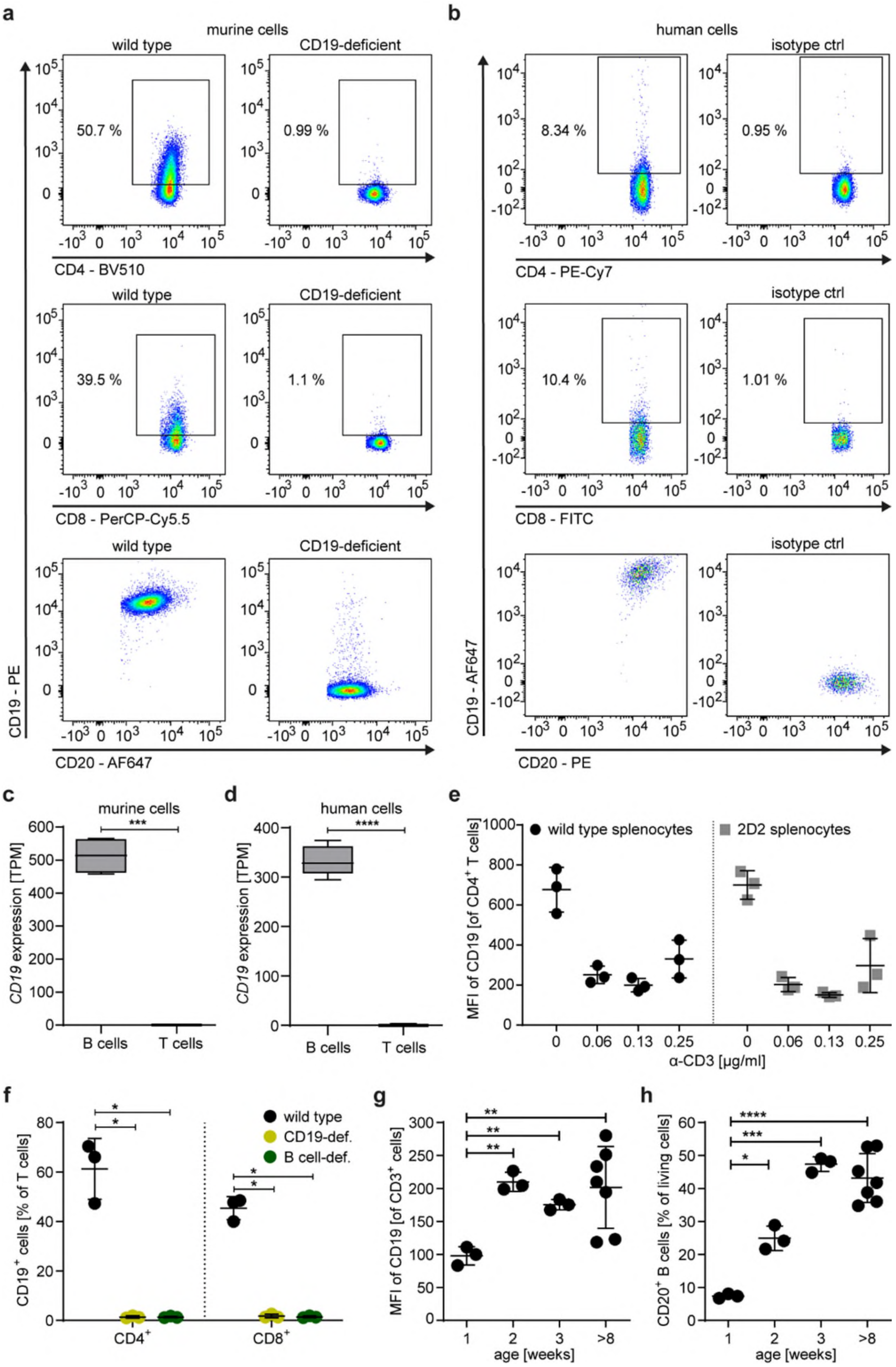

Fig.1: CD19 can be found on T cells but is not endogenously expressed

a, b Representative flow cytometric staining of CD19 on CD4+ and CD8+ T cells and B cells isolated from

a wild type and CD19-deficient CD19-cre mice or **b** healthy control peripheral blood mononuclear cells (PBMCs). **c, d** Bulk RNA sequencing of fluorescence-activated cell (FACS)-sorted B and T cells for the *CD19* transcript shown as TPM, reads per kilobase of transcript per million reads mapped; **c** n = 3-8 mice, analyzed via unpaired Student's t test with Welch's correction and Bonferroni correction for multiple testing; **d** n = 4 healthy human donors; one set of B and T cells per mouse or human, analyzed via Mann Whitney test with Bonferroni correction for multiple testing; displayed as box plot with means ± SD; **e** Mean fluorescence intensity (MFI) of CD19 of $CD4^+$ T cells of splenocyte cultures isolated from wild type mice or myelin oligodendrocyte glycoprotein $(MOG)_{35–55}$ peptide T cell receptor (TCR) transgenic 2D2 mice stimulated with anti-CD3/anti-CD28 antibodies for 48 hours. **f** Flow cytometric analysis of CD19 on $CD4^+$ and $CD8^+$ T cells from the spleens of wild type, CD19-deficient CD19-cre, and B cell-deficient µMT mice; n = 3 per group; analyzed via Brown-Forsythe and Welch ANOVA with Dunnett's T3 multiple comparisons test. **g** MFI of CD19 of $CD3^+$ T cells and **h,** percentage of $CD20^+$ B cells in murine spleens during development; n = 3-7 per group, displayed as means ± SD; analyzed via Brown-Forsythe and Welch ANOVA with Holm-Sidak's multiple comparisons test. All figures are representative of **(a, b, e-h)** or pooled from **(c, d)** 2-4 independent experiments; data displayed as means ± SD.

Figure 2. Why are %CD19+ T cells in i and j so low compared to the higher % depicted in Figure 1?

We agree that this requires clarification. The cells from Fig. 1 are stained directly after isolation from the blood. In contrast, the cells from Fig. 2i, j have gone through magnetic-activated cell sorting and were afterwards incubated with or without Staphylococcus enterotoxin B (SEB) for 48 h. The differences in CD19 positivity can be explained due to the separation process and activation of the cells. Another factor may be membrane turnover. A T cell exchanges its whole surface in the course of 24-48 h. Therefore, if T cells do not receive new CD19 via T cell-B cell interaction, they will inevitably loose it over time. Taken together these points hopefully explain the discrepancy in the amount of $CD19^+$ T cells between the signals displayed in Fig. 1 and Fig. 2.

We thank you for your kind attention and look forward to your response.

Sincerely,

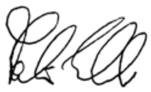

Martin S. Weber, M.D.

1. Sawen P, Lang S, Mandal P, Rossi DJ, Soneji S, Bryder D. Mitotic History Reveals Distinct Stem Cell Populations and Their Contributions to Hematopoiesis. *Cell Rep* **14**, 2809-2818 (2016).

2. Keren Z*, et al.* B-cell depletion reactivates B lymphopoiesis in the BM and rejuvenates the B lineage in aging. *Blood* **117**, 3104-3112 (2011).

RESPONSE TO REVIEWERS' COMMENTS

Reviewer #1 (Remarks to the Author):

The original manuscript by Ochs et al. lacked experiments addressing the function of B cell-derived CD19-acquired cells, which was my major concern. In the revised manuscript, the authors have adequately addressed this issue by demonstrating that anti-IgM stimulation activates CD19/IgM/IgD-acquired T cells (Fig. 5) and myeloid cells (Fig. 9). Although the in vivo pathogenic role of these cells remained to be established in an appropriate mouse model, the authors discuss this possibility appropriately based on their in vitro studies using patient-derived cells. Overall, the revised manuscript satisfactory address the major concerns raised during review.

We thank the reviewer for the concerns raised. We feel that the resulting experiments greatly improved our manuscript.

Reviewer #2 (Remarks to the Author):
I have no additional comments, thanks to the authors for the effort and the supplementary data

We thank the reviewer for their time and careful consideration of our manuscript. The requested information led to changes in the manuscript that improve comprehensibility.

Reviewer #3 (Remarks to the Author):
The revised manuscript is significantly strengthened and I support publication.

We thank the reviewer for their support and time in considering our manuscript.
